# Eco-friendly methods of synthesis and preliminary biological evaluation of sulfonamide derivatives of cyclic arylguanidines

**DOI:** 10.1016/j.ultsonch.2022.106165

**Published:** 2022-09-14

**Authors:** Przemysław Zaręba, Anna K. Drabczyk, Artur Wnorowski, Edyta Pindelska, Gniewomir Latacz, Jolanta Jaśkowska

**Affiliations:** a Faculty of Chemical Engineering and Technology, Department of Chemical Technology and Environmental Analytics, Cracow University of Technology, 24 Warszawska Street, 31-155 Cracow, Poland; b Faculty of Chemical Engineering and Technology, Department of Organic Chemistry and Technology, Cracow University of Technology, 24 Warszawska Street, 31-155 Cracow, Poland; c Department of Biopharmacy, Faculty of Pharmacy, Medical University, Lublin, Poland; d Department of Analytical Chemistry and Biomaterials, Faculty of Pharmacy, Medical University of Warsaw, 1 Banacha, 02-093 Warsaw, Poland; e Department of Technology and Biotechnology of Drugs, Jagiellonian University Medical College, 9 Medyczna Street, 30-688 Cracow, Poland

**Keywords:** Sonochemistry, Anticancer, Synthesis, Guanidines, Sulfonamides, Water

## Abstract

The chemotype of arylsulfonamide derivatives of cyclic arylguanidines is a source of molecules with valuable biological activities, including antimicrobial and antitumor properties. The methods of the synthesis presented in the literature are characterized with low selectivity and high environmental nuisance. In this publication, we present a developed alternative and earlier undescribed pathway **C**, for the synthesis of arylsulfonamide derivatives of cyclic arylguanidines (*N*-(1*H*-arylimidazol-2-yl)arylsulfonamides and *N*-(1,4-dihydroquinazolin-2-yl)arylsulfonamides), including reaction between 2-(methylsulfanyl)-benzimidazole or 2-(methylsulfanyl)-3,4-dihydroquinazoline with arylsulfonamides. We also optimized previously reported methods; **A** (reaction of 2-aminobenzimidazole or 2-amino-3,4-dihydroquinazoline with arylsulfonyl chlorides) and **B** (reaction of dimethyl-(arylsulfonyl)carbonodithioimidate with aryldiamines). The conducted research allowed achieving two independent ecological and quick methods of obtaining the desired products. We used ecological methods of ultrasound-assisted or microwave synthesis, solvent-free reactions and a “green” reaction environment. In both pathways, it has proven advantageous to use H_2_O as the solvent and K_2_CO_3_ (1 or 3 equivalent) as the basic agent. In the sonochemical variant, the efficiency reached **B**: 37–89 %, **C**: 90 % in 60 min (P = 80 W and *f* = 40 kHz), while in the microwave synthesis it was **B**: 38–74 %, **C**: 63–85 % in 0.5–4 min (P = 50 W). Path **A** led to a complementary substitution product (i.e. 1-(arylsulfonyl)-1*H*-benzimidazol-2-amine or 1-(arylsulfonyl)-1,4-dihydroquinazolin-2-amine). We obtained a small group of compounds that were tested for cytotoxicity. The **10f** (*N*-(1,4-dihydroquinazolin-2-yl)naphthalene-1-sulfonamide) showed cytotoxic activity towards human astrocytoma cell line 1321 N1. The calculated IC_50_ value was 8.22 µM at 24 h timepoint (doxorubicin suppressed 1321 N1 cell viability with IC_50_ of 1.1 µM). The viability of the cells exposed to **10f** for 24 h dropped to 48.0 % compared to vehicle control, while the cells treated with doxorubicin experienced decline to 47.5 %. We assessed its potential usefulness in pharmacotherapy in the ADMET study, confirming its ability to cross the blood–brain barrier (Pe = 5.0 ± 1.5 × 10^-6^ cm/s) and the safety of its potential use in terms of DDI and hepatotoxicity.

## Highlights

• Sonochemical method of synthesis of sulfonamide derivatives of cyclic arylguanidines.
• Microwave and sonochemical method of synthesis of sulfonamide derivatives of cyclic arylguanidines.
• Cyclic arylguanidines with antitumor activity in astrocytoma.
• Pharmacokinetic and toxicological properties of cyclic arylguanidines.

## Graphical abstract

**Figure:**
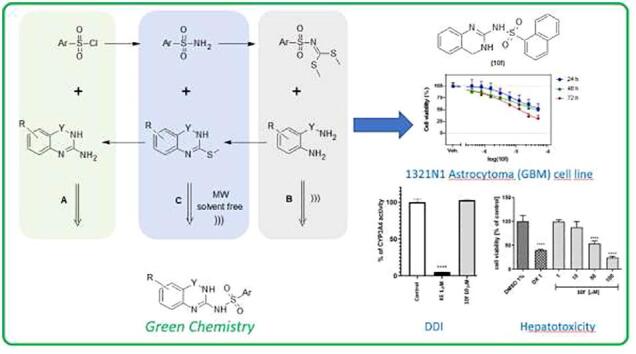

## 1 Introduction

**Fig. 1:**
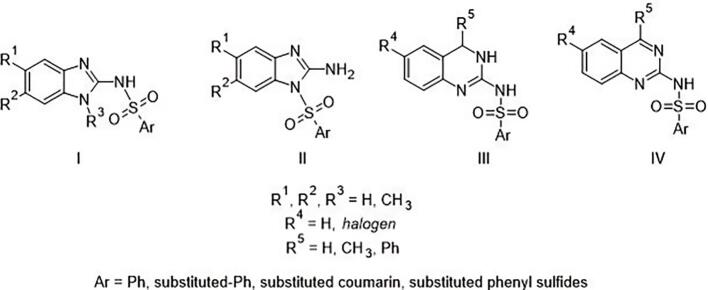
Chemical structure of arylsulfonamide derivatives of cyclic aryloguanidines [1], [2], [3], [4], [5], [6], [7], [8], [9], [10], [11], [12], [13], [14], [15], [16].

Arylsulfonamide derivatives of cyclic arylguanidines may constitute an interesting group of compounds with a potential anticancer or antimicrobial activity. These molecules have a characteristic arrangement of functional groups, including strongly basic guanidine moiety. However, it is located in the vicinity of the aryl ring and the arylsulfone group, lowering the basicity of the compounds by electron withdrawing. So far, a small number of molecules that fit to this chemotype have been described and characterized in the literature **(**Fig. 1**)**.

In group **I** (Ar = phenyl with simple substituents), compounds having activity in the treatment of parasitic diseases, such as leishmaniasis caused by *Leishmania donovani* [1], and acting as antitrypanosomal agents [2] should be distinguished. Many compounds from this group show antiviral activity [3] against polio [4], HIV (act as HIV-1 integrase inhibitors) [5] and the virus causing Marek's disease in poultry [6]. Depending on the aryl group, these compounds also act as inhibitors of 12-lipoxygenase [7], glycogen phosphorylase [8], interleukin-1 receptor-associated kinase-4 [9] and melastatin type channel modulators [10]. Molecules with coumarin derivatives as aryl groups have also been described. Among them, there are compounds with anti-breast cancer activity, connected with VEGFR-2 receptor interaction [11]. In group **II**, there are mainly inhibitors of ALDH3A1 aldehyde dehydrogenase [12], nucleotide-binding oligomerization domain (NOD) inhibitors [13], and inhibitors of NOD1-Induced Nuclear Factor-κB Activation [13]. Arylguanidine compounds exhibit cytotoxic activity against many tumor lines. These include the currently published derivatives of 2-aminobenzimidazole, showing the ability to form host: guest complexes with the porphyrin-cyclodextrin conjugate, which leads to cytotoxicity on MCF-7 cell lines [14].

Group **III** and **IV** include compounds in which substituted phenyl sulfides are present as the aryl group. Among them, there are molecules tested for anti-HIV and antitumor activity, using cell lines derived from nine different cancer types (lung, colon, prostate, breast, renal, ovarian, CNS cancers, and melanoma and leukemia). Most of the tested compounds were inactive in HIV-related assays, but exhibited a moderate (IC_50_/GI_50_ < 50) to high (IC_50_/GI_50_ < 10) anticancer activity against some human cell lines [15], [16].

Despite the promising reports on the biological activity of these compounds, the number of cyclic arylguanidine sulfonamide derivatives described in the literature is still relatively small. In particular, the previously postulated antitumorigenic activity [11], [15], [16], possibly originating from enzyme inhibition (e.g., blockage of protein kinases responsible for growth signaling propagation), seems to be of interest.

There are some ambiguities and limitations in the literature regarding the preparation of sulfonamide derivatives of cyclic arylguanidines. The two main methods of synthesizing have been described in the earlier publications. However, both are highly harmful to the environment. The reaction of 2-aminobenzimidazole (**1a**) with benzenesulfonyl chloride (**2a**) is proposed as the main method (**A**) for the synthesis of compounds from groups **I** and **II**. However, in this reaction, a mixture consisting of three different products can be obtained. At first glance, there is a visible dependence between the direction of the substitution and the solvent used for the synthesis. However, the literature data are not entirely consistent, which is presented in Table 1. The presented results may suggest low selectivity of this method and difficulties in controlling the direction of the substitution.

**Table 1:** Reaction of 2-aminobenzimidazole (**1a**) with benzenesulfonyl chloride (**2a**), described in the literature [3], [14], [17], [18], [19], [20].


Ref	Ratio **1a**:**2a**	Base	Solvent; mass [%]	Conditions	Time	Product content*		
						**10a**	**11a**	**12a**
[3]	1:1	NaOH; 2.2 Eq	MeCN:H_2_O 10:1; 86 %	RT	4 h	–	100 %	–
[14]	1:1.03	–	Pyridine; 56 %	RT	12 h	–	35 %	–
[17]	1:1.1	TEA; 3 Eq	CH_2_Cl_2_; 70 %	RT; DMAP; 0.1 Eq	2 d	100 %	–	–
[18]	1:1	TEA; 1 Eq	Acetone; 92 %	RT	4 h	–	80 %	–
[19]	1:1	–	Pyridine; 72 %	50 °C	1 h	–	35 %	38 %
[19]	1:1	–	Pyridine; 72 %	50 °C	168 h	40 %	–	–
[20]	1:1	–	Pyridine	Reflux	30 min	84 %	–	–

*Determined by yield of isolated product, or chromatography, dependent on reference.

The second described method (**B**) can be used to obtain compounds from groups **I** and **III**. In this case, the corresponding aryldiamine (**3a–d**) is reacting with an *N*-derivative of the arylsulfonamides (**4a–f**). The alkylating agent can be dimethyl-(benzenesulfonyl)carbonodithioimidate (**4a**), (benzenesulfonyl)carbonimidoyl (**4d**), and *N*-(diaminomethylidene)benzenesulfonamide (**4e**). The use of **4d** as an alkylating agent made it possible to obtain the product with a high yield [21]. However, this reaction requires the use of aromatic solvents in large amounts. Moreover, attention should be paid to the multi-step pathway of substrate synthesis (**4d**) (Table 2).

**Table 2:** Reaction of aryldiamines (**3a-d**) with an *N*-derivative of the arylsulfonamides (**4a-e**), described in the literature. [21], [22], [23], [24], [25]^.^


Ref	Y	Ar	Z	Ratio 3:4	Base	Solvent; mass [%]	Conditions	Time	Yield [%]
[21]	–	Ph	Cl	1:1	–	Benzene; 93 %	Reflux	5 h	72 %
[22]	–	Ph	NH_2_	1:1	–	–	190 °C + 215 °C	2.5 + 2.5 h	56 %
[23]	–	Ph	SCH_3_	1:1	–	DMF; 74 %	Reflux	16 h	69 %
[24]	–	Ph	SCH_3_	1:1.1	K_2_CO_3_; 1.5 Eq	H_2_O/EtOH 3:1; 86 %	Reflux; HTAB 0.1 Eq	1 h	83 %
[25]	CH_2_	4-CH_3_-Ph	SCH_3_	1:1	–	DMF	Reflux	–	56 %

The reaction with **4e** is an alternative to the substrate **4d**. This reaction does not require the use of a solvent; however, it is carried out at high temperature and has a low yield [22]. The most interesting pathway seems to be the reaction between diamine (**3a–d**) and dimethyl-(benzenesulfonyl)carbonodithioimidate (**4a–c**). The reactions described in the literature were carried out in a DMF [23], [25], or H_2_O/EtOH 3:1 system [24]. The latter variant, in which the yield of the product obtained was 83 %, deserves particular attention due to mild synthesis conditions and a high yield.

**Scheme 1:**
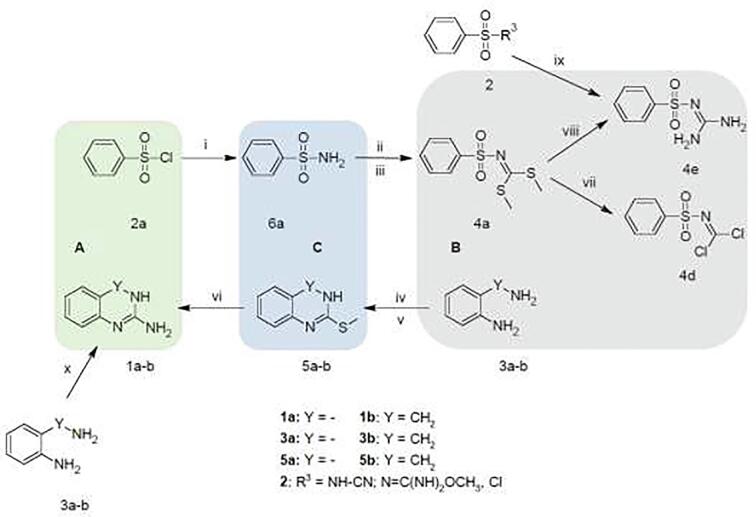
Synthesis of substrates i) NH_3_, acetone [26], ii) CS_2_, KOH, DMF [26], iii) CH_3_I, 2 equiv. [26], iv) CS_2_, EtOH [27], v) CH_3_I, 1 equiv. [27], vi) NH_3_, acetone [27], vii) Cl_2_[28], viii) NH_3_[29], ix) NH_3_ for *R*^3^ = NH-CN or N=C(NH_2_)OCH_3_, guanidine for *R*^3^ = Cl [30], x) BrCN [31].

It should be noted that the substrates used in both syntheses are related to each other according to Scheme 1.

If the basic substrates, arylsulfonylchloride (**2**) and diamine (**3**), are considered, methods **A** and **B** (using dimethyl-(benzenesulfonyl)carbonodithioimidate (**4a–c**)) are equal in terms of the number of steps and primary substrates used. However, attention should be paid to the inferior atomic economy of variant **B** due to the need of the doubled amount of iodomethane in step iii of the synthesis of substrate **4a** compared to method **A** (step iv) of the synthesis of substrate **1a**.

Considering the potential medical use of the compounds from the group of sulfonamide derivatives of cyclic arylguanidines, it is justified to work on a new, universal, and fast method of the synthesis. Due to the problematic course of the synthesis methods described in the literature and high environmental nuisance resulting from the long reaction time, the large number of solvents used, the aggressive reaction environment, low selectivity, and atomic economy, we decided to develop new, more ecological approaches to the synthesis of the compounds from the described group.

Based on substrate synthesis Scheme 1, we decided to check and optimize the **A** (green) and **B** (gray) synthesis paths with unconventional methods of conducting the reaction. We also decided to develop a completely new synthesis pathway **C** (blue), which involved a reaction between intermediates **5** and **6** used for the synthesis of the substrates in methods **A** and **B**. This method is supposed to be characterized by more favorable atomic economy than method **B** and greater selectivity of the obtained product than method **A** (for the preparation of compounds from groups **I** and **III**), which will improve the ecological values of the synthesis. As part of the preliminary biological tests, some of the compounds were tested for cytotoxicity against selected lines of breast cancer and glioblastoma. The most active compound was characterized in terms of its pharmacokinetic properties [32].

## 2 Materials and methods

### 2.1 Chemical synthesis

The ultrasound reactions ( **)))** ) were carried out in the Ultrasonic bath PS-08, with the ultrasound power 80 W and frequency 40 kHz. The microwave-assisted reactions (**MW**) were carried out in the CEM Discover microwave reactor. All chemicals were purchased from Sigma-Aldrich and all solvents used in the synthesis were from POCH. Thin-layer chromatography (TLC) was performed using chloroform:methanol eluent, in the ratio from 975:25 to 9:1 on Sigma-Aldrich sheets (silica gel on aluminum, with a fluorescent indicator 254 nm, 200 µm layer thickness, 60 Å pore diameter, 8.0–12.0 µm particle size). UV light with a wavelength of 254 nm was used for the analysis. High-performance liquid chromatography (HPLC) was performed on a PerkinElmer Series 200 HPLC (Symetry C-18, 5 µm seed size, 3.9 × 150 mm) column and MeOH:H_2_O 4:6 eluent acidified with 0.1 % formic acid. The melting points were measured with a Boëtius apparatus. IR spectra were taken on an FTS-165 spectrometer. NMR spectra were recorded on a Bruker Avance 400 MHz spectrometer, using TMS as an internal reference. Detailed description of NMR spectra is provided in the supplementary file. The LC-MS system consisted of a Waters Acquity UPLC system coupled to a Waters TQD mass spectrometer (electrospray ionization mode ESI-tandem quadrupole). The analyses were carried out with an Acquity UPLC BEH C-18, 1.7, 2.1 × 100 mm column. Elemental analysis was performed on the Vario EL II apparatus.

Benzenesulfonyl chloride, naphthalene-1-sulfonyl chloride, naphthalene-2-sulfonyl chloride, benzene-1,2-diamine, 2-(aminomethyl)aniline, 4-chlorobenzene-1,2-diamine, 2-(aminomethyl)-4-chloroaniline, 1*H*-benzimidazol-2-amine were purchased from suppliers. Benzenesulfonamide, naphthalene-2-sulfonamide, naphthalene-1-sulfonamide were prepared according Reddy et al. procedure [33]. 2-chloro-1*H*-benzimidazole, 2,5-dichloro-1*H*-benzimidazole were prepared according Kilchmann et al. procedure [34]. 2-(methylsulfanyl)-1*H*-benzimidazole, 2-(ethylsulfanyl)-1*H*-benzimidazole were prepared according Rodríguez et al. procedure [35]. 2-(methanesulfonyl)-*1H*-benzimidazole was prepared according Hoggarth procedure [36]. 2-(methylsulfanyl)-1,4-dihydroquinazoline, 6-chloro-2-(methylsulfanyl)-1,4-dihydroquinazoline, 1,4-dihydroquinazolin-2-amine were prepared according Zeiger et al. procedure [27]. 6-chloro-1*H*-benzimidazol-2-amine was prepared according Leonard et al. procedure [37]. Dimethyl(benzenesulfonyl)carbonodithioimidate, dimethyl(naphthalene-2-sulfonyl)carbonodithioimidate, dimethyl(naphthalene-1-sulfonyl)carbonodithioimidate were prepared according Loevezijn et al. procedure [26].

#### 2.1.1 Method A

A mixture of 0.001 mol of aryl sulfochloride **2a**, 0.001 mol of amine **1a** was prepared in a round bottom flask. The mixture was dissolved in a suitable solvent (78–80 % mass; 1.2–1.7 ml). The reactions were carried out for 30 s in the MW reactor while monitoring their progress on TLC. After this time, a sample was taken for analysis.

#### 2.1.2 Method B

A mixture of 0.001 mol diamine **3a–d**, 0.001/0.0012 mol dimethyl(arylsulfonyl)dithioimidocarbonate (**4a–c**) 1/1.5/3 Eq of the appropriate basic agent and 30–95 % mass of the solvent was placed in a round bottom flask. For solvent-free reactions, the mixture was triturated in a mortar and transferred to a round bottom flask, then whipped with a stirring rod. The 0.1 Eq TBAB was also added in some variants. The flask was placed in the MW reactor and the reaction was carried out for 1–5 min, heated for 2–8 h or placed in an ultrasonic bath and the reaction was carried out for 20–60 min. Due to the possibility of **3a–b** decomposition, care was taken that the reaction mixture did not get hotter than 150 °C. After this time, a sample was taken for analysis. Then, 5 ml of water was added to the reaction mixture and the resulting product was filtered off.

#### 2.1.3 Method C

A mixture of 0.001 mol of the **5a–g** (alkylating agent), 0.001 mol of arylsulfonamide **6a–c**, 1–3 Eq of the appropriate base and 5–90 % by weight of the solvent (or solvent-free variant) was placed in a round bottom flask. In some cases, 0.1 Eq of TBAB was also added. The reaction mixtures were heated (in the case of the solvent-free variant without stirring) in an oil bath for 3/20/48 h at the temperature of 130/180/200/230 °C, carried out in a MW reactor for 0.5–40 min or placed in an ultrasonic bath and the reaction was carried out for 20–60 min. After this time, samples were taken for analysis. After cooling, water was added to the reaction mixture and stirred for 30 min. Then, the resulting precipitate was filtered off.

***N*-(1*H*-benzimidazol-2-yl)benzenesulfonamide 10a**.

White solid, yield = 74 % (meth. B). FT-IR: 3372 (N—H, Str), 3057 (C—H Ar, Str), 1625; 15,979 (C=C Ar, Str), 1290(S=O, Str). Formula weight: 273.31, UPLC-MS: MW – 274.14, purity = 98 %, R_t_ – 4.45, m_p_ > 300 °C Anal. Calcd for C_13_H_11_N_3_O_2_S: C, 57.13; H, 4.06; N, 15.37; S, 11.73. Found: C, 56.93; H, 4.04; N, 15.38; S, 12.01.

**1-(benzenesulfonyl)-1*H*-benzimidazol-2-amine 11a**.

White crystals, yield = 71 % (meth. A). ^15^N NMR (500 MHz, DMSO) *δ* 316.2 N^12^, 214.2 N^1^, 182.5 N^3^. FT-IR: 3428 (N—H, Str), 3270 (N—H, Str, II^nd^ amine), 3081 (C—H Ar, Str), 1658; 1588 (C=C Ar, Str), 1366(S=O, Str). Formula weight: 273.31, UPLC-MS: MW – 274.14, purity = 100 %, R_t_ – 4.89, m_p_ = 196 – 198 °C. Anal. Calcd for C_13_H_11_N_3_O_2_S: C, 57.13; H, 4.06; N, 15.37; S, 11.73. Found: C, 56.93; H, 4.04; N, 15.38; S, 12.01.

**1-(naphthalene-1-sulfonyl)-1*H*-benzimidazol-2-amine 11b**.

Beige solid, yield = 62 % (meth. A). FT-IR: 3442 (N—H, Str), 3304 (N—H, Str, IInd amine), 3061 (C—H Ar, Str), 1654; 1557 (C=C Ar, Str), 1355(S=O, Str), Molecular formula: C_17_H_13_N_3_O_2_S, Formula weight: 323.37, UPLC-MS: MW – 324.21, purity = 100 %, R_t_ – 5.94, m_p_ > 300 °C. Anal. Calcd for C_17_H_13_N_3_O_2_S: C, 63.14; H, 4.05; N, 12.99; S, 9.91. Found: C, 63.42; H, 4.13; N, 12.96; S, 9.97.

**1-(naphthalene-2-sulfonyl)-1*H*-benzimidazol-2-amine 11c**.

White solid, yield = 54 % (meth. A). FT-IR: 3443 (N—H, Str), 3317 (N—H, Str, IInd amine), 3060 (C—H Ar, Str), 1659; 1557 (C=C Ar, Str), 1364(S=O, Str). Formula weight: 323.37, UPLC-MS: MW – 324.21, purity = 86 %, R_t_ – 6.03, m_p_ = 188 – 194 °C. Anal. Calcd for C_17_H_13_N_3_O_2_S: C, 63.14; H, 4.05; N, 12.99; S, 9.91. Found: C, 63.42; H, 4.25; N, 12.96; S, 9.90.

**5-chloro-1-(naphthalene-1-sulfonyl)-1*H*-benzimidazol-2-amine 11d**.

White solid, yield = 41 % (meth. A). FT-IR: 3435 (N—H, Str), 3309 (N—H, Str, II^nd^ amine), 3081 (C—H Ar, Str), 1658; 1556 (C=C Ar, Str), 1356(S=O, Str), 765 (C-Cl, Str). Formula weight: 357.81, UPLC-MS: MW – 358.14, R_t_ – 7.09, purity = 100 %, m_p_ = 225 – 229 °C. Anal. Calcd for C_17_H_12_ClN_3_O_2_S: C, 57.07; H, 3.38; N, 11.74; S, 8.96. Found: C, 56.99; H, 3.35; N, 11.79; S, 8.91.

***N*-(1,4-dihydroquinazolin-2-yl)benzenesulfonamide 10e**.

White solid, yield = 74 % (meth. B), 78 % (meth. C). FT-IR: 3332 (N—H, Str), 3189 (N—H, Str), 3029 (C—H Ar, Str), 1604; 1534 (C=C Ar, Str), 1362(S=O, Str). Formula weight: 287.34, UPLC-MS: MW – 288.16, purity = 100 %, R_t_ – 5.15, m_p_ = 182 – 186 °C. Anal. Calcd for C_14_H_13_N_3_O_2_S: C, 58.52; H, 4.56; N, 14.62; S, 11.16. Found: C, 58.56; H, 4.66; N, 14.66; S, 11.31.

***N*-(1,4-dihydroquinazolin-2-yl)naphthalene-1-sulfonamide 10f**.

White solid, yield = 81 % (meth. B), 76 % (meth. C). FT-IR: 3375 (N—H, Str), 3300 (N—H, Str), 3056(C—H Ar, Str), 1608; 1538 (C=C Ar, Str), 1369(S=O, Str). Formula weight: 337.40, UPLC-MS: MW – 338.20, purity = 100 %, R_t_ – 6.09, m_p_ – 182 – 186 °C. Anal. Calcd for C_18_H_15_N_3_O_2_S: C, 64.08; H, 4.48; N, 12.45; S, 9.5. Found: C, 64.26; H, 4.57; N, 12.43; S, 9.64.

***N*-(1,4-dihydroquinazolin-2-yl)naphthalene-2-sulfonamide 10g**.

White solid, yield = 89 % (meth. B), 85 % (meth. C). FT-IR: 3314 (N—H, Str), 3254 (N—H, Str), 3030(C—H Ar, Str), 1634; 1539 (C=C Ar, Str), 1363(S=O, Str). Formula weight: 337.40, UPLC-MS: MW – 338.20, purity = 99 %, R_t_ – 6.13, m_p_ > 300 °C. Anal. Calcd for C_18_H_15_N_3_O_2_S: C, 64.08; H, 4.48; N, 12.45; S, 9.5. Found: C, 64.81; H, 4.54; N, 12.56; S, 9.59.

***N*-(6-chloro-1,4-dihydroquinazolin-2-yl)naphthalene-2-sulfonamide 10h**.

White solid, yield = 84 % (meth. B), 69 % (meth. C). FT-IR: 3313 (N—H, Str), 3055 (C—H Ar, Str), 1621; 1537 (C=C Ar, Str), 1363(S=O, Str), 765 (C-Cl, Str),Molecular formula: C_18_H_14_ClN_3_O_2_S, Formula weight: 371.84, UPLC-MS: MW – 372.19, purity = 100 %, R_t_ – 6.76, m_p_ = 207 – 209 °C. Anal. Calcd for C_18_H_1C_ClN_3_O_2_S: C, 58.14; H, 3.80; N, 11.30; S, 8.62. Found: C, 58.19; H, 3.83; N, 11.39; S, 8.67.

### 2.2 Molecular modeling

All calculations were carried out using Density Functional Theory as implemented in the Jaguar suite of *ab initio* quantum chemistry programs [38]. All intermediate and transition-state geometries were optimized with M06-2X [39] functional and the 6-31G** [40] basis sets. The optimization was carried out without taking into account the solvation due to the reaction conditions. Initial Hessian was calculated using quantum chemical methods. Fukui indices were also determined for the substrates [41].

To locate transition states, the potential energy surface was first explored approximately using the standard method, followed by the quadratic synchronous transit (QST) [42] search that uses the standard transition state as an initial guess. In the QST, the initial part of the transition state search is restricted to a circular curve connecting the reactant, initial transition state guess, and the product, followed by a search along the Hessian eigenvector that is the most similar to the tangent of this curve.

Frequency calculations were performed to verify the correct nature of the stationary points and to estimate zero-point energy (ZPE) and thermal corrections to thermodynamic properties. Intrinsic reaction coordinate (IRC) calculations [43] were employed to locate reagent and product minima connected with the transition states for each considered reaction step.

The optimized geometries characterized as the local minima on the potential energy surfaces do not contain any imaginary frequency, while each of the transition states contain one imaginary frequency.

### 2.3 Cytotoxicity assessment

The cytotoxic properties of newly synthetized compounds were assessed in human astrocytoma cell line 1321 N1 (RRID:CVCL_0110, European Collection of Authenticated Cell Cultures, ECACC:86030402) and human breast adenocarcinoma cell line MDA-MB-231 (RRID:CVCL_0062; ATCC:HTB-26). ECACC and ATCC perform thorough cell line authentication utilizing Short Tandem Repeat (STR) profiling. Upon receipt of the cell lines, the cells were expanded for a few passages to enable the generation of new frozen stocks. The cells were resuscitated as needed and used for<6 months after resuscitation (no>15 passages). Cell line 1321 N1 was cultured in high-glucose (4500 mg/L) Dulbecco's modified Eagle's medium (DMEM) supplemented with 10 % fetal bovine serum (FBS). The MDA-MB-231 cells were maintained in RPMI-1640 with 10 % FBS. All cell culture media were additionally fortified with penicillin (100 U/mL), streptomycin (100 µg/mL), and amphotericin B (250 ng/mL) to prevent bacterial and yeast contamination. The cell culture media and supplements used in this study were purchased from Sigma-Aldrich. The cells were maintained at 37 °C in a humidified 5 % incubator in an atmosphere of 5 % CO_2_ and 95 % air.

The cells were subcultured upon reaching the confluency of about 80 %. Cell detachment was facilitated with TrypLE solution (Thermo Fisher Scientific). The cells were counted in a Z2 particle counter (Beckman Coulter), then plated at 5,000 cells/well in 96-well plates (Eppendorf) in 100 µL of full growth medium and cultured overnight to enable cell attachment. Next day, the cells were subjected to either vehicle (0.1 % DMSO) or our compounds of interest for 24, 48, or 72  h. The assessment of cell viability was performed by adding 20 µL/well of the mixture of 3-(4,5-dimethylthiazol-2-yl)-5-(3-carboxymethoxyphenyl)-2-(4-sulfophenyl)–2*H*-tetrazolium, inner salt (MTS) and phenazine ethosulfate from Promega (#G3580). After 3 h of incubation, the absorbance was recorded at 490 nm using Synergy H1 plate reader (Biotek) The experiments were carried out in quadruplicates and were repeated three times. The obtained values were normalized to vehicle control and plotted.

The IC_50_ values were calculated with GraphPad Prism 8.0.1 software (nonlinear regression, log(inhibitor) vs normalized response) as the dose that causes a 50 % decrease in cell viability relative to the maximum inhibition observed. Statistical significance determination was evaluated with GraphPad Prism 5.0.1 software using one-way ANOVA, followed by Bonferroni’s comparison test (*p* < 0.05).

### 2.4 ADMET tests

The ADME-Tox parameters of **10f** were analyzed according to described previously protocols [44], [45] and included: Parallel Artificial Membrane Permeability Assay (PAMPA) passive permeability testing, the influence on CYP3A4 activity, metabolic stability in mouse liver microsomes, and hepatotoxicity assessment with HepG2 cells.

#### 2.4.1 PAMPA test

The pre-coated PAMPA Plate System Gentest was sourced from Corning (Tewksbury, MA, USA). The **10f** and caffeine solutions (200 µM) were prepared in a PBS buffer (pH = 7.4) and then added to a PAMPA plate. The plate was incubated at room temperature for 5 h without stirring. Then, 50 μL was aspirated from both Acceptor (A) and Donor (D) wells and then diluted with a 50 µL solution of an internal standard (IS). The concentrations of the tested compounds in the A and D wells were estimated with an LC/MS Waters ACQUITY™ TQD system with the TQ Detector (Waters, Milford, USA). The Pe values were estimated according to the proper formulas provided by Corning and described previously in the literature [46].

#### 2.4.2 Drug-drug interactions

The influence on CYP3A4 activity by **10f** was analyzed with luminescent CYP3A4 P450-Glo (Madison, WI, USA). The compounds were tested in triplicate in a final concentration 10 μM. The luminescent signal was measured with the EnSpire PerkinElmer (Waltham, MA, USA) microplate reader. Reference inhibitor ketoconazole (KE) was obtained from Sigma-Aldrich (St. Louis, MO, USA).

### 2.5 Metabolic stability

The metabolic stability of **10f** was estimated with mouse liver microsomes (MLMs) (Sigma-Aldrich, St. Louis, MO, USA). The tested compound (50 µM) was incubated in the presence of MLMs (1 mg/ml) for 120 min in a buffer (10 mM Tris–HCl buffer at 37 °C). Then, to terminate the reaction, cold methanol was added to the reaction mixture. The precipitated MLMs were centrifuged and the supernatant was analyzed with a LC/MS Waters ACQUITY™ TQD system with the TQ Detector (Waters, Milford, USA).

### 2.6 Hepatotoxicity

Hepatotoxicity was evaluated by means of an MTS assay described above (section 23) with a HepG2 human hepatoma cell line (ATCC:HB-8065) [47], [48]. In brief, the cells were incubated for 48 h at 96-well plates with our compound of interest (concentration range: 1–100 μM), or doxorubicin (DX, 1 µM; Sigma-Aldrich), which served as control. Compound **10f** was tested in a single experiment in quadruplicate.

### 2.7 Statistical analysis

The statistical significance determination was evaluated with GraphPad Prism 5.0.1 software using one-way ANOVA, followed by Bonferroni’s comparison test: *p* < 0.001 for DDI and hepatotoxicity, and *p* < 0.05 for other tests.

## 3 Results and discussion

**Fig. 2:**
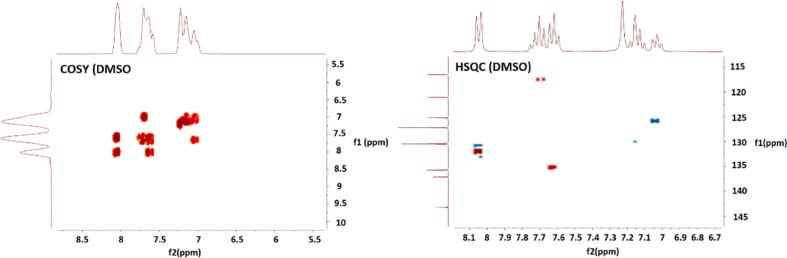
NMR spectra of product obtained in 1^st^ reaction in COSY and HSQC techniques.

First, we repeated the synthesis of compound **10a** in accordance with the preparative recipe presented in [17] and we characterized the product structurally in order to obtain a reference compound for the development of the synthesis method. One product was obtained in this reaction, and after separation from the reaction mixture, it was subjected to structural analysis. The obtained compound was 100 % pure. Its UPLC-MS analysis made it possible to confirm the molar mass. In the next step, it was subjected to a detailed NMR analysis, measuring the signal of ^1^H, ^13^C, ^15^N nuclei and using the COSY and HSQC techniques (Fig. 2).

The performed analyses showed that in this reaction we selectively obtained product **11a**, which was also confirmed by the measured melting point. The said method was also tested for the synthesis of structural derivatives of compound **11a** having a substituent on the aryl ring in the 2-aminobenzimidazole (**11d**) and naphthalene analogs of aryl sulfonylchlorides (**11b–c**). In each case, the ring substitution product was obtained selectively. This method turned out to be non-selective in the case of preparation of 2-arylsulfonamide derivatives of 1,4-dihydroquinazoline (**11e–f**), which gave a complex mixture of products **10e–f**, **11e–f** and high molecular by-products, including disubstitution products **12e–f**.

In the next step, an attempt was made to synthesize compound **10a** and its naphthalene analogues (**10b–c**) in a reaction in the presence of pyridine, according to [19]. Interestingly, for **10a**, 28 % of the desired product was observed in the reaction mixture, while the content of 72 % was disubstituted product **12a**. However, in the case of the naphthalene derivatives, no product **10b–c** was observed, almost only disubstitution product **12b–c** and unreacted starting material. We made an attempt to develop our own method of the synthesis of compound **10a** in a sonochemical or MW-assisted reactions in the presence of various solvents. Using DMF, or solvent-free reactions, only product **12a** was obtained. In the variant using pyridine, or acetone, ring-substituted product **11a** was obtained selectively. On the basis of the performed reactions, it can be concluded that the indicated synthetic pathway is not suitable for the preparation of the product **10** (Table 3).

**Table 3:** Development the method **A** of synthesis.


No	Y	Ar	R	Ratio of **1:2**	Base	Solvent; mass [%]	Conditions	Time	Product content*			Yield isol
									**10**	**11**	**12**	
1	–	Ph	H	1:1.1	TEA; 3 Eq	CH_2_Cl_2_; 70 %	RT; DMAP 0.1 Eq	2d	–	100 %	–	71 %
2	–	1-napht	H	1:1.1	TEA; 3 Eq	CH_2_Cl_2_; 70 %	RT; DMAP 0.1 Eq	4d	–	100 %	–	62 %
3	–	2-napht	H	1:1.1	TEA; 3 Eq	CH_2_Cl_2_; 70 %	RT; DMAP 0.1 Eq	2d	0.7 %	86 %	10 %	54 % (**11**)
4	–	1-napht	Cl	1:1.1	–	CH_2_Cl_2_; 70 %	RT; DMAP 0.1 Eq	2d	–	64 %	–	41 %
5	CH_2_	Ph	H	1:1.1	TEA; 3 Eq	CH_2_Cl_2_; 70 %	RT; DMAP 0.1 Eq	2d	7 %	21 %	53 %	–
6	CH_2_	1-napht	H	1:1.1	TEA; 3 Eq	CH_2_Cl_2_; 70 %	RT; DMAP 0.1 Eq	2d	11 %	20 %	–	–
7	–	Ph	H	1:1	–	Pyridine; 72 %	50 °C	1 t	28 %	–	72 %	–
8	–	2-napht	H	1:1	–	Pyridine; 72 %	50 °C	1 t	–	–	100 %	–
9	–	1-napht	H	1:1	–	Pyridine; 72 %	50 °C	1 t	–	17 %	77 %	–
10	–	Ph	H	1:1	–	DMF; 90 %	RT	1d	–	–	100 %*	–
11	–	Ph	H	1:1	–	DMF; 78 %	MW 100 W	30 s	–	–	100 %*	–
12	–	Ph	H	1:1	–	Pyridine; 80 %	MW 100 W	30 s	–	100 %*	–	–
13	–	Ph	H	1:1	–	Acetone; 80 %	)))	30 s	–	100 %*	–	–
14	–	Ph	H	1:1	–	–	MW 100 W	30 s	–	–	100 %*	–

*Determined by comparison with references on TLC, MW – reaction in microwave reactor, ))) – ultrasound-assisted reaction.

In the next step, an attempt was made to synthesize compound **10** according to the path shown in Table 2. First, the reactions were carried out in accordance with the variants described in the literature, in the presence of DMF, or with the use of K_2_CO_3_ in DMF, ethanol and the system ethanol:water/1:3 in the presence of HTAB. Interestingly, in each of the variants mentioned, the content of product **10a** did not exceed 8 %. In the post-reaction mixture, mainly unreacted diamine **3a** and partially decomposed **4a** were observed. About 8 % of the product **10a** was found using the DMF/NaOH system, however, the use of a stronger base increased the degree of **4a** decomposition into by-products. Similar observations were made for the chlorine-substituted *o*-phenylenediamine (**3c**) and the naphthyl derivatives (**4b–c**). Interesting results were also obtained by carrying out the reaction under MW irradiation. The use of ethanol, or water in the presence of K_2_CO_3_ made it possible to obtain the product with a satisfactory yield. Importantly, the use of the K_2_CO_3_/H_2_O system allowed obtaining only 8 % of the product, but the addition of the TBAB, while maintaining the remaining parameters, increased the product content to 83 %, achieving an isolated product yield of 74 %. The use of DMF, or a stronger basic agent, as with conventionally conducted reactions, led to product formation, but also, to a large extent, to the breakdown of the substrates into many by-products. Interestingly, **10a** was also obtained under solvent-free conditions using TBAB as a phase-transfer catalyst.

Much more satisfactory effects were obtained for dihydroquinazoline derivatives (**10e–i**). By heating the starting materials in ethanol, a high yield of the product (82 %) was obtained after just 3  h. However, reducing the mass fraction of the solvent from 90 % to 40 % brought the yield down to just 21 %. High efficiency comparable with the first variant was observed when water was used as a solvent. To shorten the reaction time and reduce the solvent content, an attempt was made to synthesize the product in the presence of MW irradiation or ultrasounds using a 40 % of solvent. In the MW variant, after 1 min of heating with the use of water, 38 % of the product was obtained in the post-reaction mixture; the rest was mainly unreacted starting materials. When the reaction was continued, it unfortunately led to their decomposition, disproportionately to the amount of the new product formed. In the ultrasonic variant, 64 % of the product in the reaction mixture was observed after 40 min of the reaction. The rest was unreacted substrates. Importantly, when the reaction was continued for another 20 min, it led to the almost complete disappearance of the starting materials. The use of water as a solvent brought much better results compared to ethanol. In this method, several derivatives of compound **10a** were prepared (Table 4).

**Table 4:** Development the method **B** of synthesis.

No.	Z = SCH_3_			Ratio of **3:4**	Base	Solvent; mass [%]	Conditions	Time	Product content*	
	R	Ar	Y						LC	Yield isol
1	H	Ph	–	1:1	–	DMF; 74 %	Reflux	24 h	0 %	–
2	H	Ph	–	1:1	K_2_CO_3_; 1 Eq	DMF; 95 %	Reflux	2 h	0 %*	–
3	H	Ph	–	1:1	K_2_CO_3_; 1 Eq	EtOH; 95 %	Reflux	4 h	0.5 %	–
4	H	Ph	–	1:1	K_2_CO_3_; 1 Eq	EtOH:H_2_O; 1:3; 88 %	Reflux; HTAB 0.1 Eq	2 h	6 %	–
5	H	Ph	–	1:1	NaOH; 1 Eq	DMF; 90 %	Reflux	8 h	7.3 %*	–
6	H	Ph	–	1:1	K_2_CO_3_; 1 Eq	DMF; 30 %	MW 50 W	3 min	0.5 %	–
7	H	Ph	–	1:1	K_2_CO_3_; 3 Eq	DMF; 60 %	MW 50 W	4 min	57 %	–
8	H	Ph	–	1:1	K_2_CO_3_; 3 Eq	EtOH; 60 %	MW 50 W	4 min	60 %	48 %
9	H	Ph	–	1:1	K_2_CO_3_; 3 Eq	H_2_O; 60 %	MW 50 W	4 min	8 %	–
10	H	Ph	–	1:1	DBU; 1.5 Eq	DMF; 50 %	MW 50 W	3 min	54 %	–
11	H	Ph	–	1:1	NaOH; 3 Eq	H_2_O; 60 %	MW 50 W	5 min	25 %	–
12	H	Ph	–	1:1	K_2_CO_3_; 3 Eq	H_2_O; 60 %	MW 50 W; TBAB 0.1 Eq	5 min	83 %	74 %
13	H	Ph	–	1:1	K_2_CO_3_; 3 Eq	–	MW 50 W; TBAB 0.1 Eq	5 min	57 %	44 %
14	H	Ph	–	1:1	K_2_CO_3_; 3 Eq	H_2_O; 80 %	))); TBAB 0.1 Eq	60 min	37 %	–
15	H	Ph	–	1:1	K_2_CO_3_; 1 Eq	H_2_O; 90 %	))); TBAB 0.1 Eq	60 min	27 %	–
16	H	2-napht	–	1:1.2	K_2_CO_3_; 1 Eq	EtOH; 95 %	Reflux	4 h	0 %*	–
17	Cl	1-napht	–	1:1.2	K_2_CO_3_; 1 Eq	EtOH; 95 %	Reflux	4 h	0 %*	–
18	H	1-napht	–	1:1	NaOH; 1 Eq	DMF; 80 %	Reflux	6 h	1 %*	–
19	H	1-napht	–	1:1.2	K_2_CO_3_; 1 Eq	EtOH; 95 %	Reflux	4 h	0 %*	–
20	H	Ph	CH_2_	1:1	K_2_CO_3_; 1 Eq	EtOH; 90 %	Reflux	3 h	56 %*	48 %
21	H	Ph	CH_2_	1:1	K_2_CO_3_; 1 Eq	EtOH; 40 %	Reflux	3 h	12 %	–
22	H	Ph	CH_2_	1:1	K_2_CO_3_; 1 Eq	H_2_O; 90 %	Reflux	3 h	54 %	40 %
23	H	Ph	CH_2_	1:1	K_2_CO_3_; 1 Eq	H_2_O; 40 %	MW 50 W	1 min	38 %*	
24	H	Ph	CH_2_	1:1	K_2_CO_3_; 1 Eq	–	MW 50 W; TBAB 0.1 Eq	5 min	56 %*	50 %
25	H	Ph	CH_2_	1:1	K_2_CO_3_; 1 Eq	EtOH; 50 %	MW 50 W; TBAB 0.1 Eq	5 min	43 %*	–
26	H	Ph	CH_2_	1:1	K_2_CO_3_; 1 Eq	H_2_O; 40 %	)))	40 min	64 %*	59 %
27	H	Ph	CH_2_	1:1	K_2_CO_3_; 1 Eq	EtOH; 40 %	)))	20 min	24 %*	–
28	H	Ph	CH_2_	1:1	K_2_CO_3_; 1 Eq	H_2_O; 40 %	)))	60 min	100 %**	74 %
29	H	2-napht	CH_2_	1:1	K_2_CO_3_; 1 Eq	H_2_O; 40 %	)))	60 min	100 %**	89 %
30	H	1-napht	CH_2_	1:1	K_2_CO_3_; 1 Eq	H_2_O; 40 %	)))	60 min	100 %**	81 %
31	Cl	2-napht	CH_2_	1:1	K_2_CO_3_; 1 Eq	H_2_O; 40 %	)))	60 min	100 %**	84 %

*LC-MS, **purity after isolation by LC-MS, MW – reaction in microwave reactor, ))) – ultrasound-assisted reaction.

As part of further research, an attempt was made to synthesize compound **10a** according to a new procedure: the reaction between 2-substituted analogs of 1,4-dihydroquinazoline (**5f–g**), or 1*H*-benzimidazoles (**5a–e**) with the corresponding arylsulfonamides (**6a–c**).

First, the reactions were carried out between 2-chloro-1*H*-benzimidazole (**5a**) and benzenesulfonamide (**6a**) in ethanol. However, after 20 h of heating under reflux, no traces of the product were observed. The MW and ultrasonic variants were also tested, using TEA as the base and DMF or EtOH as the solvent, but no product was found either. In the next step, 2–methylthiobenzimidazole (**5b**) was used instead of 2-chlorobenzimidazole (**5a**). The reactions were carried out with or without a basic agent (TEA, DBU, DIPEA, KOH, DMAP) in solvents such as pyridine, DMF, ACN, EtOH, or solvent-free. We tested the conventional, MW and ultrasonic variants. Unfortunately, none achieved a product with content exceeding 6 % in the post-reaction mixture. Similar results were obtained by using 2-ethylthiobenzimidazole (**5c**), or 2-(methanesulfonyl)-1*H*-benzimidazole (**5d**) as a substrate instead of 2-methylthiobenzimidazole (**5b**). Interestingly, a slightly higher product content was observed when 2-naphthalenesulfonamide (**6c**) was used instead of benzenesulfonamide (**6a**). The reaction with 2-(methanesulfonyl)-1*H*-benzimidazoles (**5d**) resulted in 14 % product content **10c**.

Much more favorable was the course of this reaction in the preparation of dihydroquinazoline derivatives (**10e–i**). The reaction between 2-(methylsulfanyl)-3,4-dihydroquinazoline (**5f**) and benzenesulfonamide (**6a**) in the presence of TEA made it possible to obtain **10e** with a 75 % yield of an isolated product without the need for a solvent, after 3 h. Importantly, the replacement of TEA with the more ecological potassium carbonate resulted in only a slight decrease in the yield of the isolated product compared to the previous variant (with a higher content in the post-reaction mixture). Conducting the reaction in the MW variant with a completely dry reaction mixture did not allow the reaction to proceed (no temperature rise in the reaction mixture). However, wetting the mixture with a small portion of the solvent (5 % by mass) allows the product to be obtained with a yield of over 60 % within 30 s. What is also important is that the reaction also takes place under ultrasonic conditions, with water as the solvent. The use of the developed MW method allowed to obtain 3 derivatives of **10f–h** at the yields of 69–85 % relative to the weight of the isolated product (Table 5).

**Table 5:** Development the method **C** of synthesis.


No.	Y	R	Ar	X	Ratio of **5:6**	Base	Solvent; mass [%]	Conditions	Time	Product content*	
										LC	Yield isol
1	–	H	Ph	Cl	1:1	–	EtOH; 60 %	200 °C; p	20 h	0 %	–
2	–	H	Ph	Cl	1:1	TEA; 1.5 Eq	DMF; 60 %	MW 100 W	5 min	0 %*	–
3	–	H	Ph	Cl	1:1	TEA; 3 Eq	EtOH; 60 %	MW 100 W	5 min	0 %	–
4	–	H	Ph	Cl	1:1	K_2_CO_3_; 3 Eq	DMF; 95 %	))); TBAB 0.1 Eq	10 min	0 %*	–
5	–	H	Ph	SCH_3_	1:1	TEA; 3 Eq	–	180 °C	2d	0 %*	–
6	–	H	Ph	SCH_3_	1:1	TEA; 1.5 Eq	–	MW 50 W	5 min	2 %	–
7	–	H	Ph	SCH_3_	1:1	TEA; 1.5 Eq	EtOH; 50 %	MW 50 W	5 min	0 %	–
8	–	H	Ph	SCH_3_	1:1	TEA; 1.5 Eq	DMF; 50 %	MW 50 W	5 min	0 %	–
9	–	H	Ph	SCH_3_	1:1	Pyridine; 1.5 Eq	–	MW 50 W	5 min	0 %	–
10	–	H	Ph	SCH_3_	1:1	DBU; 1.5 Eq	–	MW 50 W	1 min	5 %	–
11	–	H	Ph	SCH_3_	1:1	DBU; 1.5 Eq	DMF; 50 %	MW 50 W	10 min	2 %	–
12	–	H	Ph	SCH_3_	1:1	DBU; 3 Eq	DMF; 50 %	MW 50 W; TBAB 0.1 eq	40 min	6 %	–
13	–	H	Ph	SCH_3_	1:1	DBU; 1.5 Eq	MeCN; 50 %	MW 50 W; TBAB 0.1 eq	5 min	0 %	–
14	–	H	Ph	SCH_3_	1:1	DBU; 1.5 Eq	DMF; 50 %	)))	60 min	0 %	–
15	–	H	Ph	SCH_3_	1:1	DIPEA; 1.5 Eq	DMF; 50 %	MW 50 W	10 min	0 %	–
16	–	H	Ph	SCH_3_	1:1	DMAP; 1.5 Eq	DMF; 50 %	MW 50 W	10 min	3 %	–
17	–	H	Ph	SCH_3_	1:1	KOH; 1.5 Eq	DMF; 50 %	MW 50 W	5 min	0 %	–
18	–	H	Ph	SC_2_H_5_	1:1	TEA; 1 Eq	–	130 °C	2d	0 %	–
19	–	H	Ph	SC_2_H_5_	1:1	TEA; 1 Eq	–	230 °C	2d	0.8 %	–
20	–	H	Ph	SC_2_H_5_	1:1	DBU; 1.5 Eq	DMF; 50 %	MW 50 W; TBAB 0.1 Eq	10 min	0.5 %	–
21	–	H	Ph	SO_2_CH_3_	1:1	TEA; 1 Eq	EtOH; 65 %	Reflux	2d	2 %*	–
22	–	H	Ph	SO_2_CH_3_	1:1	TEA; 1 Eq		Reflux	2d	0 %*	–
23	–	Cl	Ph	Cl	1:1	K_2_CO_3_; 3 Eq	–	MW 100 W; TBAB 0.1 Eq	2 min	0 %*	–
24	–	H	2-napht	Cl	1:1	K_2_CO_3_; 3 Eq	–	MW 100 W; TBAB 0.1 Eq	2 min	0 %*	–
25	–	H	2-napht	SCH_3_	1:1	TEA; 2 Eq	–	MW 50 W	5 min	0 %	–
26	–	H	2-napht	SCH_3_	1:1	TEA; 2 Eq	EtOH; 60 %	MW 50 W	5 min	0 %	–
27	–	H	2-napht	SCH_3_	1:1	TEA; 3 Eq	DMF; 60 %	MW 50 W	5 min	0 %	–
28	–	H	2-napht	SC_2_H_5_	1:1	TEA; 3 Eq	EtOH; 85 %	Reflux	2d	0.4 %*	–
29	–	H	2-napht	SO_2_CH_3_	1:1	TEA; 1 Eq	–	200 °C	2d	14 %*	–
30	–	H	2-napht	SO_2_CH_3_	1:1	TEA; 3 Eq	–	MW 50 W	5 min	0 %*	–
31	–	H	2-napht	SO_2_CH_3_	1:1	–	EtOH; 65 %	MW 50 W	5 min	0 %	–
32	–	H	2-napht	SO_2_CH_3_	1:1	TEA; 2 Eq	–	MW 50 W	5 min	0 %	–
33	–	H	2-napht	SO_2_CH_3_	1:1	TEA; 2 Eq	EtOH; 60 %	MW 50 W	5 min	0 %	–
34	–	H	2-napht	SO_2_CH_3_	1:1	TEA; 2 Eq	DMF; 60 %	MW 50 W	5 min	0 %	–
35	CH_2_	H	Ph	SCH_3_	1:1	Et_3_N; 1 Eq	–	180 °C	3 h	79 %	75 %
36	CH_2_	H	Ph	SCH_3_	1:1	K_2_CO_3_; 1 Eq	–	180 °C	3 h	97 %*	66 %
37	CH_2_	H	Ph	SCH_3_	1:1	K_2_CO_3_; 1 Eq	H_2_O; 5 %	MW 50 W	30 s	73 %	63 %
38	CH_2_	H	Ph	SCH_3_	1:1	K_2_CO_3_; 1 Eq	H_2_O; 5 %	MW 50 W; TBAB 0.1 Eq	30 s	68 %* 100 %**	64 %
39	CH_2_	H	Ph	SCH_3_	1:1	K_2_CO_3_; 1 Eq	EtOH; 5 %	MW 50 W	30 s	12 %	–
40	CH_2_	H	Ph	SCH_3_	1:1	K_2_CO_3_; 1 Eq	DMF; 50 %	MW 50 W	30 s	90 %	55 %
41	CH_2_	H	Ph	SCH_3_	1:1	K_2_CO_3_; 1 Eq	EtOH; 90 %	)))	60 min	87 %*	–
42	CH_2_	H	Ph	SCH_3_	1:1	K_2_CO_3_; 1 Eq	H_2_O; 90 %	)))	60 min	90 %	78 %
43	CH_2_	H	Ph	SCH_3_	1:1	K_2_CO_3_; 1 Eq	DMF; 90 %	)))	60 min	0 %*	–
44	CH_2_	H	Ph	SCH_3_	1:1	DBU; 1.5 Eq	DMF; 50 %	)))	60 min	8 %	–
45	CH_2_	H	2-napht	SCH_3_	1:1	K_2_CO_3_; 1 Eq	H_2_O; 5 %	MW 50 W	30 s	100 %**	85 %
46	CH_2_	H	1-napht	SCH_3_	1:1	K_2_CO_3_; 1 Eq	H_2_O; 5 %	MW 50 W	30 s	100 %**	76 %
47	CH_2_	Cl	2-napht	SCH_3_	1:1	K_2_CO_3_; 1 Eq	H_2_O; 5 %	MW 50 W	30 s	100 %**	69 %

*LC-MS, **purity after isolation by LC-MS, MW – reaction in microwave reactor, ))) – ultrasound-assisted reaction.

**Fig. 3:**
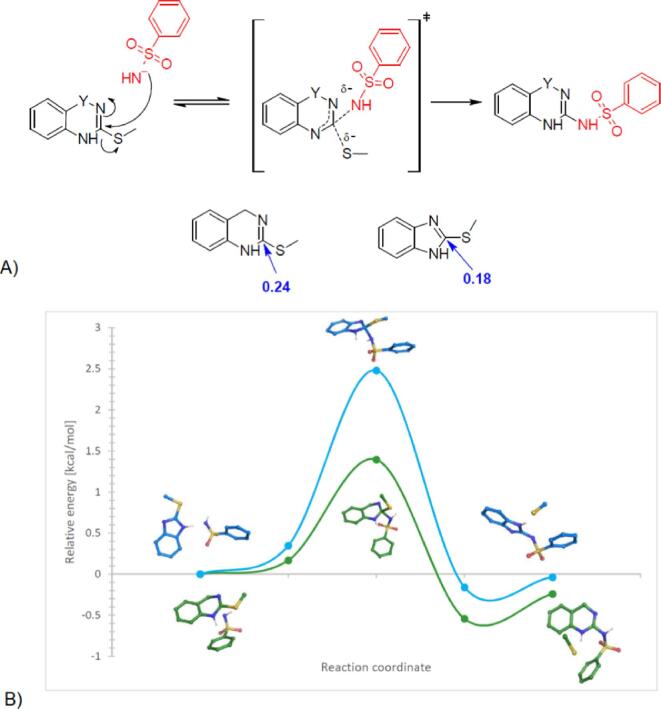
A) Proposed reaction mechanism, consistent with the commonly accepted mechanism of nucleophilic substitution reaction. The calculated Fukui indices (f-NN-LUMO) are marked in blue. B) Gibbs free energy changes for the nucleophilic substitution reaction of 2-(methylsulfanyl)-1,4-dihydroquinazoline (**5g**) (green marked), or 2-(methylsulfanyl)-*1H*-benzimidazole (**5a**) (blue marked) and the deprotonated benzenesulfonamide (**6a**). Values are given in kcal/mol relative to the energy of the substrates.

During the optimization of method **C**, the clear difference in reactivity between 2-(methylsulfanyl)-1,4-dihydroquinazoline (**5f**) and 2-(methylsulfanyl)-1*H*-benzimidazole (**5a**) was noticed. Both compounds have a similar structure and an identical leaving group. Due to huge differences in reactivity, we decided to investigate this phenomenon using molecular modeling methods. At the beginning, we proposed a reaction mechanism consistent with the generally accepted S_N_2 nucleophilic substitution mechanism (Fig. 3).

We optimized the structures of products and substrates, using Jaguar suite of *ab initio* quantum chemistry program [38] (M06-2X functional [39] and the 6-31G ** basis set [40]). Fukui indexes were also determined for the substrates [41]. For an electrophile, the atoms that are most susceptible to attack by a nucleophile are indicated by the high positive values of f_NN for the LUMO [41]. The calculated values indicate that the carbon atom attached to the methylsulfanyl group in **5f** is more susceptible to nucleophile attack than in **5a**, confirming the reactivity observed experimentally.

In the next step, an attempt was made to determine transition states in both of these reactions. To locate transition states, the potential energy surface was first explored approximately using the standard method, followed by a quadratic synchronous transit (QST) [42]. Intrinsic reaction coordinate (IRC) calculations were employed to locate reagent and product minima connected with the transition states for each considered reaction step (Table 6) [43].

**Table 6:** Free energies and bond length in the reaction center.

Components	**5f+6a**			**5a+6a**		
	Free Energy [kcal/mol]	C—S [Å]	C—N [Å]	Free Energy [kcal/mol]	C—S [Å]	C—N [Å]
Substrates	–32.639	1.79	–	–32.114	1.76	–
Initial complex	−31.471	1.83	2.13	−31.767	1.81	1.95
TS	−31.244	1.90	1.72	−29.634	1.84	1.72
Products complex	–33.182	2.67	1.44	–32.273	1.90	1.54
Products	–32.879	–	1.42	–32.151	–	1.41

The obtained results show that the course of the reaction between 2-(methylsulfanyl)-1,4-dihydroquinazoline (**5f**) (green marked) and the deprotonated benzenesulfonamide (**6a**) is characterized by a much more favorable energy profile than in the case of the reaction between 2-(methylsulfanyl)-1*H*-benzimidazole (**5a**) (blue marked) and **6a** (Fig. 3**B**). The computational analyses performed are consistent with the previously observed differences in reactivity. The high activation barrier for the reaction of **5a** and **6a** indicates a difficult course of the reaction.

The compounds for biological tests were selected on the basis of early pharmacokinetic parameters analyzes (ADMET) *in silico* [49] and checking possible bioactivity according to the *Similarity Ensemble Approach* (SEA) [50]. After the SEA analysis, compounds from the group of 1-naphthalenesulfonamide derivatives (**10f, 11b**) were selected for biological research. Activity related to the serotonin receptor 5-HT_6_ and PKA catalytic subunit alpha were predicted for them, which, according to literature reports, may be associated with the pathogenesis and treatment of CNS cancers [51], [52], [53]. Compound **10a** was also selected as the reference compound in the class of aryl sulfonamide cyclic arylguanidines on which the development of the synthesis method was based.

High absorbability from the digestive system and compliance with druglikeness determinants, such as Lipinski's rule, are predicted for all compounds [54]. The selected molecules did not show PAINS alerts [55]. Following the boiled-egg scheme [56], **10a**, **11b**, and **11d** are at the boundary of the area of ​​ability to cross the blood–brain barrier (BBB). Compound **10f** was classified as barrier-penetrating. However, the calculated CNS MPO parameters indicate that all of the compounds may exhibit high CNS drugability (Table 7) [57].

**Table 7:** Compounds selected for biological tests and it’s ADME properties (determined *in silico*).

Name	Structure	CNS MPO score	BBB penetration
10a		5.5	No
11b		5.0	No
11d		4.4	No
10f		4.6	Yes

Considering the above data, a group of selected compounds was tested for cytotoxic activity in two human cell lines, including cancer cell lines of brain origin, i.e., 1321 N1 astrocytoma, as well as breast adenocarcinoma line MDA-MB-231.

**Fig. 4:**
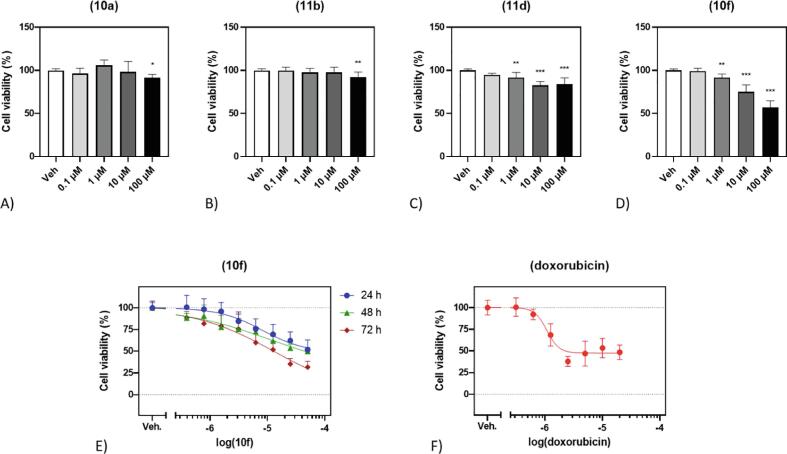
Effect of compounds **10a**, **11b**, **11d**, and **10f** on the viability on the 1321 N1 human astrocytoma cell line. The cells were exposed to either vehicle (0.1 % DMSO), or A) **10a**, B) **11b**, C) **11d**, or D) **10f**) for 24 h. Then, cell viability was assessed by the means of MTS assay. In E), the cytotoxic properties of the compound **10f** were studied in time- and dose-dependent manner. The 1321 N1 cells were subjected to the gradient of concentrations of **10f** for 24 h (●), 48 h (▲), or 72 h (♦). In the same cell line, F) doxorubicin inhibited cell viability with IC_50_ of 1.1 µM at 24 h time point. The IC_50_ were calculated as a dose that causes a 50 % decrease in cell viability relative to the maximum inhibition observed.

Compounds **10a**, **11b**, **11d**, and **10f** were subjected to MTS cytotoxicity tests against 1321 N1 cells. Two of the tested compounds (**10f** and **11d**) showed activity already at a concentration of 1 µM. The remaining compounds displayed relatively modest activity, with statistically significant suppression of cell viability detectable only at a concentration of 100 µM (Fig. 4**A–D**).

For **10f**, i.e., the most active compound, the calculated IC_50_ value was 8.22 µM at 24 h timepoint (Fig. 4**E**). At the same time point, doxorubicin suppressed 1321 N1 cell viability with IC_50_ of 1.1 µM (Fig. 4**F**). The viability of the cells exposed to **10f** for 24 h dropped to 48.0 % compared to vehicle control, while the cells treated with doxorubicin experienced decline to 47.5 %. After 72 h, the decrease in viability of **10f**-treated cells was even more pronounced as only 32 % of the cells exposed to 50 µM of **10f** were still viable. At the same time point, the compound yielded the IC_50_ of 0.13 µM (Fig. 4**E)**.

**Fig. 5:**
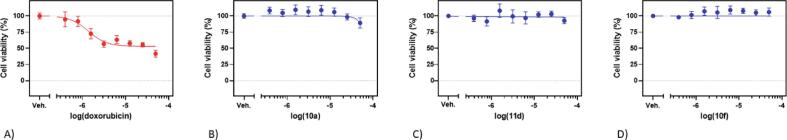
Effect of doxorubicin and compounds **10a**, **10d**, and **11f** on the viability of human MDA-MB-231 breast cancer cell line. The cells were exposed to either A) doxorubicin, B) **10a**, C) **10d**, or D) **11f** for 24 h. The cytotoxic effect of the compounds was evaluated by the means of MTS assay. Doxorubicin yielded IC_50_ value of 1.4 µM while three other assessed compounds were inactive under the assay conditions. The IC_50_ were calculated as a dose that causes a 50 % decrease in cell viability relative to the maximum inhibition observed.

The activity of compounds **10a**, **10f**, and **11d** against human breast adenocarcinoma line MDA-MB-231 were assessed in MTS tests. Interestingly, only compound **10a**, which had the lowest activity against the CNS tumor line, showed little activity on the breast tumor line. The remaining compounds were completely inactive in the tested concentration range (Fig. 5).

The performed cytotoxicity studies showed a significant antitumor activity of **11f** against 1321N1astrocytoma (GBM) cell line. Therefore, we decided to test some of the pharmacokinetic and toxicological properties of this compound *in vitro*.

The permeability of the most promising compound **10f** was tested in PAMPA. This test allows the determination of the compound’s passive diffusion through biological membranes. Tested compound **10f** had a good calculated permeability coefficient (Pe = 5.0 ± 1.5 × 10^-6^ cm/s) in comparison with the well permeable reference caffeine (Pe = 12.2 ± 0.9 × 10^-6^ cm/s) and according to the breakpoint for permeable compounds (Pe ≥ 1.5 × 10^−6^ cm/s) described in the literature [44].

The metabolic stability was determined in mouse liver microsomes (MLMs). The proposed main metabolic pathways are presented in Table 8. The UPLC analysis of the tested compound after 120 min of incubation with MLMs indicated that **10f** was metabolized in 97 %. This experiment indicated that the tested compound is unstable. We identified 5 possible metabolites. The results show that the most probable metabolic pathway is the hydroxylation at the phenyl ring and double hydroxylation. Importantly, a large proportion of **10f** (about 50 %) was dehydrogenated. The most likely product is *N*-(quinazolin-2-yl)naphthalene-1-sulfonamide, which remains unchanged at 17.5 % and it is also partially hydroxylated and double-hydroxylated. On the basis of the UPLC-MS analysis of the control sample (**10f** in TRIS-HCl pH 7.4 buffer solution without microsomes), it can be concluded that a slight dehydrogenation of **10f** (5 %) can also occur as a result of incubation in an inorganic buffer alone, or as a result of longer storage in DMSO. Due to the high content of the dehydrogenated derivative in the mixture of metabolites, it may be important to determine both the cytotoxic activity and pharmacokinetic parameters for *N*-(quinazolin-2-yl) naphthalene-1-sulfonamide itself.

**Table 8:** Metabolic stability and metabolic pathways of 10f after incubation with mouse liver microsomes (MLMs).

Substrate	Molecular mass (*m/z*)	% remaining	Molecular mass of the metabolite (m/z)	Metabolic pathway
10f	338.23	3%	354.25 (M1)	*hydroxylation*
			370.20 (M2)	*double hydroxylation*
			370.13 (M3)	*double hydroxylation*
			370.33 (M4)	*double hydroxylation*
			372.19 (M5)	*double hydroxylation and double bound reduction*
Dehydrogenated-10f	336.17	17.5%	352.19 (DM1)	*hydroxylation*
			368.20 (DM2)	*double hydroxylation*

**Fig. 6:**
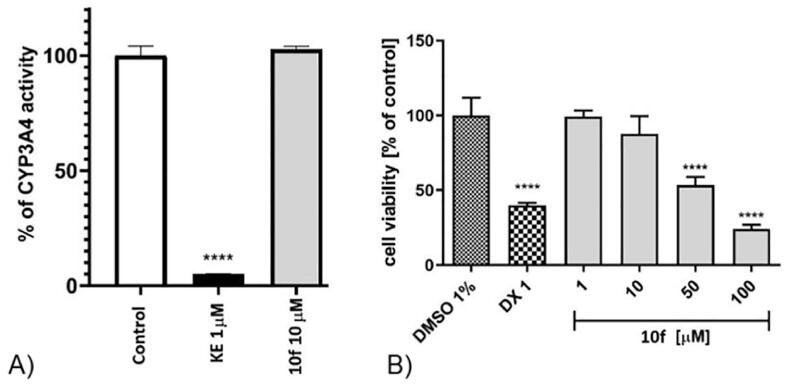
A) The influence on CYP3A4 activity. B) The effect of cytostatic drug doxorubicin (DX) and **10f** on hepatoma HepG2 cell line viability after 72 h of incubation at 37°, 5 % CO_2_. Statistical significance (****p < 0.0001) was analyzed by Graph Pad Prism 8.0.1 software using One-way ANOVA and Bonferroni’s Multiple Comparison Post Test. The compounds were examined in triplicate.

The potential risk of drug-drug interactions (DDI) was examined in luminescence-based CYP3A4 P450-Glo assay (Promega) (Fig. 6**A**). This CYP isoform was chosen for its leading role in the metabolism of xenibiotics [32]. We found that **10f** should not exhibit drug-drug interactions with CYP3A4.

The safety profile was estimated in the hepatotoxicity assay in hepatoma HepG2 cell line Fig. 6**B**. The results of cytotoxicity on HepG2 are similar to those obtained on astrocytoma 1321N1cells after 72 h. The observed cytotoxic effect is milder in HepG2 cells. Compound **10f** did not exhibit hepatotoxic properties in the range concentration of 1–10 µM. The statistically significant decrease in cells viability was observed at the concentration of 50, or 100 μM.

## 4 Conclusions

The conducted research show that method **A** (reaction of 2-aminobenzimidazole or 2-amino-3,4-dihydroquinazoline with arylsulfonyl chlorides) does not allow the selective preparation of compounds belonging to the arylsulfonamide derivatives of cyclic arylguanidines from a group of 2–substituted 3,4-dihydroquinazolines (**10e–h**) or 1*H*-benzimidazoles (**10a–d**) type. Method **B** allows obtaining the mentioned products. As part of this publication, we have developed an ultrasonic variant of method **B** (reaction of dimethyl-(arylsulfonyl)carbonodithioimidate with aryldiamines), which enables shortening the reaction time, using water as a solvent, and promoting the reactions of obtaining 1*H*-benzimidazoles derivatives, which are difficult to achieve under conventional conditions. In the sonochemical variant, we achieved the efficiency of 37–89 %, in 60 min (P = 80 W and f = 40 kHz), while in the microwave synthesis it was 38–74 %, in 0.5–4 min (P = 50 W). The development of a completely new **C** synthesis pathway allows highly selective preparation of 2-substituted 3,4-dihydroquinazolines in the solvent-free conditions. In the sonochemical variant, the efficiency reached 90 % in 60 min, when the solvent was water (P = 80 W and *f* = 40 kHz), while in the microwave synthesis it was 63–85 % in 0.5–4 min (P = 50 W). This method is characterized by improved atomic economics compared to method **B**. Moreover, the use of the microwave variant significantly shortens the reaction time, making the method more ecological. Importantly, the ultrasonic variant also proved successful in this path, allowing the reaction to be carried out in water as a solvent with high efficiency. In path **B**,

The conducted preliminary biological studies confirm the strong cytotoxic effect of the **10f** derivative (*N*-(1,4-dihydroquinazolin-2-yl)naphthalene-1-sulfonamide) against astrocytoma (GBM) line 1321 N1. The calculated IC_50_ value was 8.22 µM at 24 h timepoint (doxorubicin suppressed 1321 N1 cell viability with IC_50_ of 1.1 µM). The viability of the cells exposed to **10f** for 24 h dropped to 48.0 % compared to vehicle control, while the cells treated with doxorubicin experienced decline to 47.5 %. ADMET studies also confirm the possibility of penetrating the blood–brain barrier and the safety of its potential use in terms of DDI and hepatotoxicity.

## CRediT authorship contribution statement

**Przemysław Zaręba:** Conceptualization, Data curation, Funding acquisition, Investigation, Methodology, Project administration, Resources, Supervision, Validation, Visualization, Writing – original draft, Writing – review & editing. **Anna K. Drabczyk:** Investigation, Methodology, Writing – original draft. **Artur Wnorowski:** Investigation, Methodology, Resources, Writing – original draft, Visualization. **Edyta Pindelska:** Investigation, Visualization. **Gniewomir Latacz:** Investigation, Methodology, Resources, Writing – original draft, Visualization. **Jolanta Jaśkowska:** Investigation, Writing – original draft.

## Declaration of Competing Interest

The authors declare the following financial interests/personal relationships which may be considered as potential competing interests: Przemyslaw Zareba reports financial support was provided by National Science Centre Poland. Przemyslaw Zareba reports equipment, drugs, or supplies was provided by AGH University of Science and Technology Academic Computer Centre CYFRONET.

## Data Availability

No data was used for the research described in the article.
