# Supplementary material for: Eco-friendly methods of synthesis and preliminary biological evaluation of sulfonamide derivatives of cyclic arylguanidines
Source: Ultrason Sonochem. 2022 Sep 14;90:106165. doi: 10.1016/j.ultsonch.2022.106165 (PMC9529985; doi:10.1016/j.ultsonch.2022.106165)
Supplement: Supplementary Data 1 [file mmc1.pdf]

Table of Contents

1. **Detailed description of the synthesis methods** **1**
2. **Products characterization** **3**
   - 2.1. ***N*-(1H-benzimidazol-2-yl)benzenesulfonamide 10a:** **3**
   - 2.2. ***N*-(1H-benzimidazol-2-yl)naphthalene-1-sulfonamide 10b:** **5**
   - 2.3. ***N*-(1H-benzimidazol-2-yl)naphthalene-2-sulfonamide 10c:** **5**
   - 2.4. **1-(benzenesulfonyl)-1H-benzimidazol-2-amine 11a:** **6**
   - 2.5. **1-(naphthalene-1-sulfonyl)-1H-benzimidazol-2-amine 11b** **10**
   - 2.6. **1-(naphthalene-2-sulfonyl)-1H-benzimidazol-2-amine 11c** **12**
   - 2.7. **5-chloro-1-(naphthalene-1-sulfonyl)-1H-benzimidazol-2-amine 11d** **14**
   - 2.8. ***N*-(1,4-dihydroquinazolin-2-yl)benzenesulfonamide 10e** **16**
   - 2.9. ***N*-(1,4-dihydroquinazolin-2-yl)naphthalene-1-sulfonamide 10f** **18**
   - 2.10. ***N*-(1,4-dihydroquinazolin-2-yl)naphthalene-2-sulfonamide 10g** **20**
   - 2.11. ***N*-(6-chloro-1,4-dihydroquinazolin-2-yl)naphthalene-2-sulfonamide 10h** **22**
   - 2.12. **1-(benzenesulfonyl)-1,4-dihydroquinazolin-2-amine 11e** **24**
   - 2.13. **1-(naphthalene-1-sulfonyl)-1,4-dihydroquinazolin-2-amine 11h** **24**
3. **ADME-tox 10f** **25**
   - 3.1. **Permeability – PAMPA** **25**
   - 3.2. **Metabolic stability** **26**
4. **Development of synthesis methods (tables 3-5) - chromatographic analyzes** **28**
   - 4.1. **Development the method A of synthesis (Table 3)** **28**
   - 4.2. **Development the method B of synthesis (Table 4)** **35**
   - 4.3. **Development the method C of synthesis (Table 5)** **52**

1. Detailed description of the synthesis methods

Method A (synthesis according to Bocanegra-Garcia et al., 2012)

A mixture of 0.001 mol of aryl sulfochloride **2a–c**, 0.001 mol of amine **1a–c** and 0.0001 mol of DMAP was placed in a round bottom flask. The mixture was dissolved in methylene chloride (70% mass; 1.6–1.8 ml), then 0.003 mol of TEA was added dropwise while stirring. The reactions were carried out for 2 to 4 days, monitoring their progress on TLC. After this time, a sample was taken for analysis. Afterwards, the compound obtained was filtered off and dried.

Method A (synthesis according to Paget et al., 1969)

In a round bottom flask, 0.001 mol of amine **1a** in pyridine (72% mass; 0.8-1.0 ml) was placed, then 0.001 mol of aryl sulfochloride **2a–c** was added. The reactions were carried out for 7 days with continuous stirring at 50 °C, monitoring their progress on TLC. After this time, a sample was taken for analysis. Then, 5 ml of H_2O was added to the mixture and the resulting precipitate was filtered off. The precipitate was taken up in 10 ml of 4M NaOH and filtered off.

Method A (synthesis at room temperature)

In a round bottom flask, a mixture of 0.001 mol of aryl sulfochloride **2a** and 0.001 mol of amine **1a** was placed. The mixture was dissolved in DMF (90% mass; 3 ml). The reaction was carried out for 1 day, monitoring their progress on TLC. After this time, a sample was taken for analysis. Then, 5 ml of H_2O was added to the mixture and the resulting precipitate was filtered off.

Method A (microwave synthesis)

A mixture of 0.001 mol of aryl sulfochloride **2a**, 0.001 mol of amine **1a** was prepared in a round bottom flask. The mixture was dissolved in a suitable solvent (78–80% mass; 1.2-1.7 ml). The reactions were carried out for 30 s while monitoring their progress on TLC. After this time, a sample was taken for analysis.

Method B (synthesis according to Gompper and Hägele 1966)

A flask was filled with 0.001 mol of diamine **3a**, 0.001 mol of dimethyl(arylsulfonyl) dithioimidocarbonate **4a** and placed in 1 ml of DMF. The mixture was heated for 24 hours. After this time, 5 ml of water was added and filtered off.

Method B (synthesis according to Zali-Boeini et al., 2015)

To a warm (40 °C) mixture 0.001 mol of 1,2-diaminobenzene **3a**, K_2CO_3 1.5 Eq and hexadecyltrimethylammonium bromide HTAB, 0.1 Eq in H_2O/ EtOH (3:1, 4 mL), dimethyl (arylsulfonyl) dithioimidocarbonate (**4a**) 0.0011 mmol was added under stirring and then heated to reflux for 60 minutes. After cooling to room temperature, the reaction mixture was poured onto water (5 mL) and the crude product was filtered off.

Method B (microwave method)

A mixture of 0.001 mol diamine **3a–b**, 0.001/0.0012 mol dimethyl(arylsulfonyl)dithioimidocarbonate (**4a**) 1/1.5/3 Eq of the appropriate basic agent and 30–60% mass of the solvent was placed in a round bottom flask. For solvent-free reactions, the mixture was triturated in a mortar and transferred to a round bottom flask, then whipped with a stirring rod. The 0.1 Eq TBAB was also added in some variants. The flask was placed in the MW reactor and the reaction was carried out for 1–5 minutes. Due to the possibility of **3a–b** decomposition, care was taken that the reaction mixture did not get hotter than 150 °C. After this time, a sample was taken for analysis. Then, 5 ml of water was added to the reaction mixture and the resulting product was filtered off.

Method B (method in reflux)

A round bottom flask was charged with 0.001 mol diamine (**3a–d**), 0.001 mol dimethyl(arylsulfonyl) dithioimidocarbonate (**4a–c**), 1 Eq of the appropriate basic agent, and 40–95% mass of solvent. The reaction flask was heated for 2–8 hours. After this time, a sample was taken for analysis. Then, 5 ml of water was added to the reaction mixture and the resulting product was filtered off.

Method B (ultrasonic method)

A mixture of 0.001 mol diamine (**3a–d**), 0.001 mol dimethyl (arylsulfonyl)dithioimidocarbonate (**4a–c**), 1 Eq of K_2CO_3 and 40% mass of solvent were placed in a round bottom flask. Also, 0.1 Eq of TBAB was added in some variants. The flask with the reaction mixture was placed in an ultrasonic bath and the reaction was carried out for 20–60 minutes. After this time, a sample was taken for analysis and the resulting product was filtered off.

Method C (classical method)

A mixture of 0.001 mol of the **5a–d**, **5f** (alkylating agent), 0.001 mol of arylsulfonamide **6a**, **6c**, 1/3 Eq of the appropriate base and in some cases 60–85% mass of ethanol was placed in a round bottom flask. The reaction mixtures were heated (in the case of the solvent-free variant without stirring) in an oil bath for 3/20/48 hours at the temperature of 130/180/200/230 °C, after which

samples were taken for analysis. After cooling, water was added to the reaction mixture and stirred for 30 minutes. Then, the resulting precipitate was filtered off.

Method C (microwave method)

A mixture of 0.001 mol of the **5a/f** (alkylating agent), 0.001 mol of arylsulfonamide **6a**, 1–3 Eq of the appropriate base and 5–65% by weight of the solvent (or solvent-free variant) was placed in a round bottom flask. In some cases, 0.1 Eq of TBAB was also added. The reactions were carried out in a MW reactor for 0.5–40 minutes, after which a sample for testing was taken. After cooling, water was added to the reaction mixture and stirred for 30 minutes. Then, the resulting precipitate was filtered off.

Method C (ultrasonic method)

A mixture of 0.001 mol of the **5a–g** (alkylating agent), 0.001 mol of **6a–c** arylsulfonamide, 1/1.5 Eq of the appropriate basic agent and 50–90% by weight of the solvent was placed in a round bottom flask. In some cases, 0.1 Eq TBAB was also added. The flask with the reaction mixture was placed in an ultrasonic bath and the reaction was carried out for 20–60 minutes. After this time, a sample was taken for analysis and the resulting product was filtered off.

2. Products characterization

2.1. *N*-(1H-benzimidazol-2-yl)benzenesulfonamide 10a:

UPLC-MS:

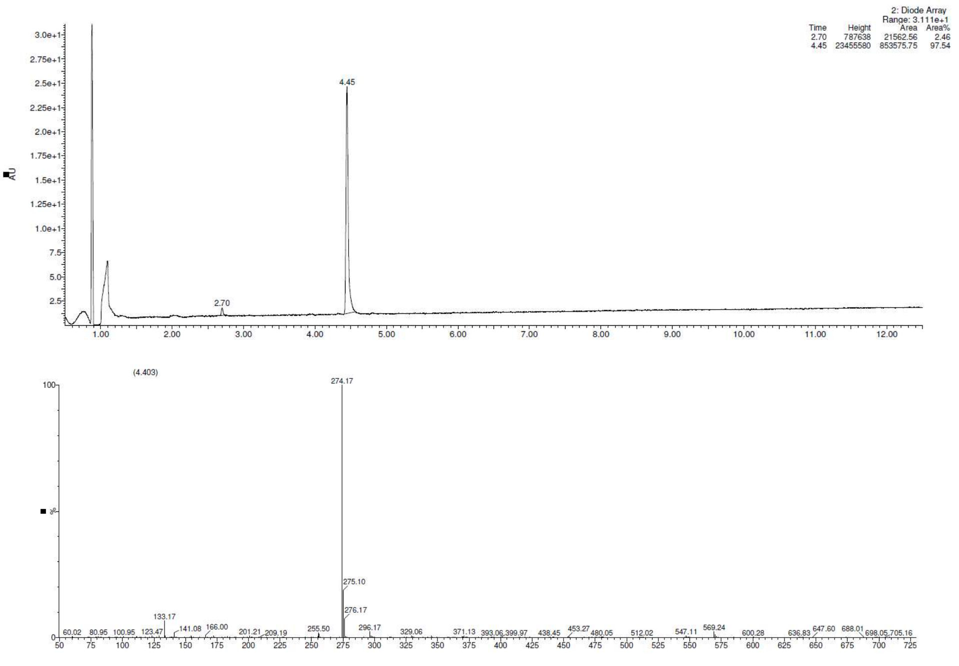

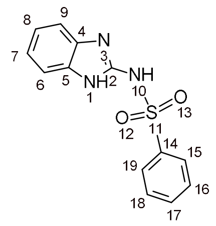

^{1}H NMR (400 MHz, DMSO) δ 11.93 (s, 2H $N^{1}H$,$N^{10}H$), 7.89 (dd, *J* = 7.7, 1.8 Hz, 2H, $C^{1}8H$, $C^{19}H$), 7.55 – 7.48 (m, 3H, $C^{9}H$, $C^{10}H$, $C^{16}H$), 7.30 – 7.25 (m, 2H, $C^{16}H$,$C^{18}H$), 7.14 – 7.09 (m, 2H, $C^{7}H$,$C^{8}H$).

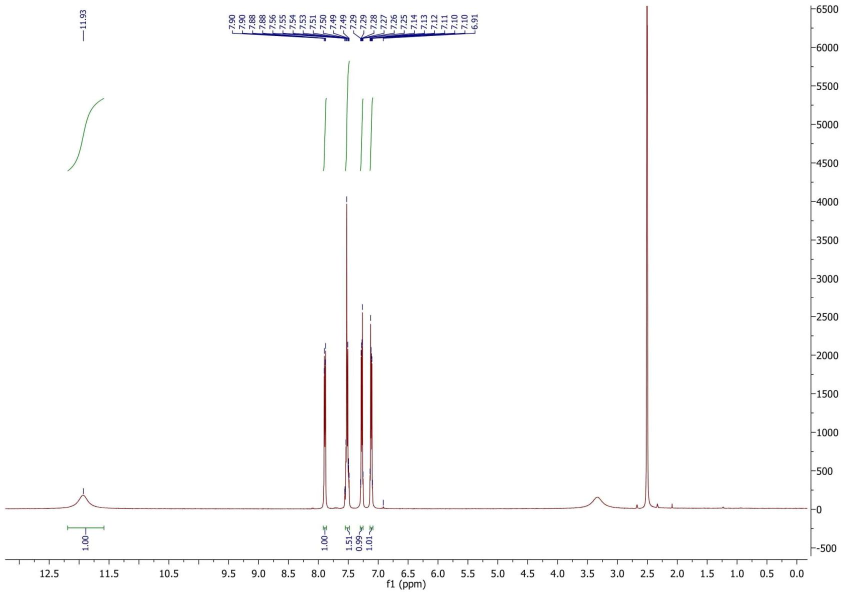

^{13}C NMR (101 MHz, DMSO) δ 150.08 C^{2}, 144.14 C^{4},C^{5}, 131.37 C^{14}, 129.67 C^{16} C^{18}, 128.80 C^{7},C^{8}, 125.51 C^{17}, 122.30 C^{15},C^{19}, 110.92 C^{6},C^{9}.

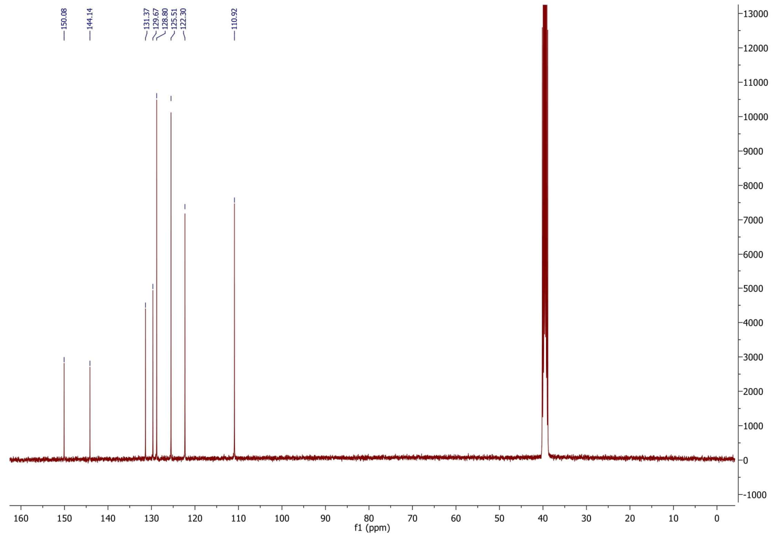

FT-IR:

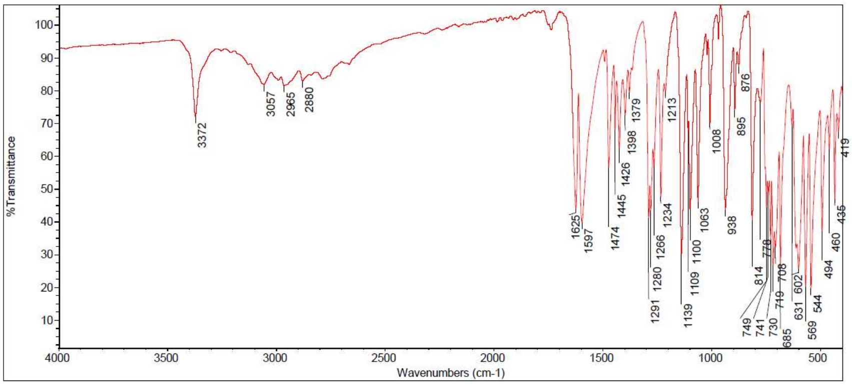

2.2. ***N*****-(1H-benzimidazol-2-yl)naphthalene-1-sulfonamide 10b:**

MS:

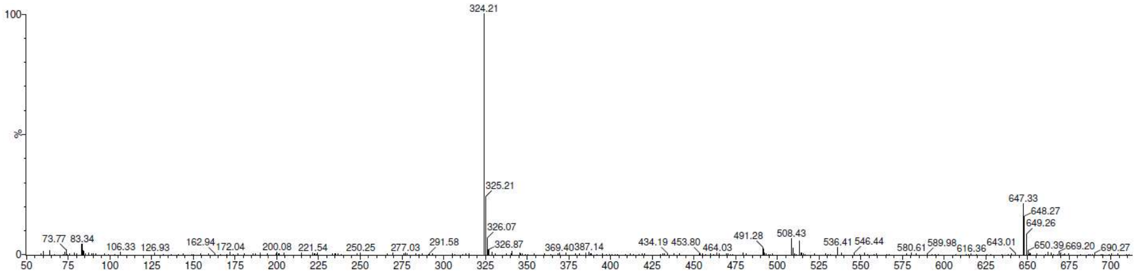

2.3. ***N*****-(1H-benzimidazol-2-yl)naphthalene-2-sulfonamide 10c:**

MS:

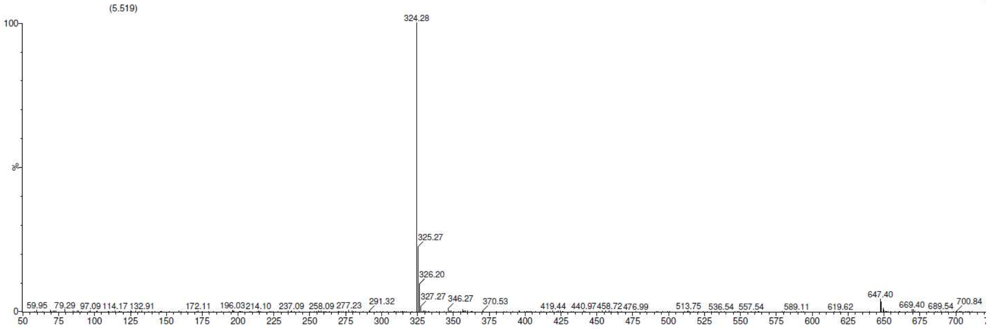

2.4. 1-(benzenesulfonyl)-1H-benzimidazol-2-amine 11a:

UPLC-MS:

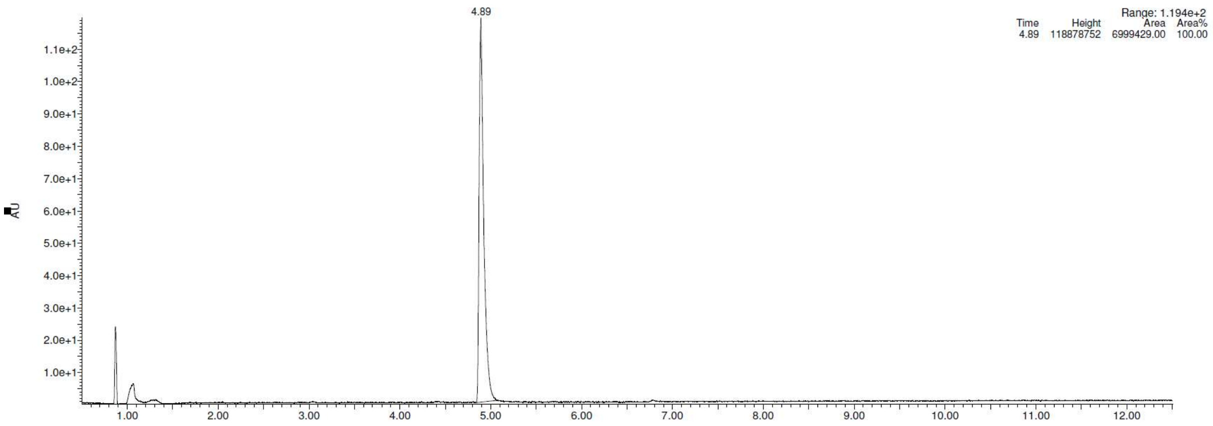

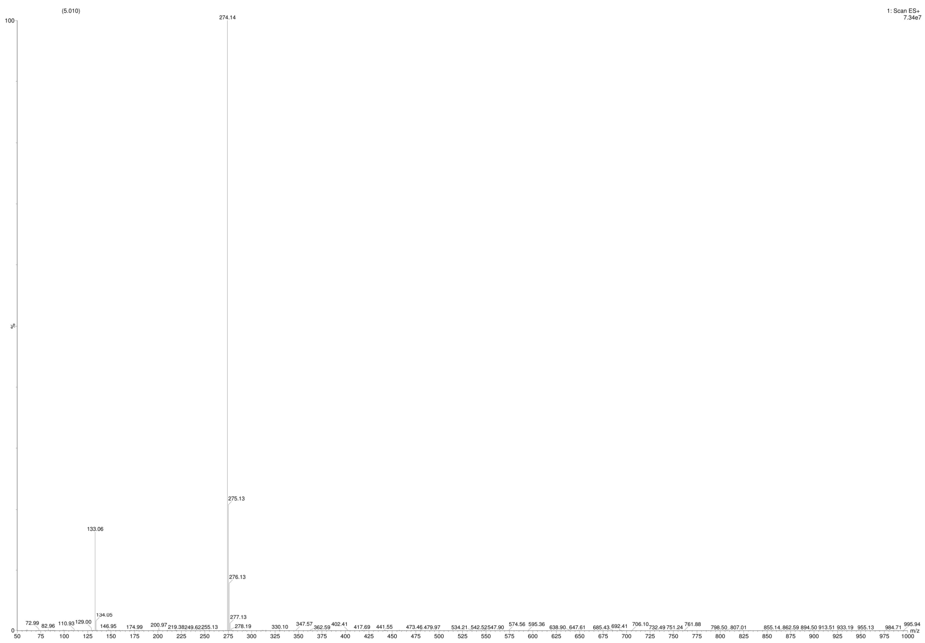

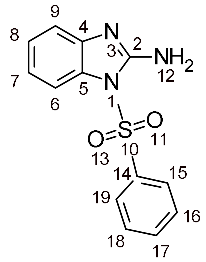

^{1}H NMR (500 MHz, DMSO) δ 8.02 (dt, J = 8.7, 1.6 Hz, 2H, $C^{19}H$, $C^{15}H$), 7.75 – 7.69 (m, 1H, $C^{17}H$), 7.66 (d, J = 7.9 Hz, 1H, $C^{6}H$), 7.63 – 7.58 (m, 2H, $C^{16}H$,$C^{18}H$), 7.18 (s, 2H, $N^{2}H$), 7.12 (dtd, J = 8.9, 7.8, 1.1 Hz, 2H, $C^{7}H$, $C^{9}H$), 7.04 – 6.98 (m, 1H, $C^{7}H$).

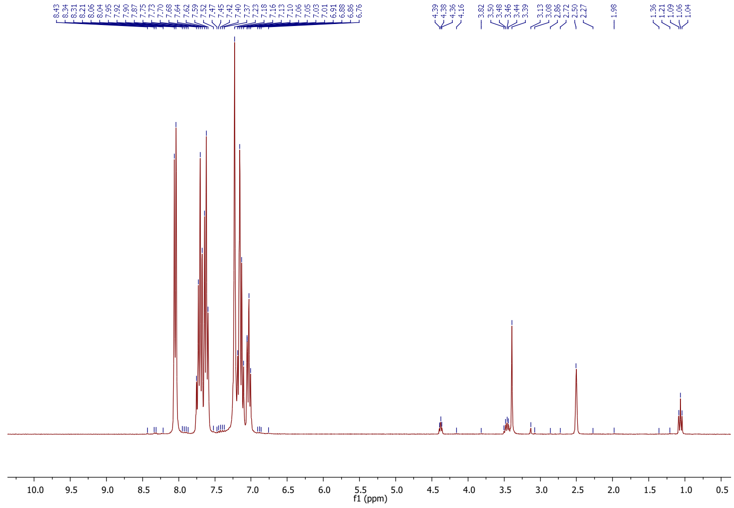

^{13}C NMR (126 MHz, DMSO) δ 152.51 C^{2}, 143.17 C^{4}, 137.07 C^{14}, 135.71 C^{17}, 130.52 C^{5}, 130.44 C^{16},C^{18}, 127.17 C^{15},C^{19}, 125.19 C^{8}, 121.06 C^{7}, 116.45 C^{9}, 112.60 C^{6}.

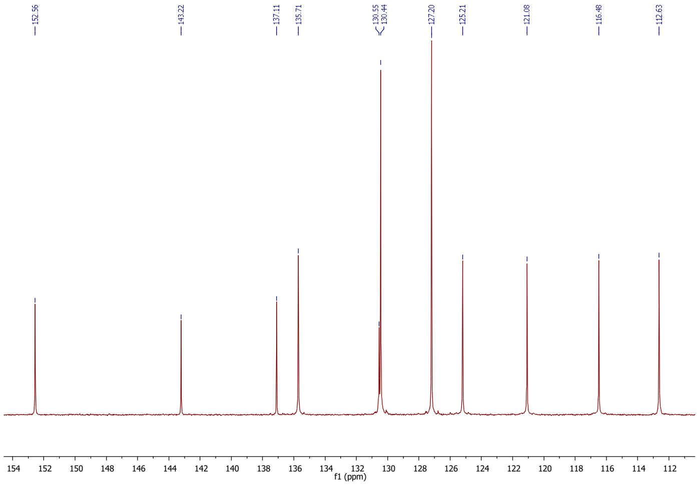

HSQC (DMSO):

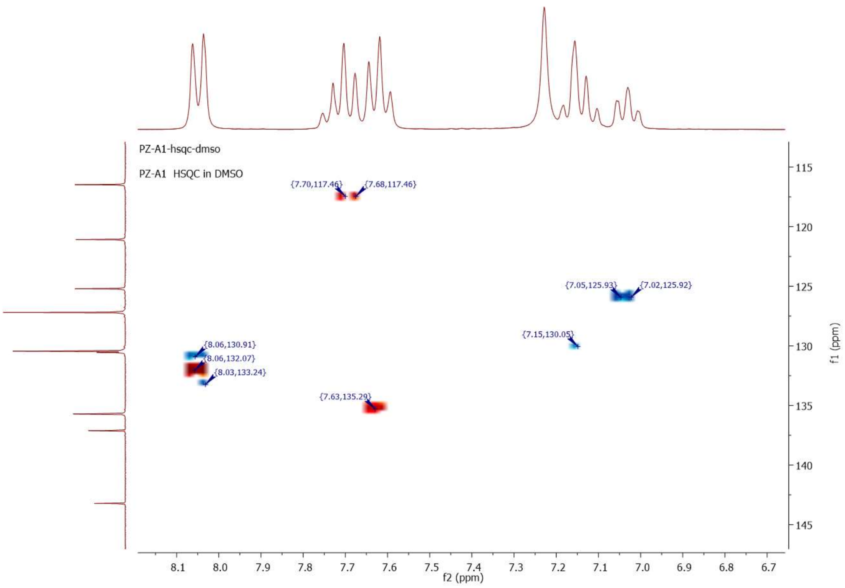

COSY (DMSO):

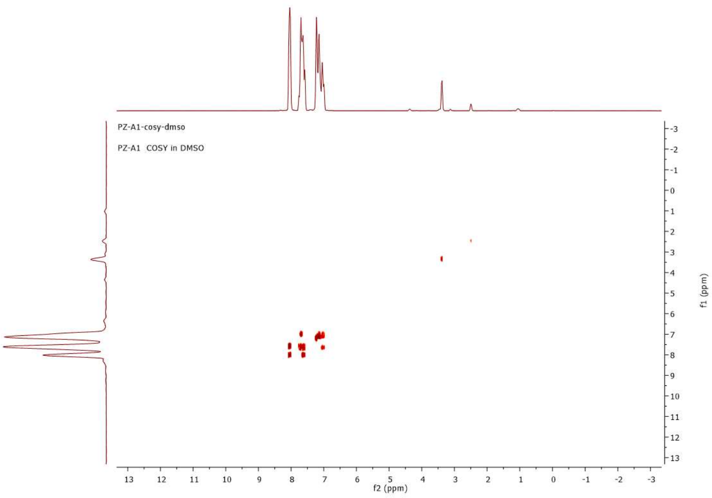

Comparison of the experimental ^{1}H NMR spectrum of sample **11a** with the calculated shift values using DFT methods for compound **10a**:

**10a**	B3lyp/6-311+G** [ppm]	EXP [ppm]
C4	142,71725	143,17
C9	119,64377	116,45
C8	122,20535	125,19
C7	121,99349	121,06
C6	108,29963	112,60
C14	133,12577	137,07
C2	144,19064	152,51
C15	126,57737	127,17
C15	129,37007	130,52
C17	133,2317	135,71
C16,C17	128,77301	130,44
C19	127,02998	127,17

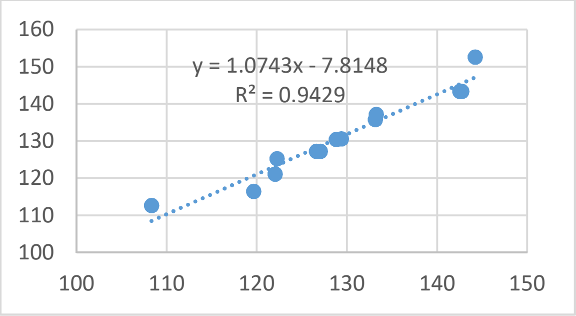

Comparison of the experimental ^{1}H NMR spectrum of sample **11a** with the calculated shift values using DFT methods for compound **11a**:

**11a**	B3lyp/6-311+G** [ppm]	EXP [ppm]
C2	149,1886	152,51
C4	143,2084	143,17
C9	117,8622	116,45
C8	124,7092	125,19
C7	120,366	121,06
C6	111,5642	112,60
C14	131,065	137,07
C15	126,8663	127,17
C15	129,0138	130,52
C17	133,6265	135,71
C16,C17	128,5612	130,44
C19	126,9818	127,17

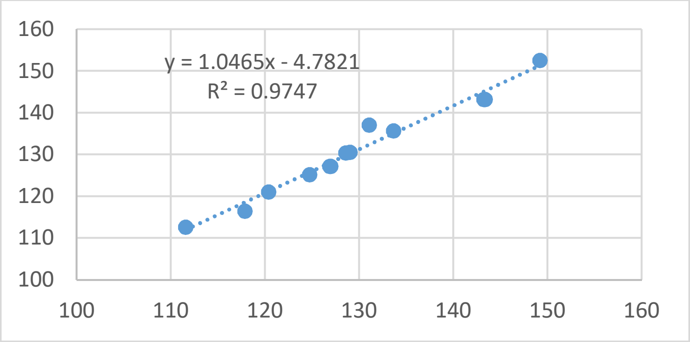

FT IR:

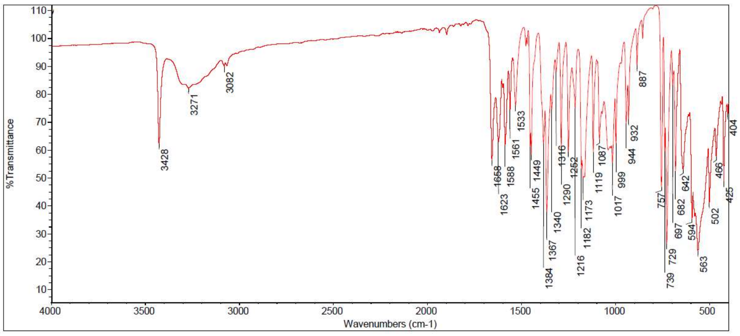

2.5. 1-(naphthalene-1-sulfonyl)-1H-benzimidazol-2-amine 11b

UPLC-MS:

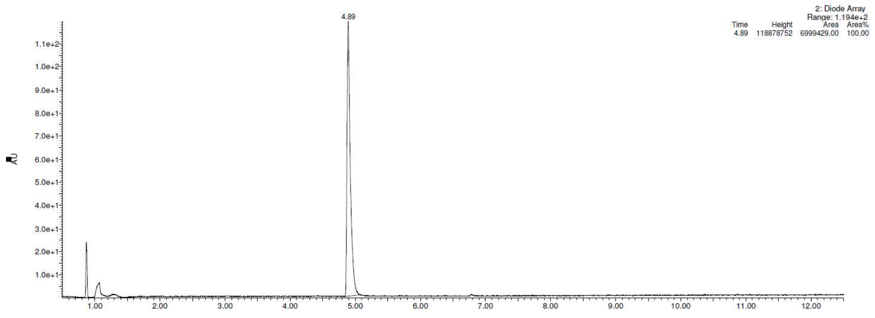

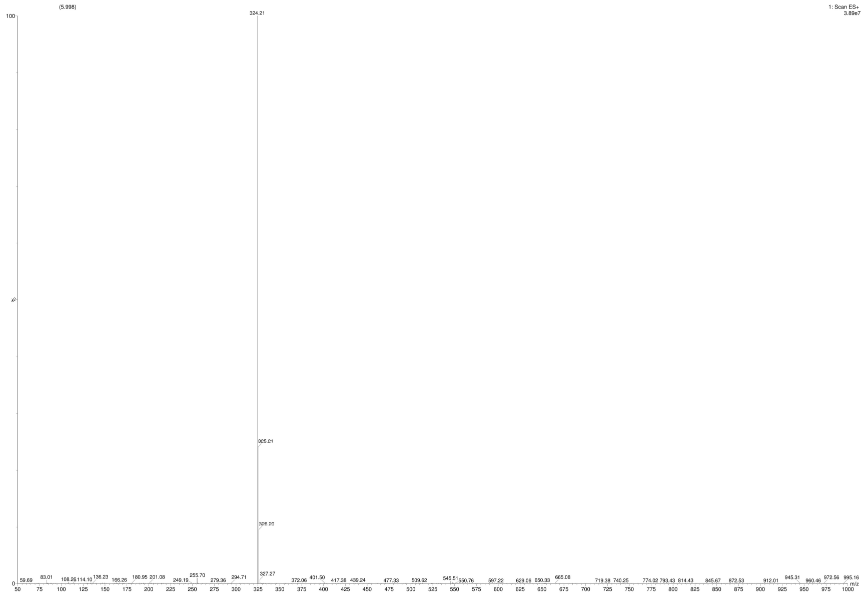

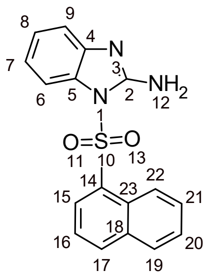

^{1}H NMR (400 MHz, DMSO) δ 8.56 – 8.50 (m, 1H $C^{21}H$), 8.46 (dd, *J* = 7.5, 1.1 Hz, 1H $C^{22}H$), 8.38 (d, *J* = 8.3 Hz, 1H $C^{17}H$), 8.12 (dd, *J* = 7.1, 2.3 Hz, 1H $C^{19}H$), 7.79 – 7.73 (m, 1H $C^{15}H$), 7.72 – 7.64 (m, 2H $C^{16}H$, $C^{20}H$), 7.41 (d, *J* = 7.8 Hz, 1H $C^{9}H$), 7.26 (s, 2H $N^{12}H$), 7.15 (d, *J* = 7.3 Hz, 1H $C^{6}H$), 7.07 (td, *J* = 7.7, 1.1 Hz, 1H $C^{8}H$), 6.96 – 6.90 (m, 1H $C^{7}H$).

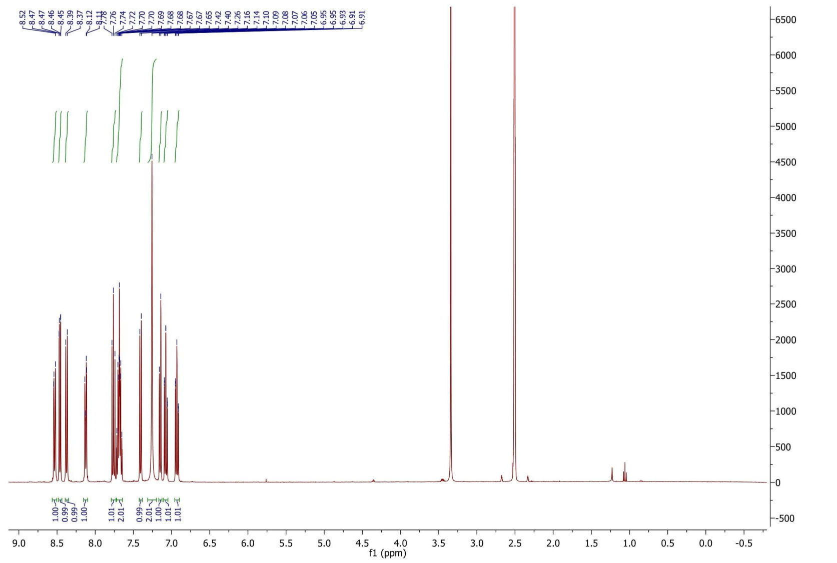

^{13}C NMR (101 MHz, DMSO) δ 152.60 C^{2}, 142.77 C^{4}, 137.04 C^{5}, 134.27 C^{14}, 132.17 C^{18}, 131.69 C^{15}, 130.52 C^{23}, 130.12 C^{7}, 129.67 C^{8}, 128.04 C^{19}, 127.56 C^{17}, 125.07 C^{21}, 124.91 C^{20}, 123.19 C^{16}, 120.83 C^{22}, 116.56 C^{9}, 112.22 C^{6}.

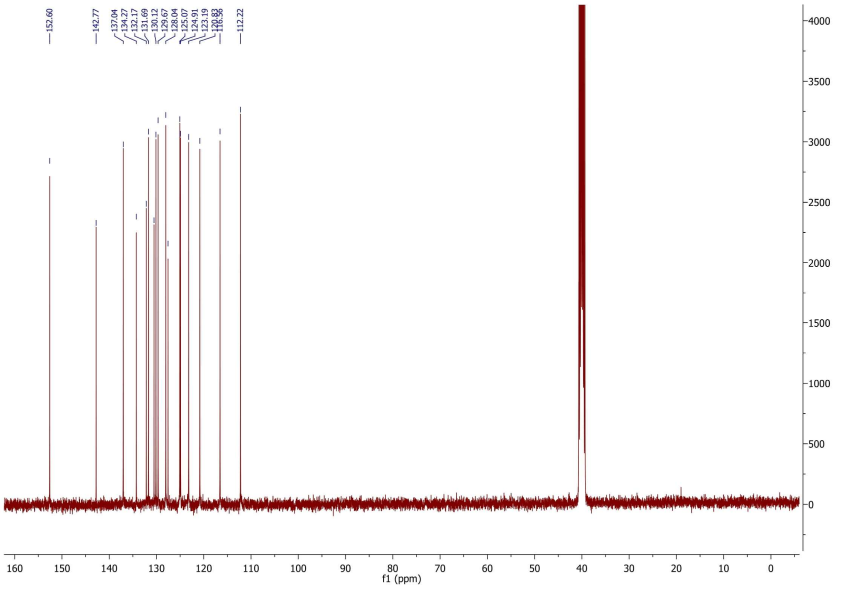

FT IR:

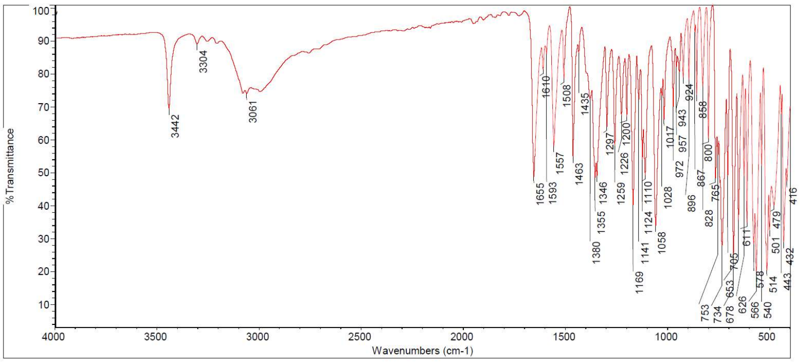

2.6. 1-(naphthalene-2-sulfonyl)-1H-benzimidazol-2-amine 11c

UPLC-MS:

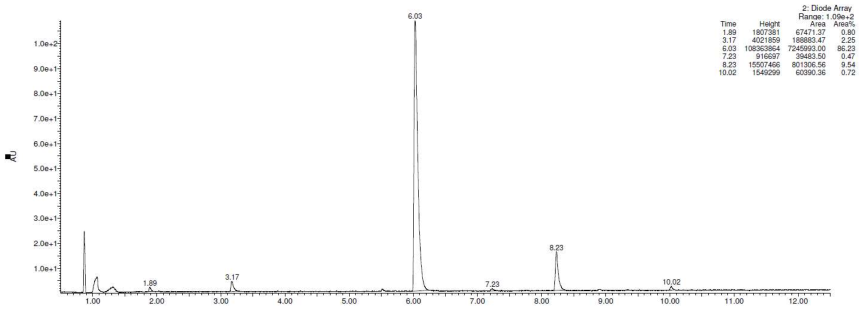

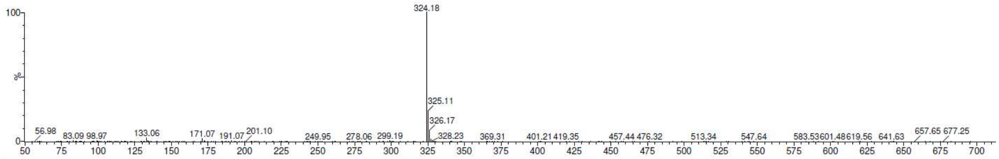

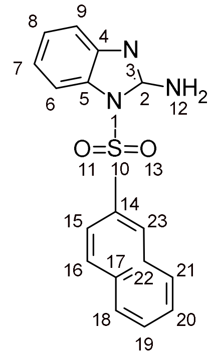

^{1}H NMR (400 MHz, DMSO) δ 8.90 (d, *J* = 1.8 Hz, 1H C^{23}H), 8.22 (d, *J* = 8.0 Hz, 1H, C^{21}H), 8.15 (d, *J* = 8.8 Hz, 1H, C^{16}H), 8.03 (d, *J* = 8.0 Hz, 1H C^{15}H), 7.90 (dd, *J* = 8.8, 2.0 Hz, 1H C^{18}H), 7.78 – 7.69 (m, 3H C^{9}H, C^{19}H,C^{20}H), 7.23 (s, 2H N^{12}H), 7.16 – 7.08 (m, 2H C^{8}H,C^{6}H), 7.07 – 6.98 (m, 1H C^{7}H).

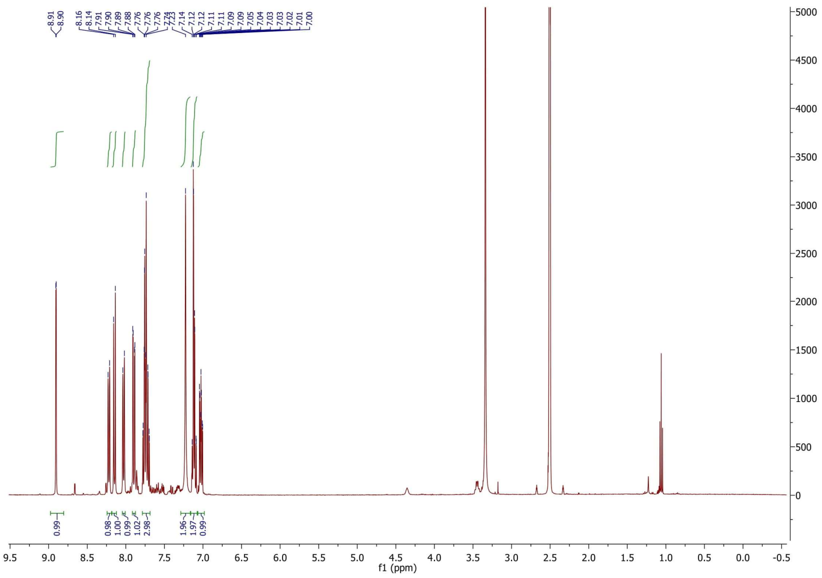

^{13}C NMR (101 MHz, DMSO) δ 152.52 C^{2}, 143.18 C^{4}, 135.59 C^{5}, 133.92 C^{14}, 131.81 C^{22}, 130.86 $C^{17,}$ 130.59 C^{16}, 130.56 C^{7}, 130.13 C^{8}, 129.58 C^{21}, 128.76 C^{18}, 128.45 C^{15}, 125.15 C^{20}, 121.31 C^{19}, 121.04 C^{23}, 116.43 C^{9}, 112.61 C^{6}.

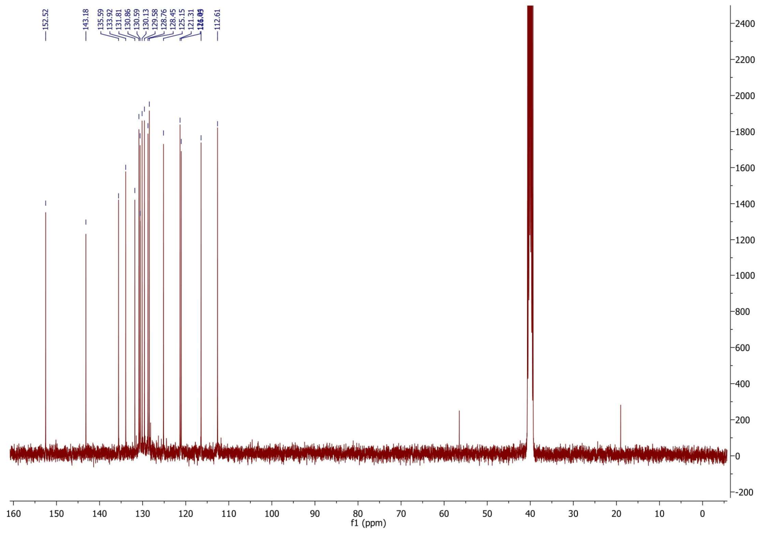

FT IR:

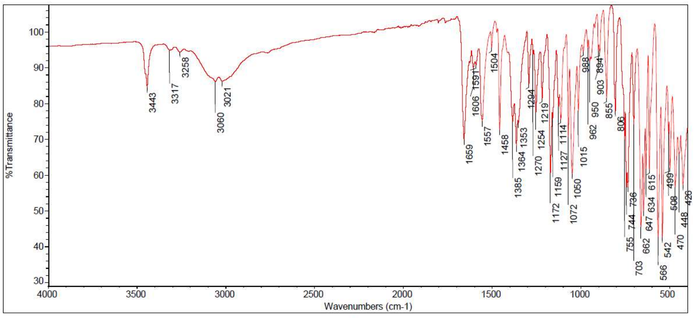

2.7. 5-chloro-1-(naphthalene-1-sulfonyl)-1H-benzimidazol-2-amine 11d

UPLC-MS:

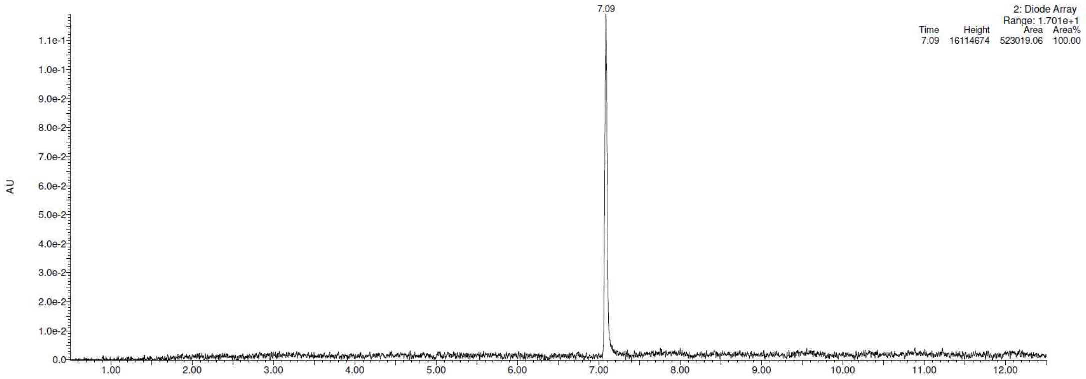

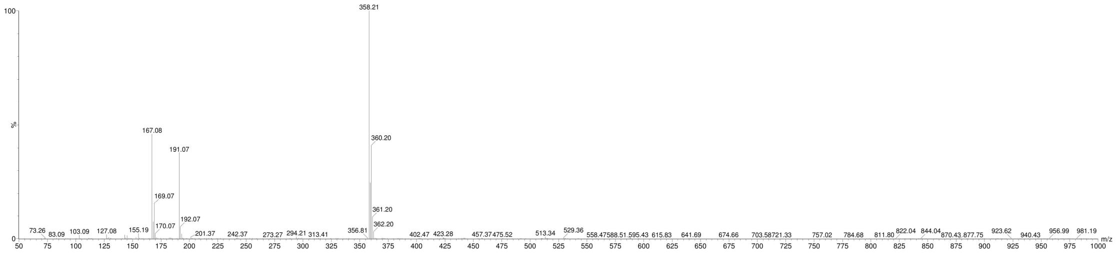

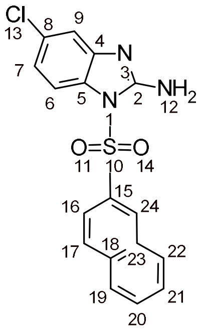

^{1}H NMR (400 MHz, DMSO) δ 8.55 – 8.37 (m, 3H $C^{17}H$,$C^{22}H$,$C^{24}H$), 8.18 – 8.11 (m, 1H, $C^{16}H$), 7.82 – 7.66 (m, 3H $C^{19}H$, $C^{21}H$,$C^{20}H$), 7.45 – 7.36 (m, 2H, $N^{12}H$), 7.28 – 7.11 (m, 2H, $C^{9}H$,$C^{6}H$), 7.10 – 6.93 (m, 1H $C^{7}H$).

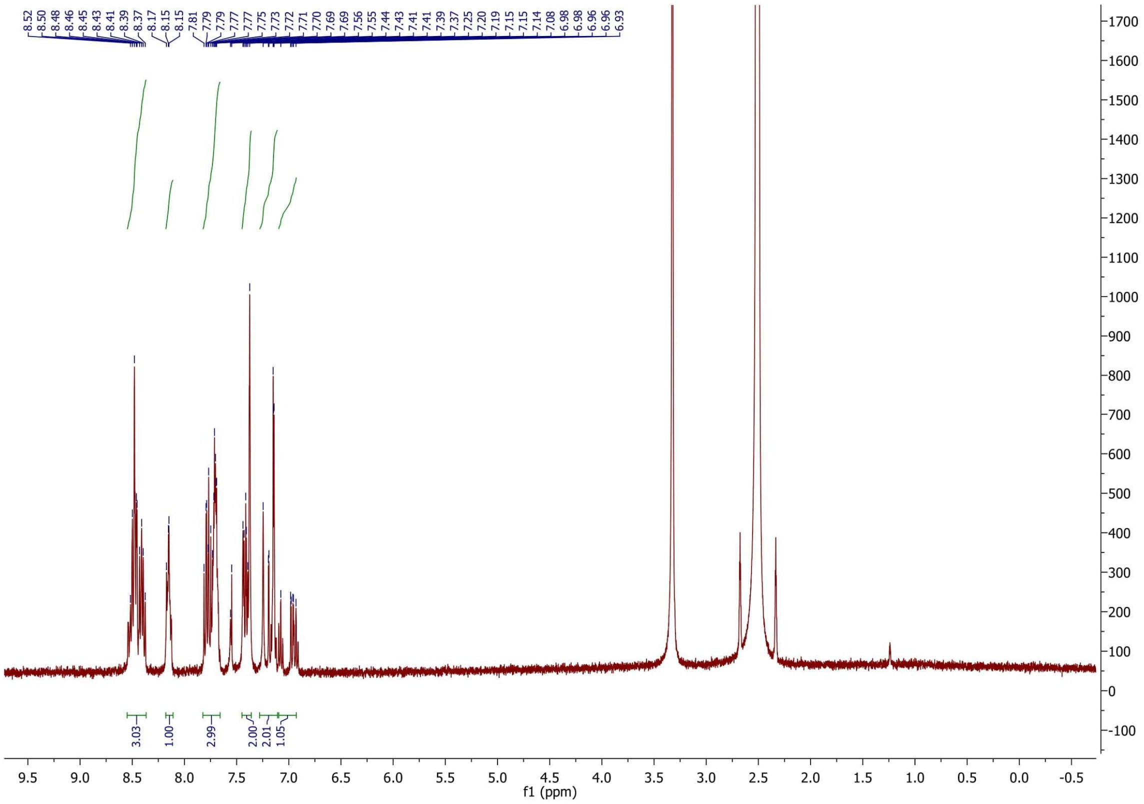

FT IR:

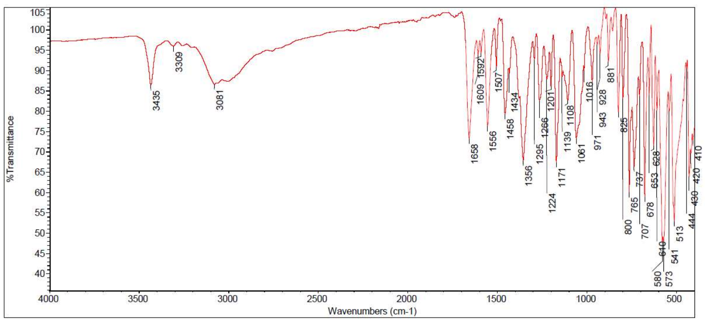

2.8. *N*-(1,4-dihydroquinazolin-2-yl)benzenesulfonamide 10e

UPLC-MS:

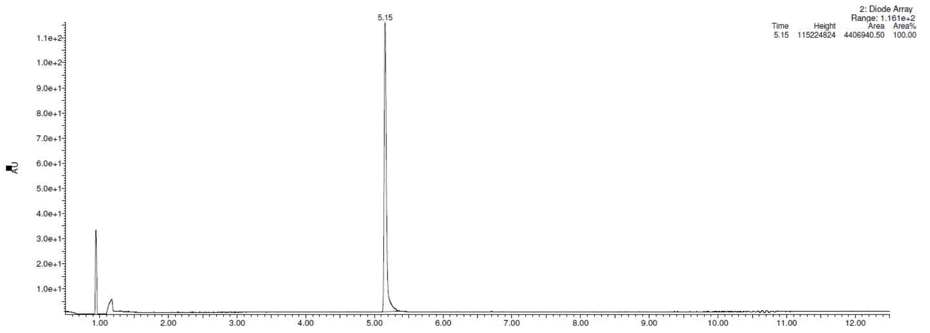

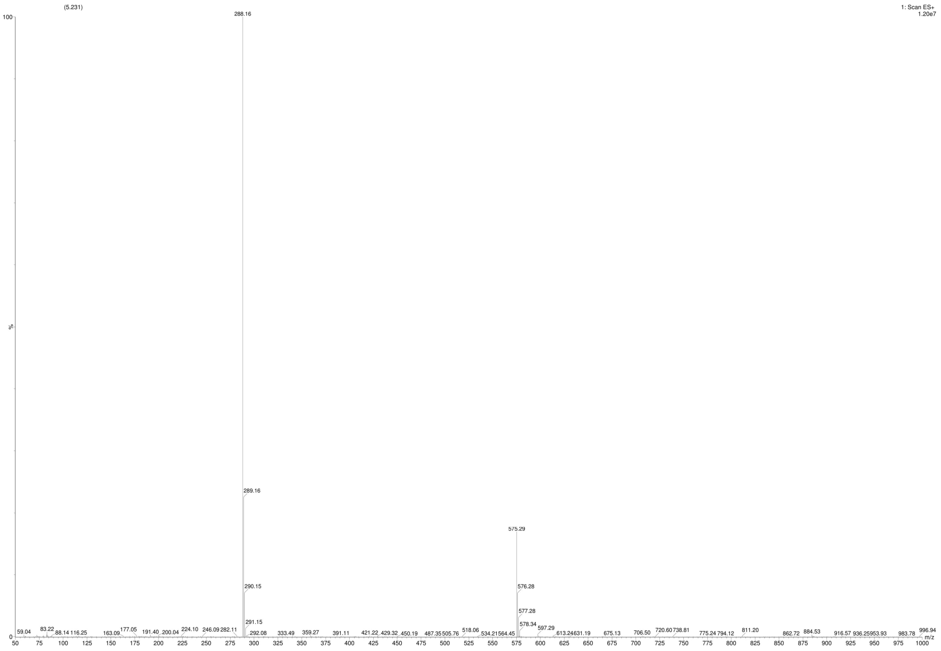

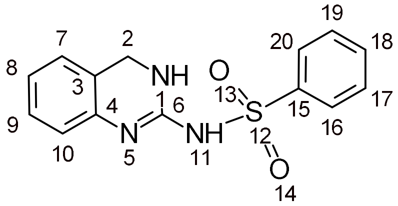

^{1}H NMR (400 MHz, DMSO) δ 10.01 (s, 1H $N^{1}H$), 8.78 (d, *J* = 8.6 Hz, 1H, $C^{18}H$), 8.24 (dd, *J* = 7.3, 1.2 Hz, 1H, $C^{23}H$), 8.14 (d, *J* = 8.2 Hz, 1H, $C^{22}H$), 8.04 (d, *J* = 7.5 Hz, 1H, $C^{20}H$), 7.93 (s, 1H $N^{11}H$), 7.68 (ddd, *J* = 8.5, 6.9, 1.5 Hz, 1H, $C^{16}H$), 7.64 – 7.58 (m, 2H, $C^{17}H$,$C^{21}H$), 7.14 (t, *J* = 7.7 Hz, 1H, $C^{8}H$), 7.09 (d, *J* = 7.5 Hz, 1H $C^{7}H$), 6.95 (td, *J* = 7.5, 1.0 Hz, 1H $C^{9}H$), 6.88 (d, *J* = 7.9 Hz, 1H, $C^{10}H$), 4.40 (s, 2H $C^{2}H$).

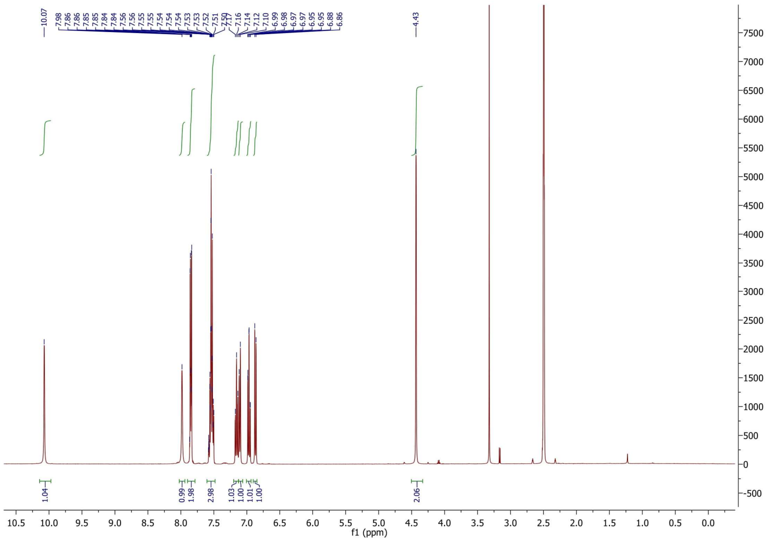

^{13}C NMR (101 MHz, DMSO) δ 152.10 C^{6}, 138.91 C^{4}, 134.98 C^{15}, 133.80 C^{19}, 132.65 C^{16}, 128.53 C^{24}, 128.05 C^{8}, 127.93 C^{9}, 127.22 C^{20}, 126.51 C^{18}, 126.32 C^{7}, 125.93 C^{22}, 125.81 C^{21}, 124.40 C^{10}, 123.12 C^{17}, 117.98 C^{23}, 114.68 C^{3}, 42.10 C^{2}.

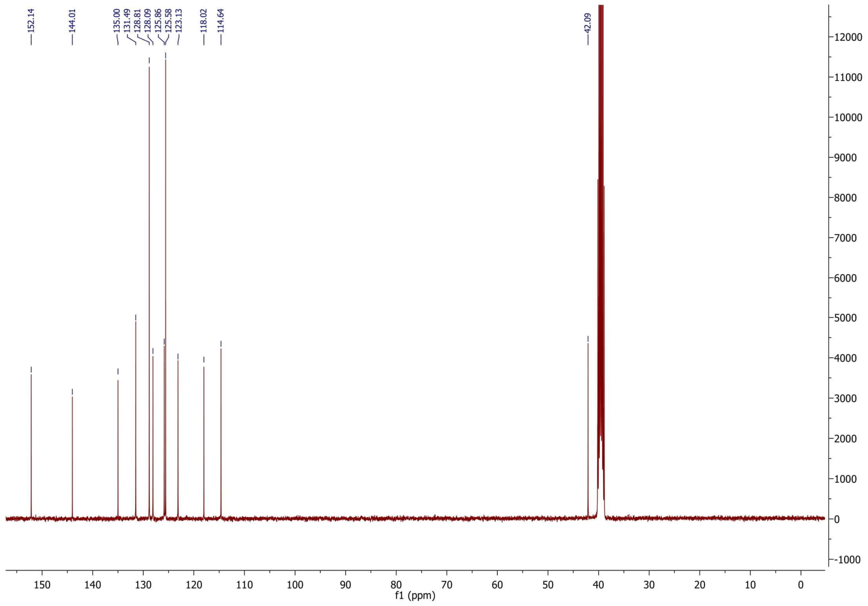

2.9. *N*-(1,4-dihydroquinazolin-2-yl)naphthalene-1-sulfonamide 10f

UPLC-MS:

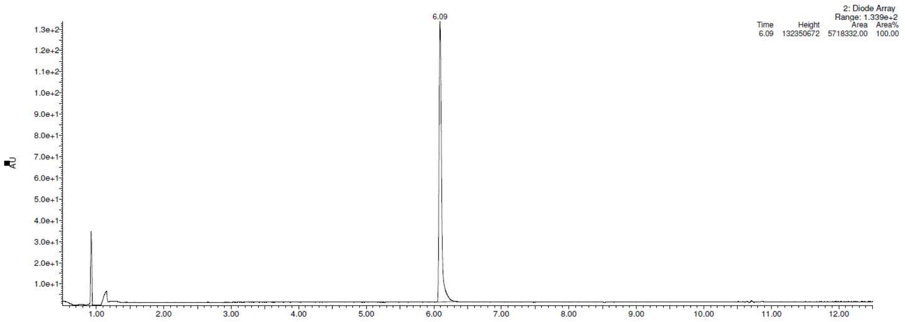

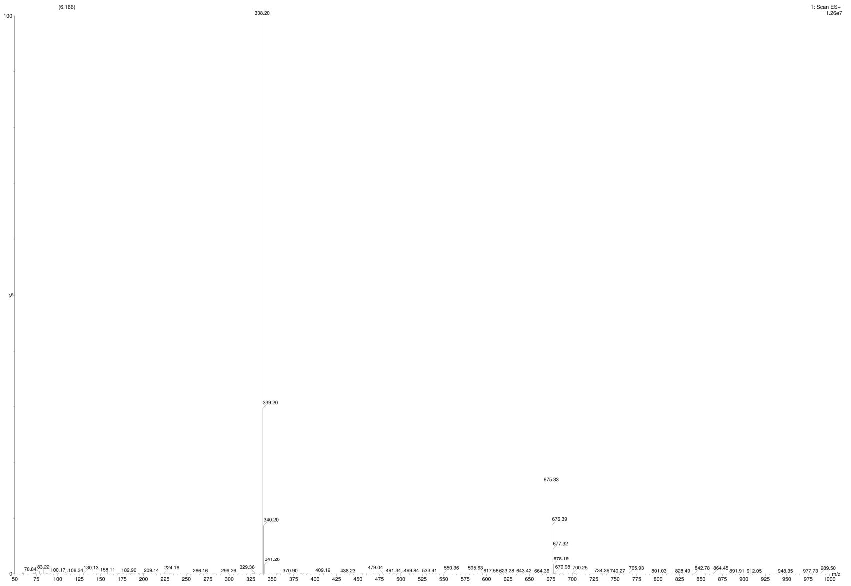

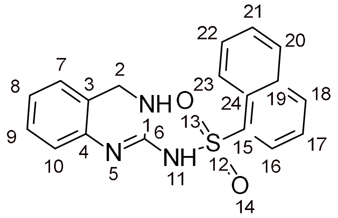

^{1}H NMR (400 MHz, DMSO) δ 10.07 (s, 1H N^{1}H), 7.98 (s, 1H NH11), 7.90 – 7.79 (m, 2H, C^{16}H,C^{20}H), 7.60 – 7.48 (m, 3H C^{17}H,C^{18}H,C^{19}H), 7.16 (t, *J* = 7.7 Hz, 1H, C^{8}H), 7.11 (d, *J* = 7.4 Hz, 1H C^{7}H), 6.97 (td, *J* = 7.5, 1.0 Hz, 1H, C^{9}H), 6.87 (d, *J* = 7.9 Hz, 1H, C^{10}H), 4.43 (s, 2H C^{2}H).

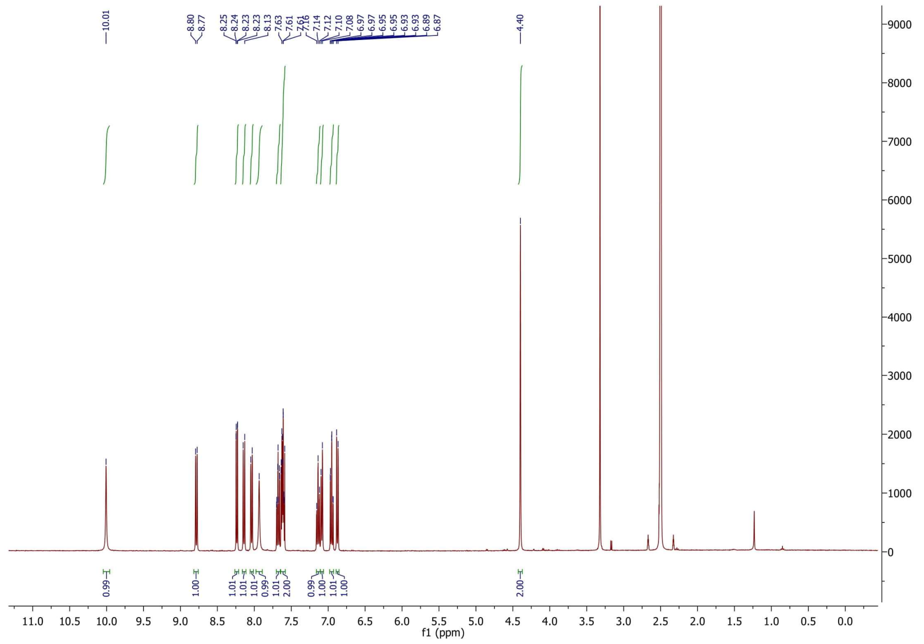

^{13}C NMR (101 MHz, DMSO) δ 152.14C^{6}, 144.01 C^{4}, 135.00 C^{15}, 131.49 C^{8}, 128.81 C^{17},C^{19}, 128.09 C^{9}, 125.86 C^{18}, 125.58 C^{16},C^{20}, 123.13 C^{7}, 118.02 C^{10}, 114.64 C^{3}, 42.09 C^{2}.

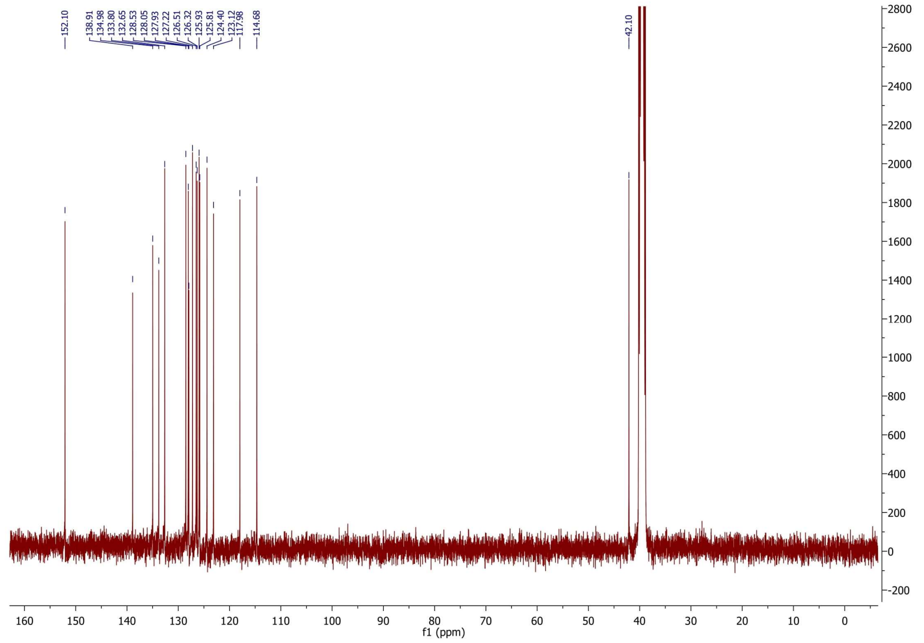

FT IR:

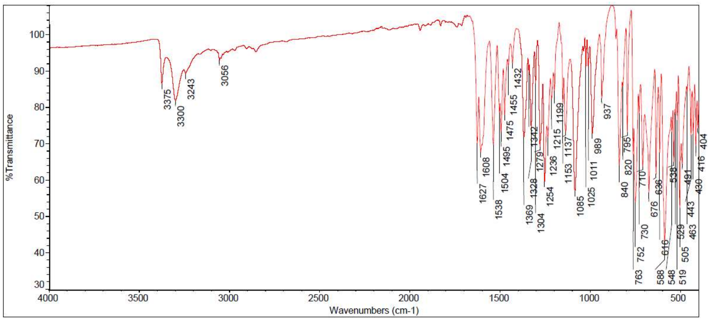

2.10. *N*-(1,4-dihydroquinazolin-2-yl)naphthalene-2-sulfonamide 10g

UPLC-MS:

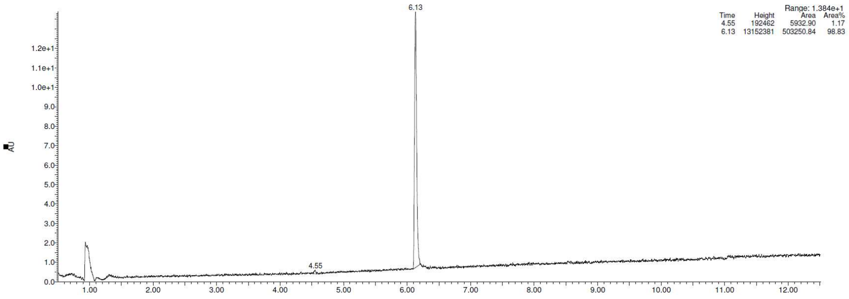

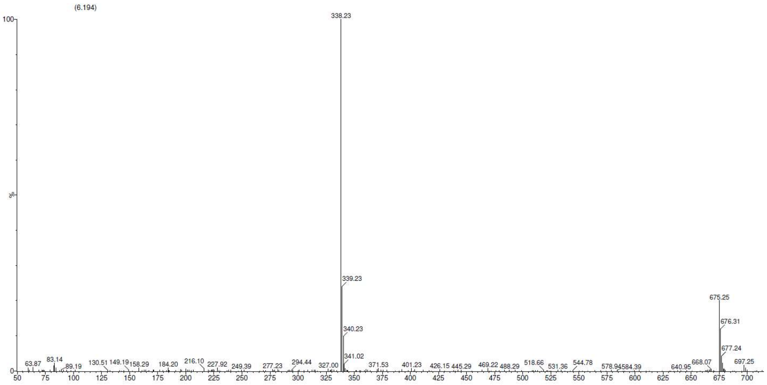

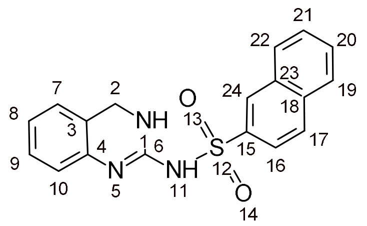

^{1}H NMR (400 MHz, DMSO) δ 8.47– 8.45 (m, 1H, C^{24}H), 8.08 (d, *J* = 8.0 Hz, 1H, C^{21}H), 7.99 (dd, *J* = 13.0, 8.9 Hz, 2H, C^{17}H,C^{19}H), 7.88 (dd, *J* = 8.6, 1.7 Hz, 1H, C^{16}H), 7.67 – 7.56 (m, 2H, C^{20}H,C^{21}H), 7.08– 7.02 (m, 1H, C^{9}H), 7.00 – 6.94 (m, 1H C^{8}H), 6.87 (d, *J* = 6.4 Hz, 1H C^{7}H), 6.78 (d, *J* = 5.2 Hz, 1H, C^{10}H), 4.38 (s, 2H, C^{2}H).

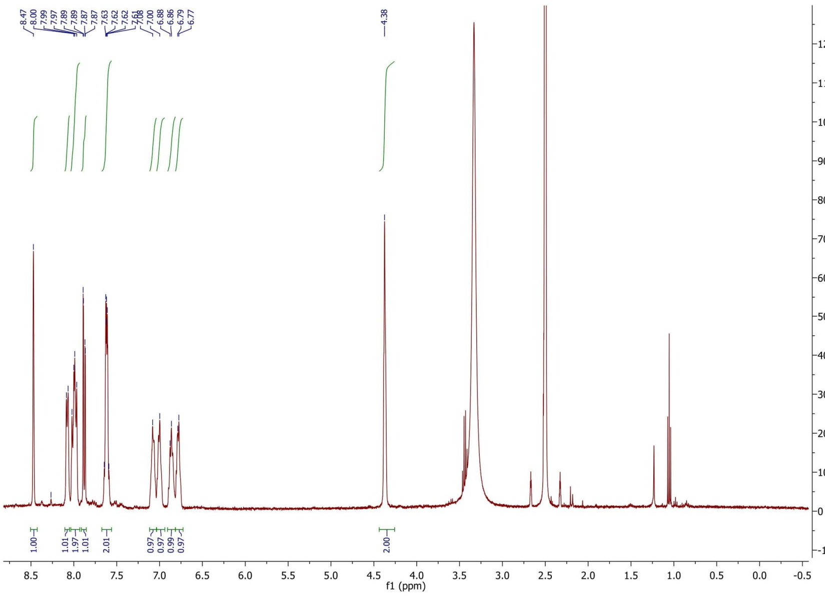

^{13}C NMR (101 MHz, DMSO) δ 133.59 C^{6}, 131.78 C^{4}, 128.89 C^{23},C^{15}, 128.31 C^{17},C^{18}, 127.80 C^{8},C^{9}, 127.66 C^{22},C^{19}, 127.06 C^{7},C^{16}, 125.74 C^{20},C^{21}, 125.52 C^{10}, 122.93 C^{24}, 118.72 C^{3}, 42.48 C^{2}.

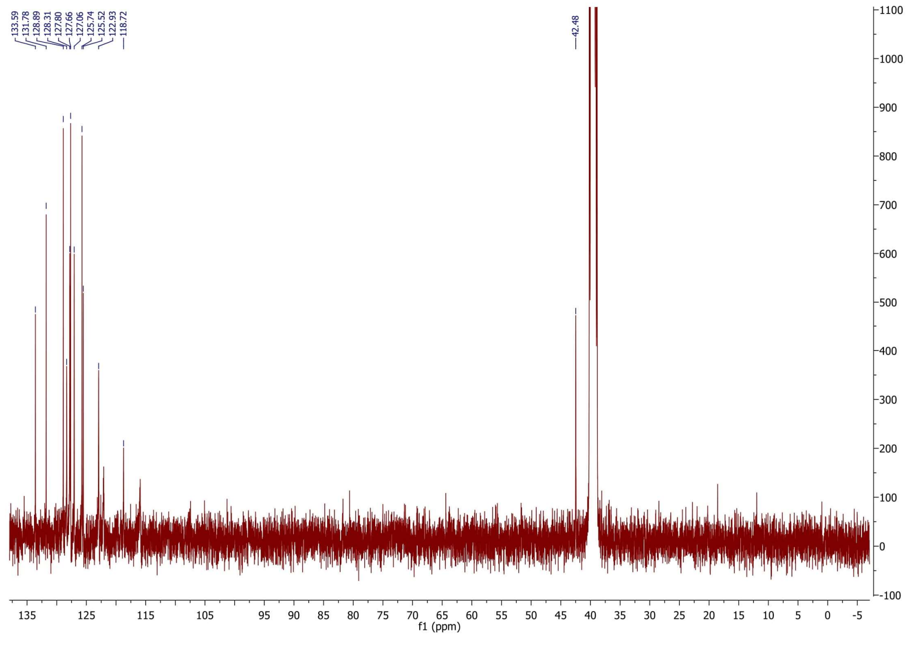

FT IR:

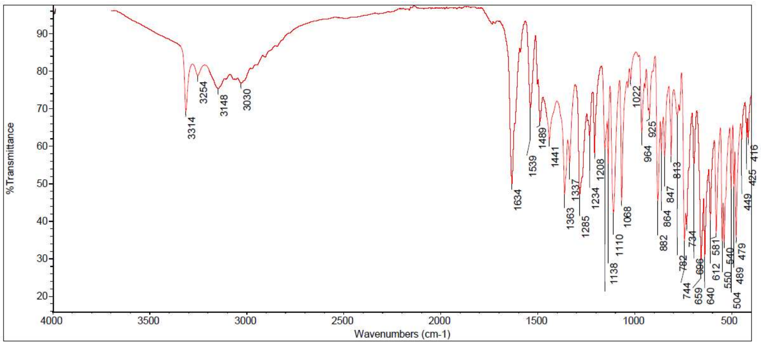

2.11. *N*-(6-chloro-1,4-dihydroquinazolin-2-yl)naphthalene-2-sulfonamide 10h

UPLC-MS:

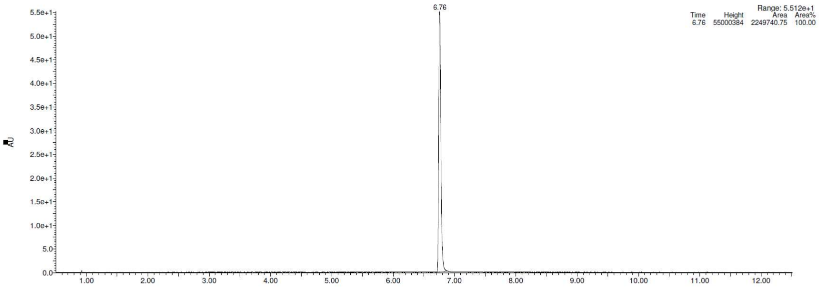

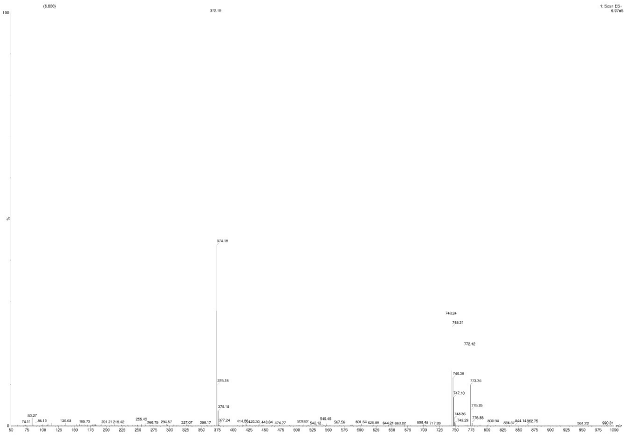

FT IR:

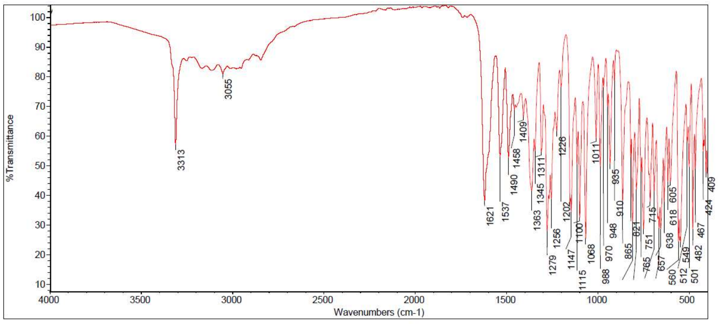

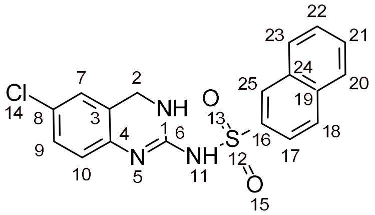

^{1}H NMR (400 MHz, DMSO) δ 8.46 (s, 1H, C^{25}H), 8.09 – 8.04 (m, 1H, C^{23}H), 7.98 (t, *J* = 8.5 Hz, 2H, C^{18}H,C^{20}H), 7.88 (dd, *J* = 8.6, 1.7 Hz, 1H, C^{17}H), 7.63 – 7.55 (m, 2H, C^{21}H, C^{22}H), 7.13 – 6.98 (m, 2H, C^{7}H,C^{9}H), 6.72 (d, *J* = 8.2 Hz, 1H, C^{10}H), 4.33 (s, 2H, C^{2}H).

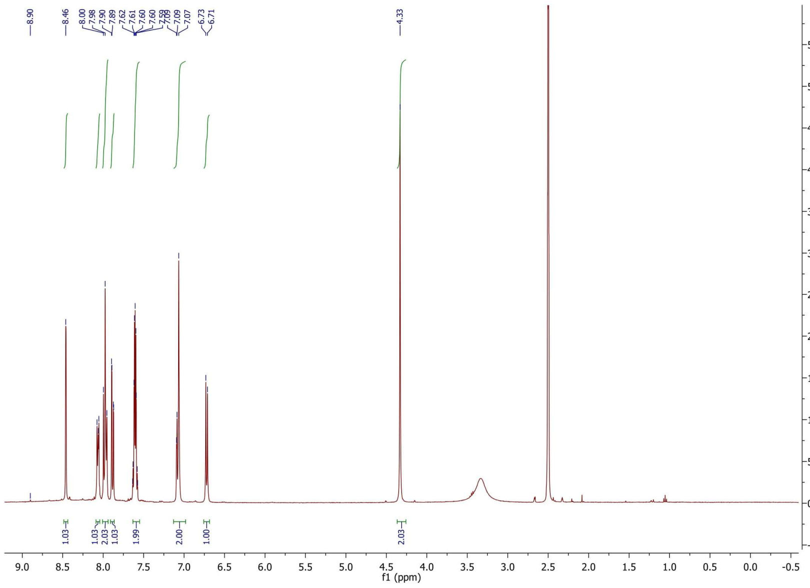

^{13}C NMR (101 MHz, DMSO) δ 153.99 C^{6}, 142.74 C^{4}, 133.99 C^{16}, 132.25 C^{24}, 129.32 C^{18},C^{19}, 128.51 C^{8}, 128.13 C^{7},C^{9}, 128.09 C^{23}, 127.86 C^{20}, 127.41 C^{17}, 126.36 C^{10}, 125.63 C^{22}, 125.27 C^{21}, 123.71 C^{25}, 121.64 C^{3}, 118.65 C^{2}.

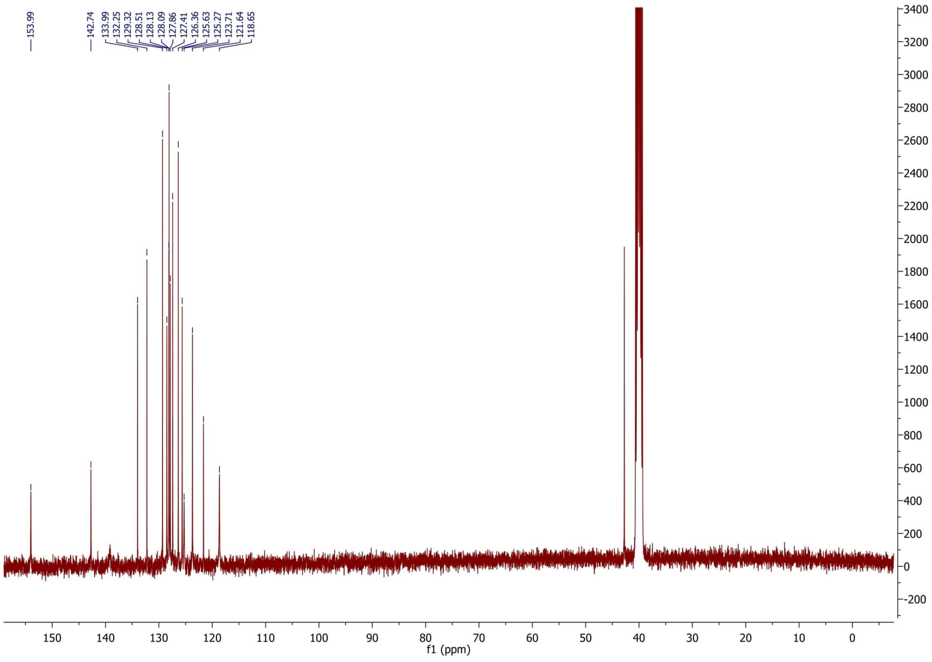

2.12. 1-(benzenesulfonyl)-1,4-dihydroquinazolin-2-amine 11e

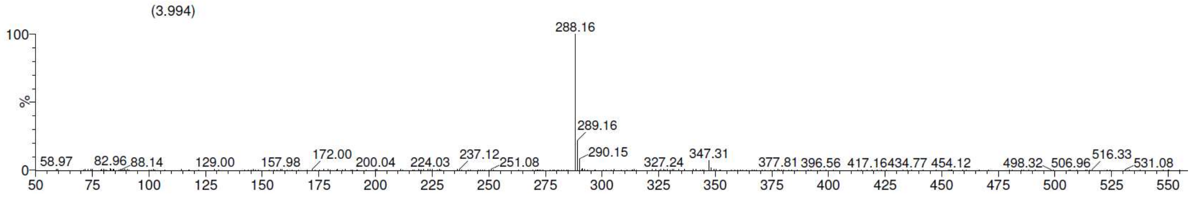

2.13. 1-(naphthalene-1-sulfonyl)-1,4-dihydroquinazolin-2-amine 11h

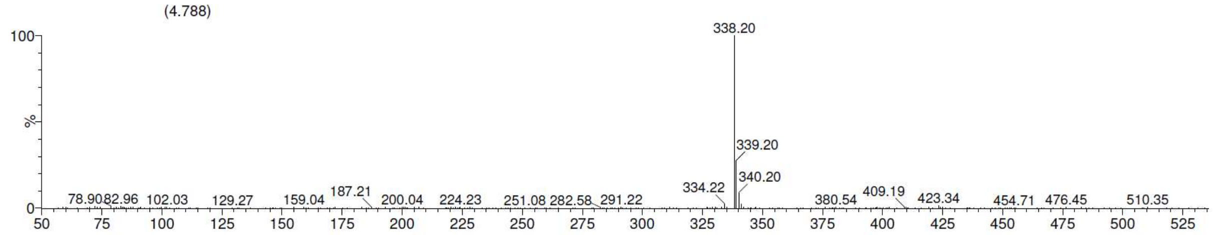

3. ADME-tox 10f

3.1. Permeability – PAMPA

UPLC of the solution of reference compound caffeine in PBS (pH=7.4) after 5h of incubation (control reaction). IS = internal standard:

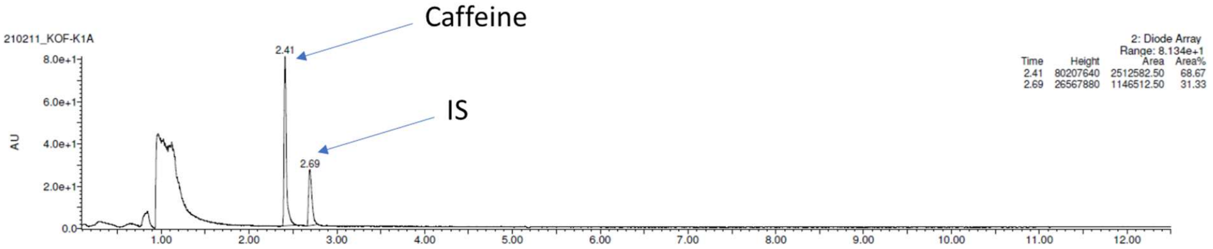

UPLC of caffeine solution in PBS (pH=7.4) in acceptor and donor wells after 5h of incubation:

Acceptor well

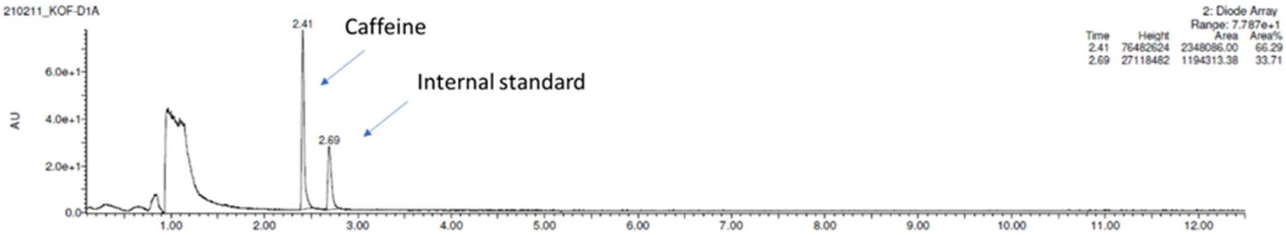

Donor well

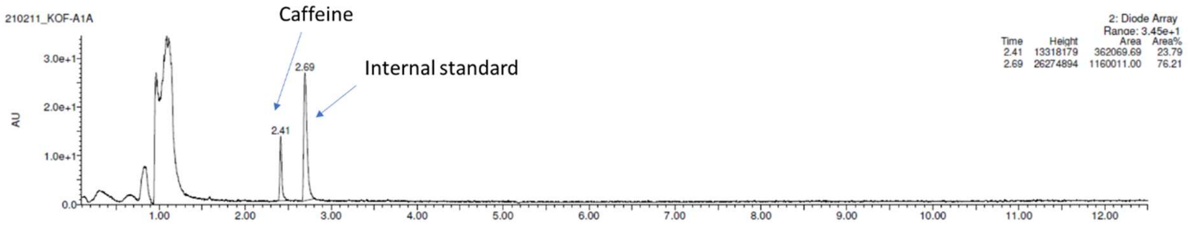

UPLC of **10f** solution in PBS (pH=7.4) after 5h of incubation (control reaction). IS = internal standard:

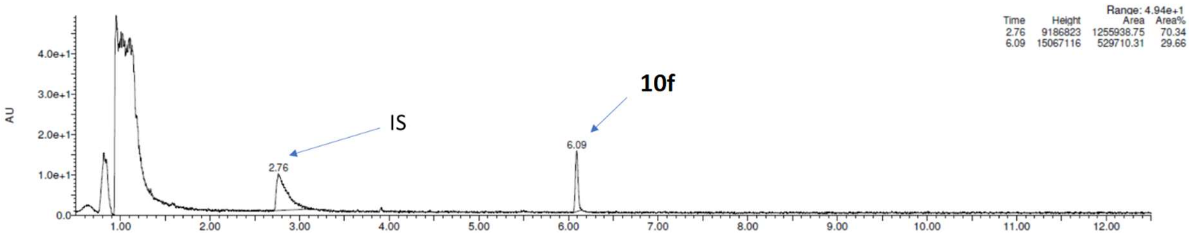

UPLC of **10f** solution in PBS (pH=7.4) in acceptor and donor wells after 5h of incubation:

Acceptor well

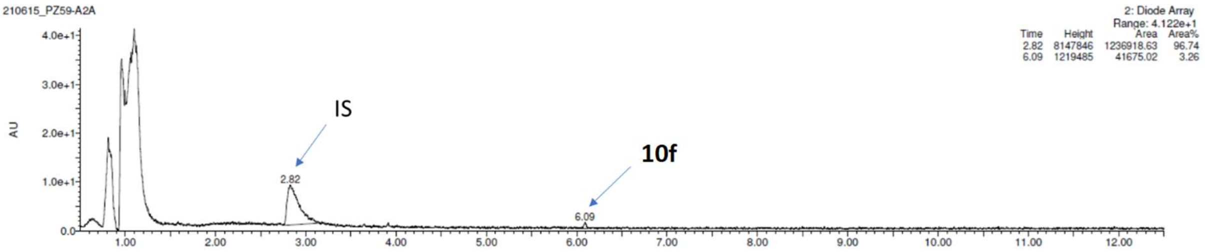

Donor well

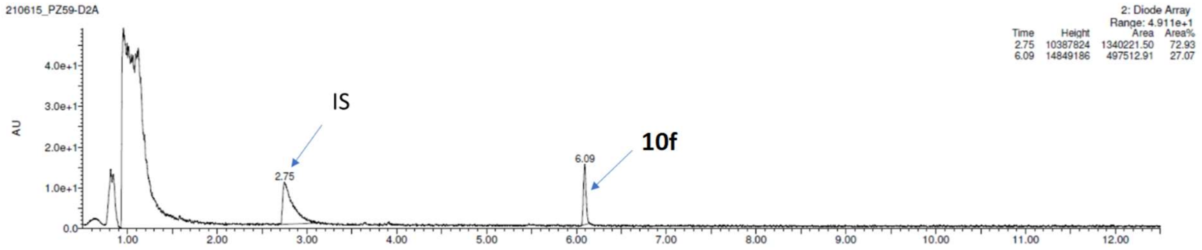

3.2. Metabolic stability

UPLC of control reaction (120 min incubation of compound **10f** in the reaction buffer without microsomes):

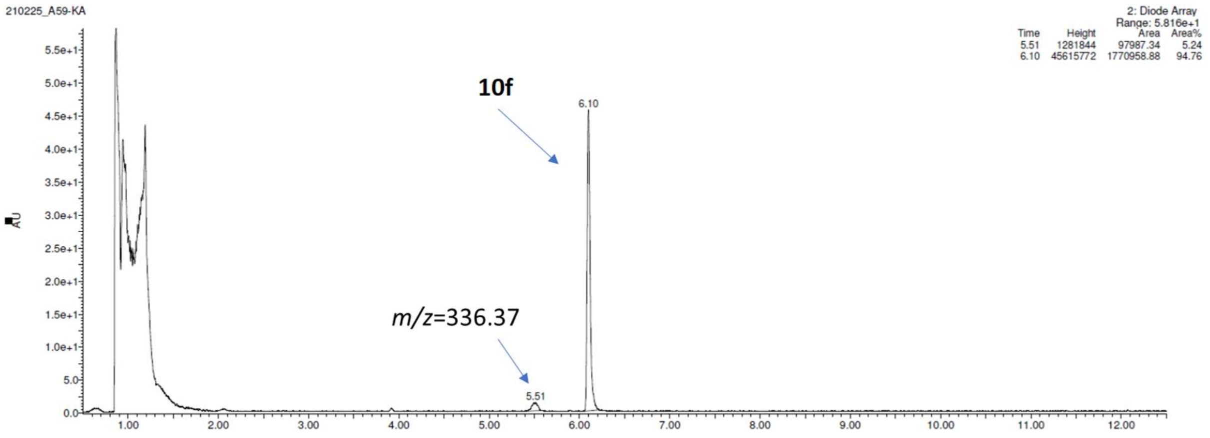

UPLC after 120 min incubation of compound **10f** with MLMs:

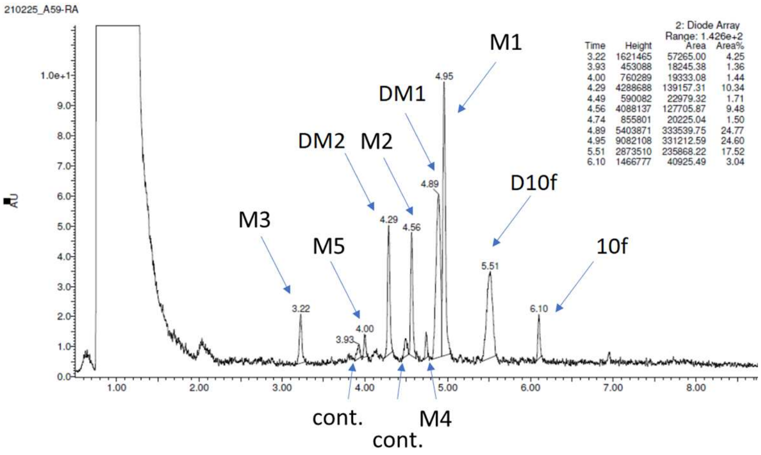

MS analyses of **10f** and its metabolites:

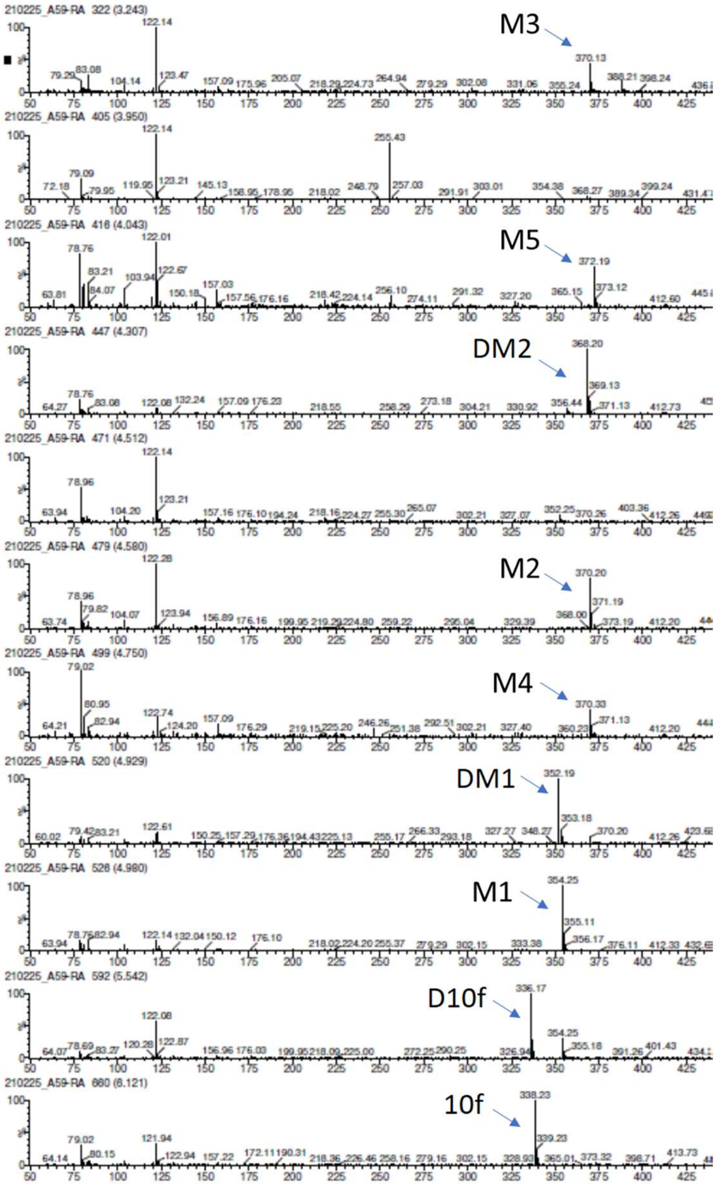

4. Development of synthesis methods (tables 3-5) - chromatographic analyzes

4.1. Development the method A of synthesis (Table 3)

Entry 1:

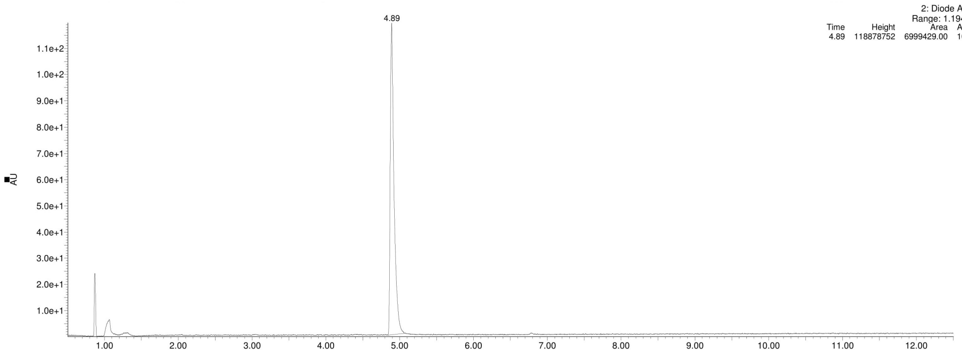

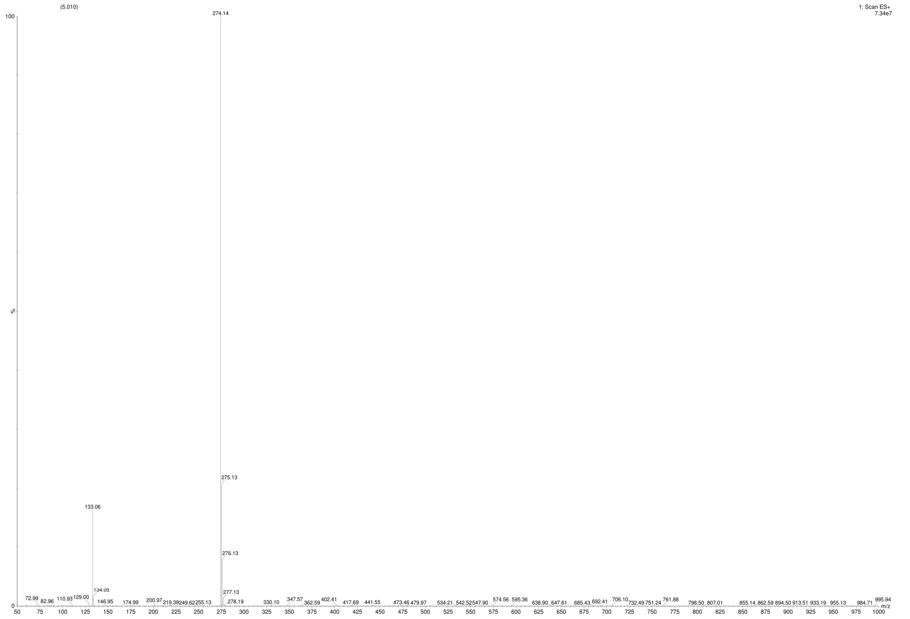

Entry 2:

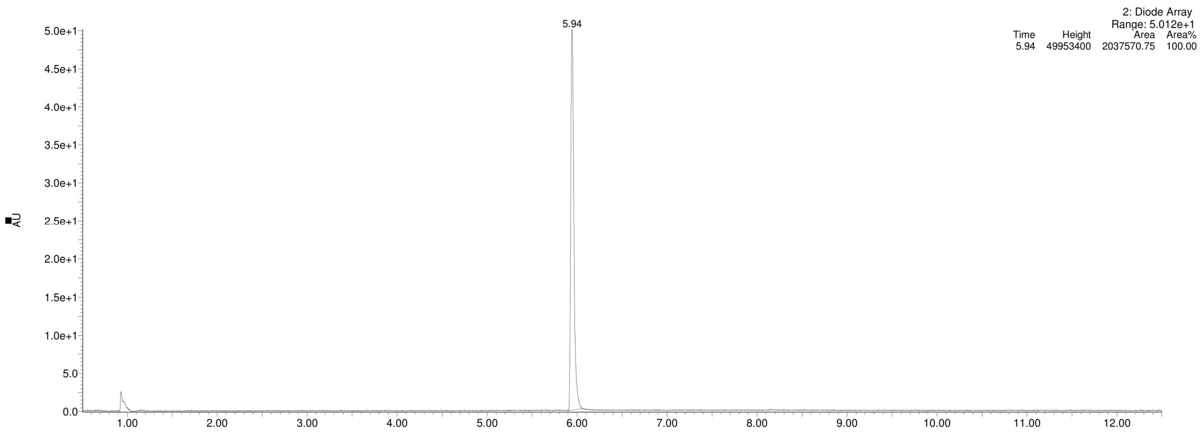

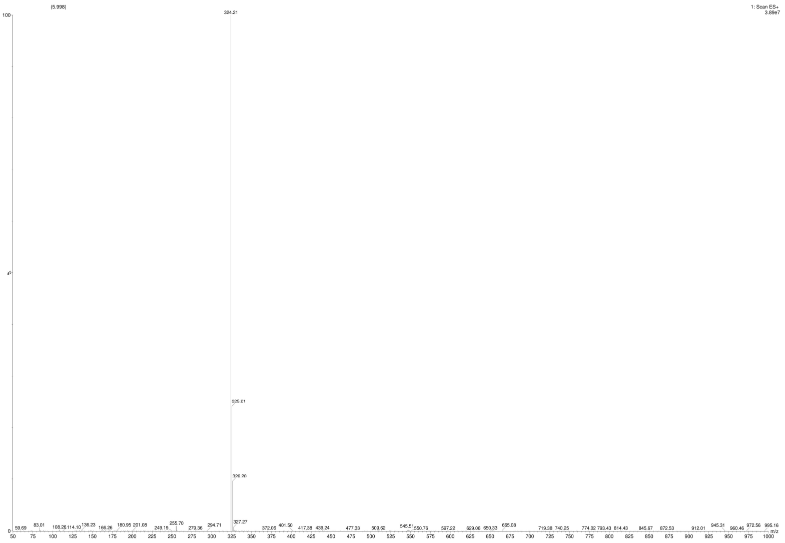
(5.998)
1: Scan ES+
3.89e7
324.21
325.21
326.20
m/z

Entry 3:

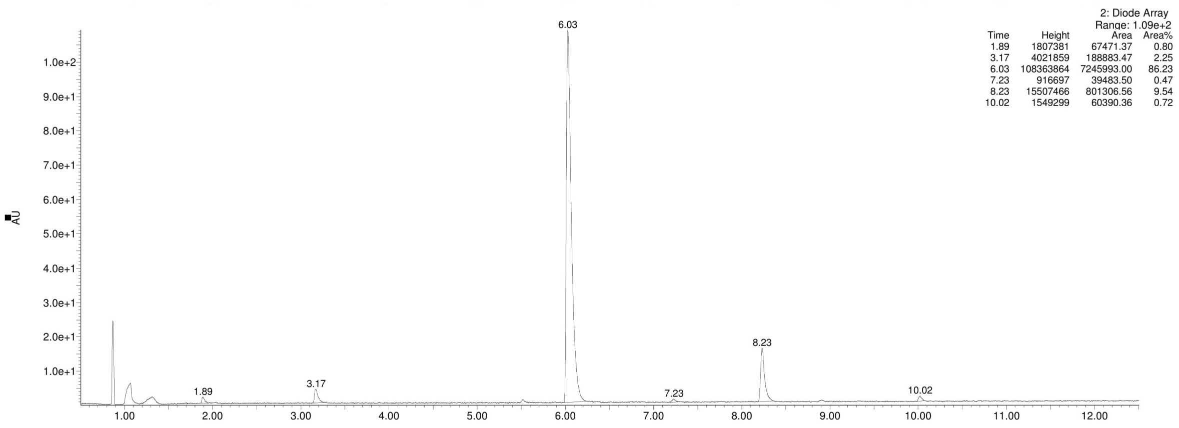
2: Diode Array
Range: 1.09e+2
Time Height Area Area%
1.89 1807381 67471.37 0.80
3.17 4021859 188883.47 2.25
6.03 108363864 7245993.00 86.23
7.23 916697 39483.50 0.47
8.23 15507466 801306.56 9.54
10.02 1549299 60390.36 0.72
AU
6.03
8.23
1.89
3.17
7.23
10.02

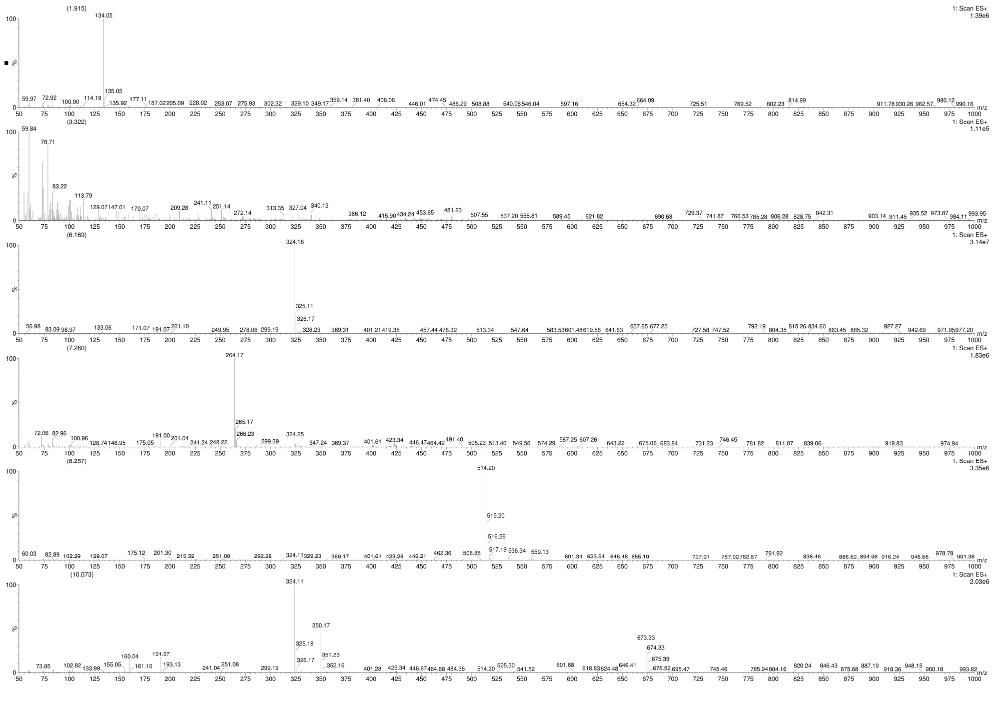
(1.915)
134.05
(3.322)
(6.169)
324.18
(7.260)
264.17
(8.257)
514.20
(10.073)
324.11
1: Scan ES+
m/z

Entry 4:

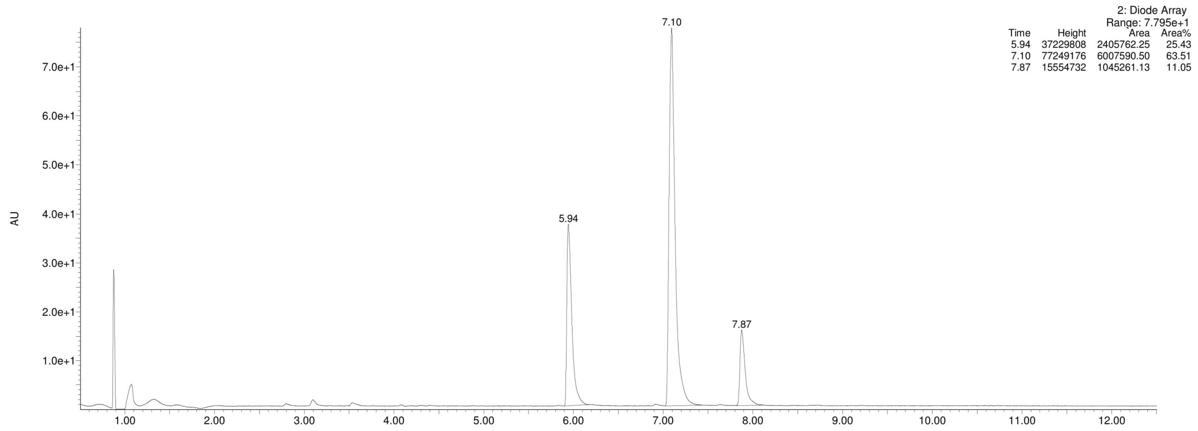

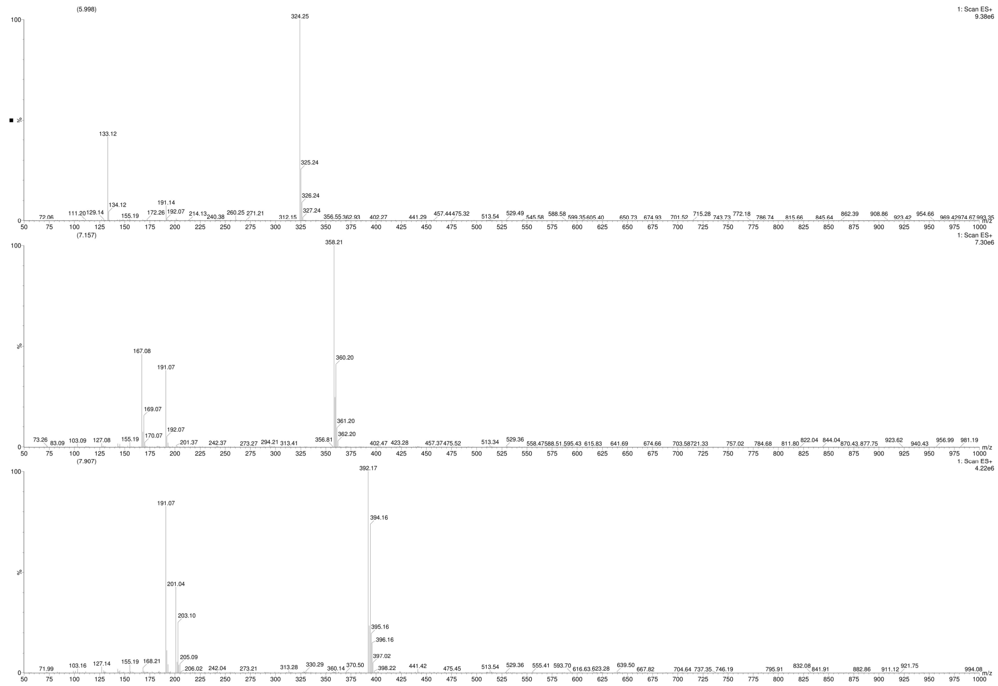

Entry 5:

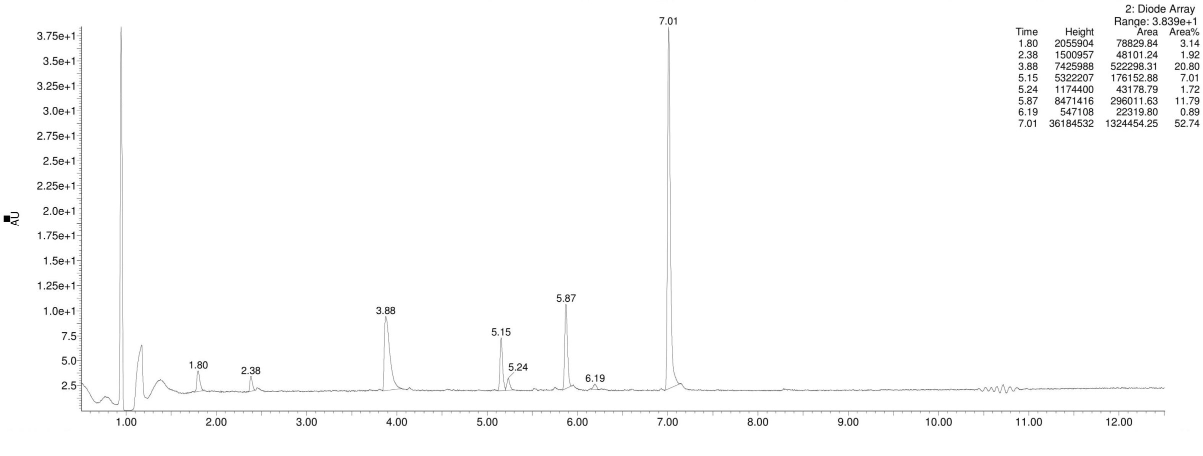

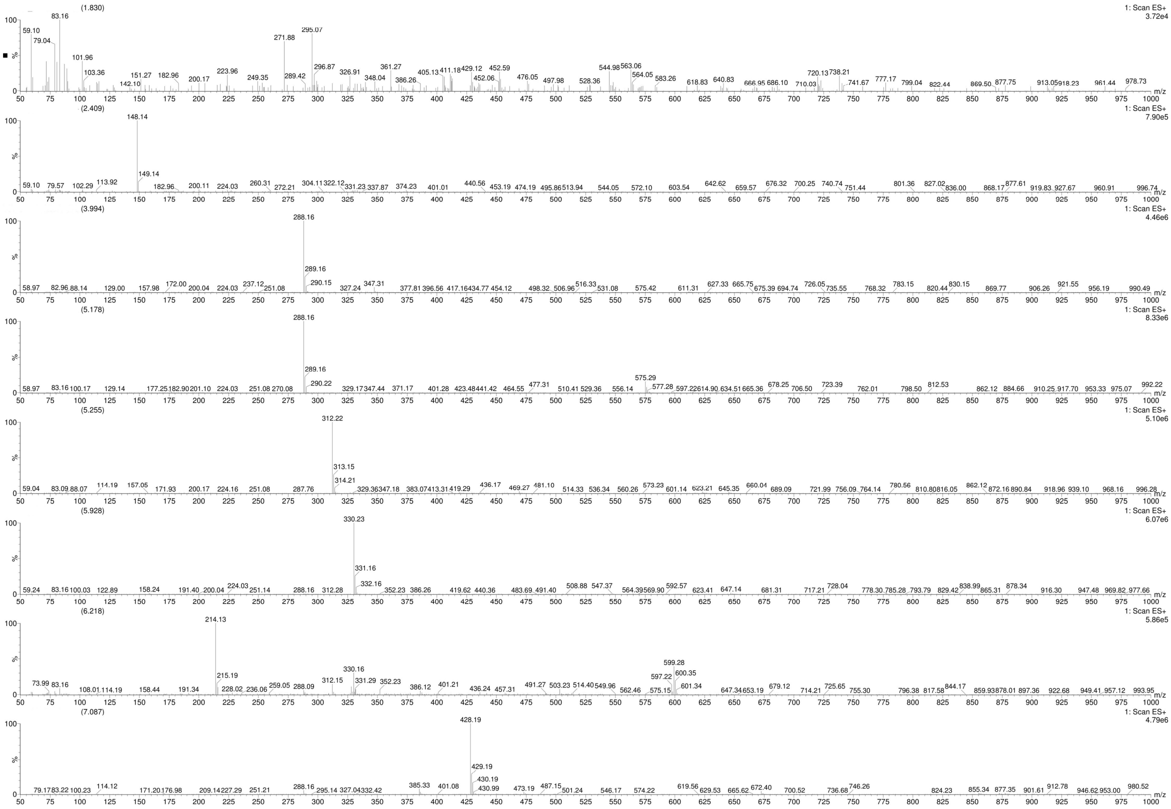

Entry 6:

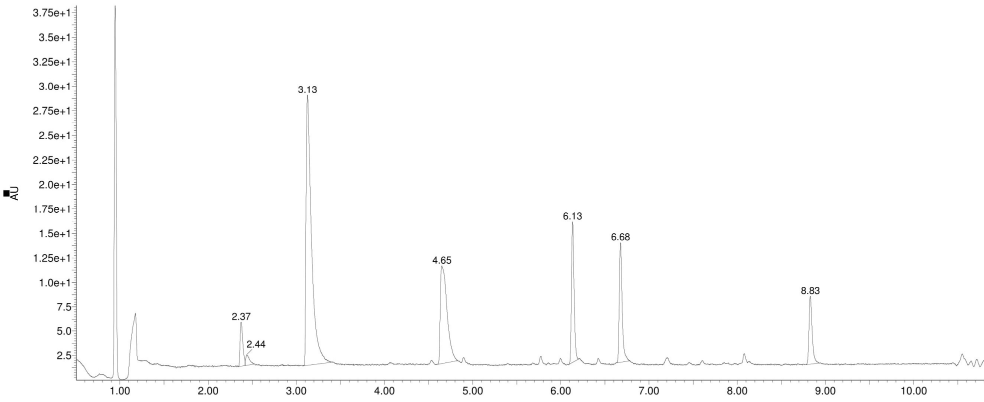

Time	Height	Area	Area%
2.37	4559881	162959.88	3.77
2.44	1029433	54141.45	1.25
3.13	27619698	1981371.75	45.88
4.65	10019559	881007.69	20.40
6.13	14410737	488051.41	11.30
6.68	12262507	463950.84	10.74
8.83	6986707	287399.06	6.65

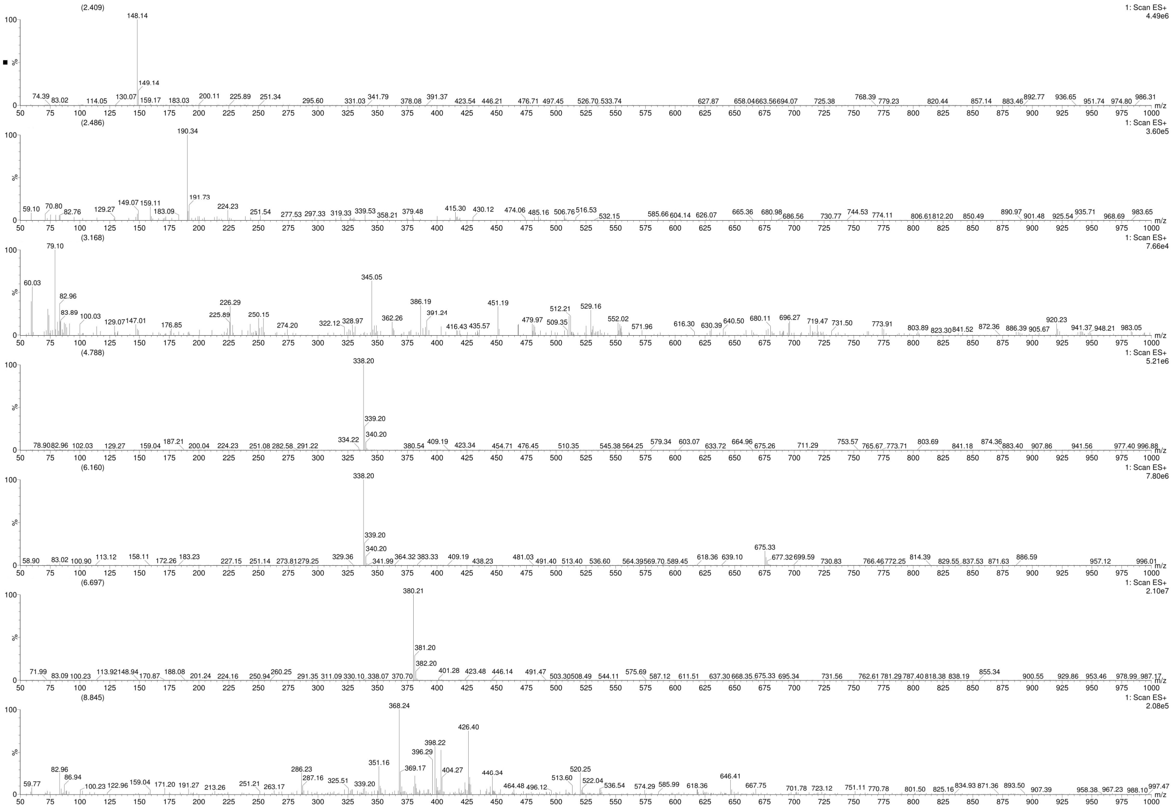

Entry 7:

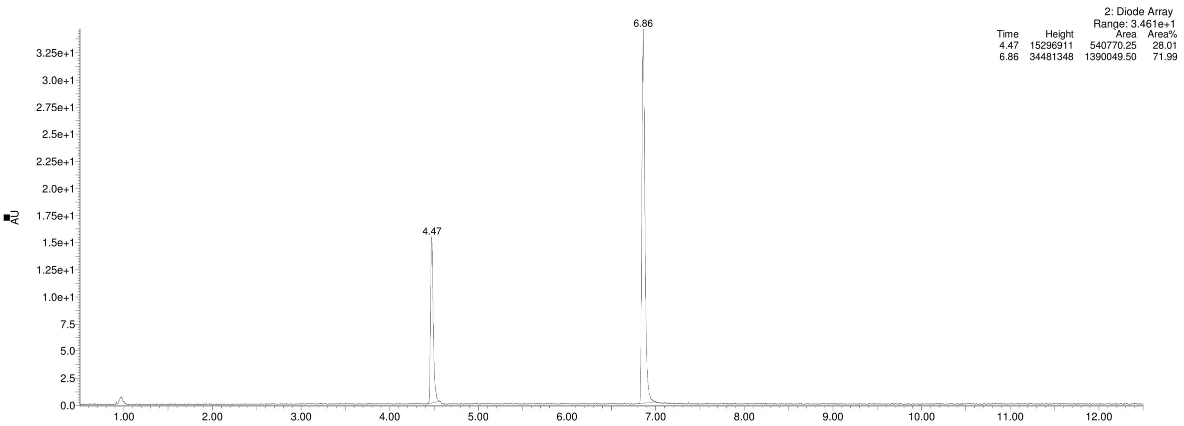

2: Diode Array
Range: 3.461e+1
Time Height Area Area%
4.47 15296911 540770.25 28.01
6.86 34481348 1390049.50 71.99
6.86
4.47
AU

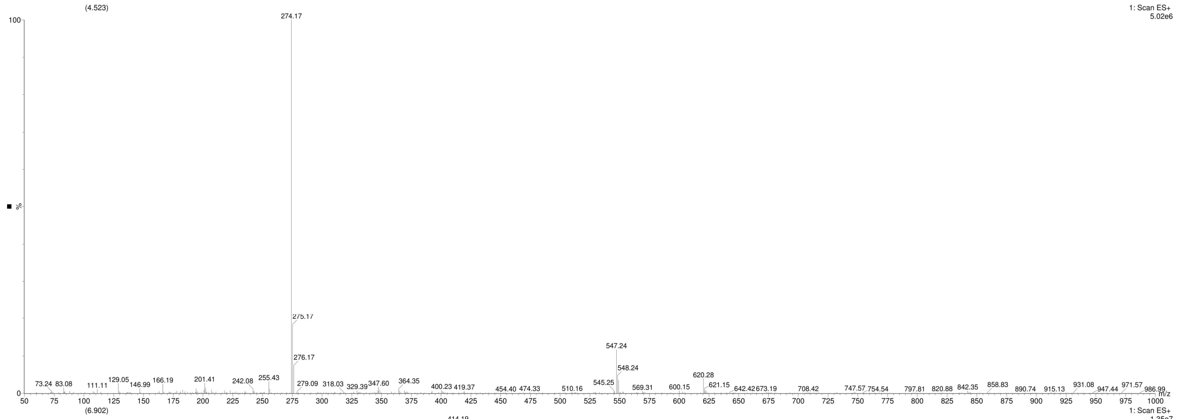

(4.523)
1: Scan ES+
5.02e6
274.17
275.17
276.17
547.24
548.24
620.28

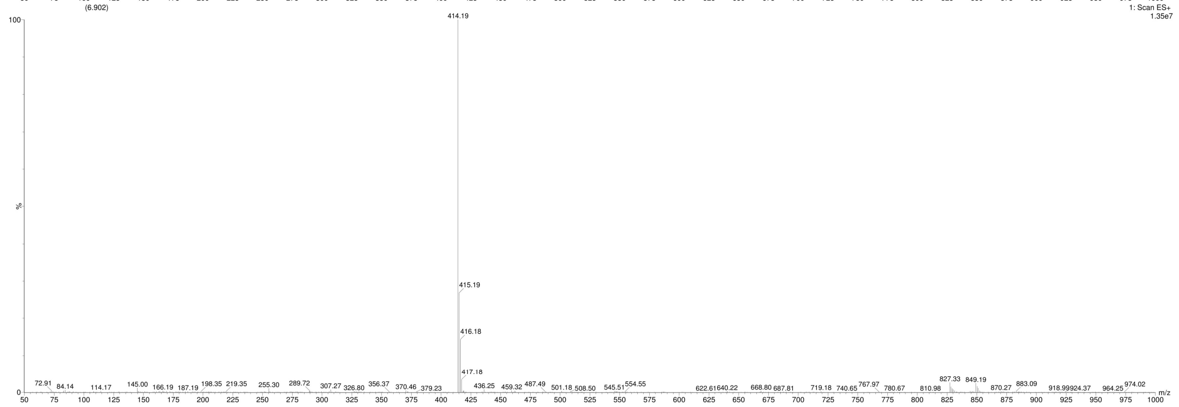

(6.902)
1: Scan ES+
1.35e7
414.19
415.19
416.18
417.18

Entry 8:

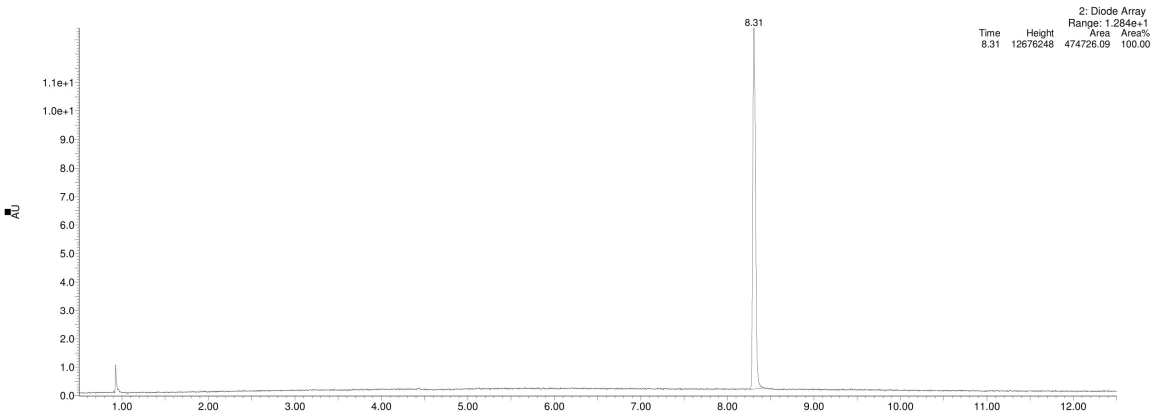

2: Diode Array
Range: 1.284e+1
Time Height Area Area%
8.31 12676248 474726.09 100.00
8.31
AU

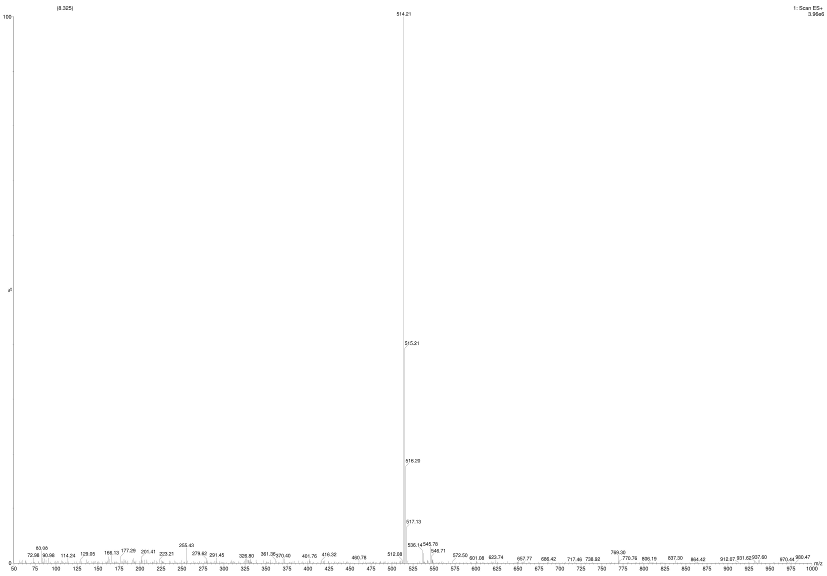
(8.325)
1: Scan ES+
3.96e6
514.21
515.21
516.20
517.13
m/z

Entry 9:

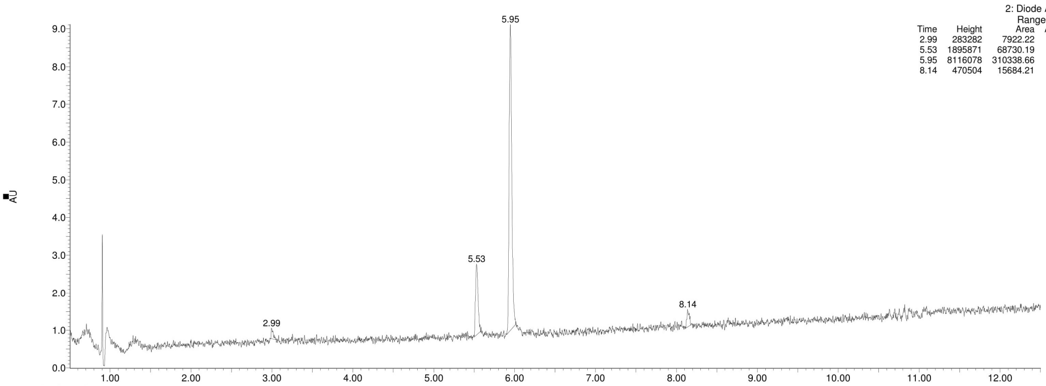
2: Diode Array
Range: 9.06
Time Height Area Area%
2.99 283282 7922.22 1.97
5.53 1895871 68730.19 17.07
5.95 8116078 310338.66 77.07
8.14 470504 15684.21 3.90
AU
5.95
5.53
2.99
8.14

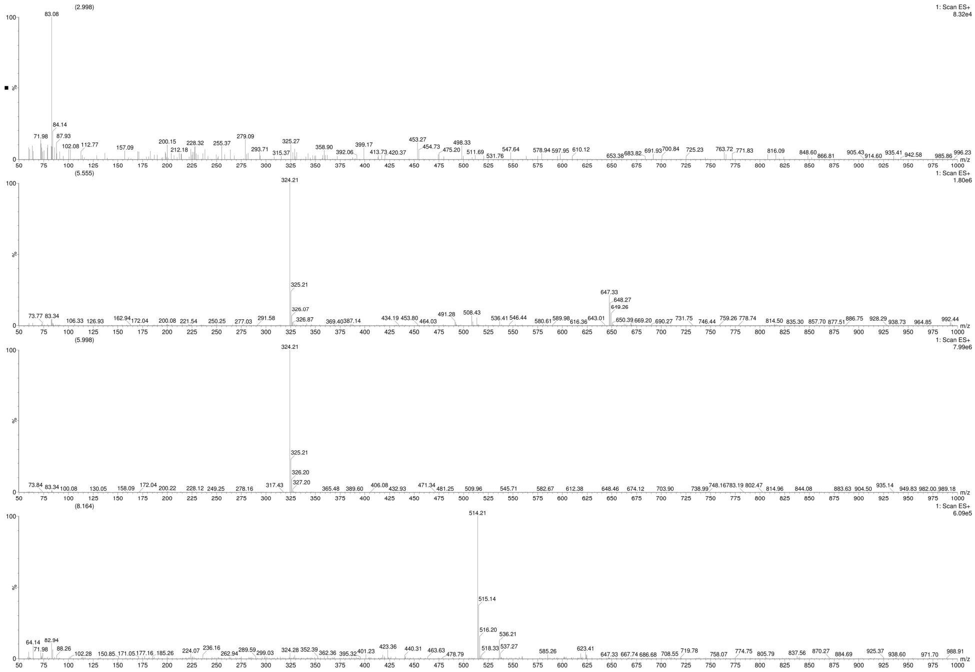
(2.998)
1: Scan ES+
8.32e4
83.08
(5.555)
1: Scan ES+
1.80e6
324.21
647.33
(5.998)
1: Scan ES+
7.99e6
324.21
(8.164)
1: Scan ES+
6.09e5
514.21
m/z

4.2. Development the method B of synthesis (Table 4)

3a standard:

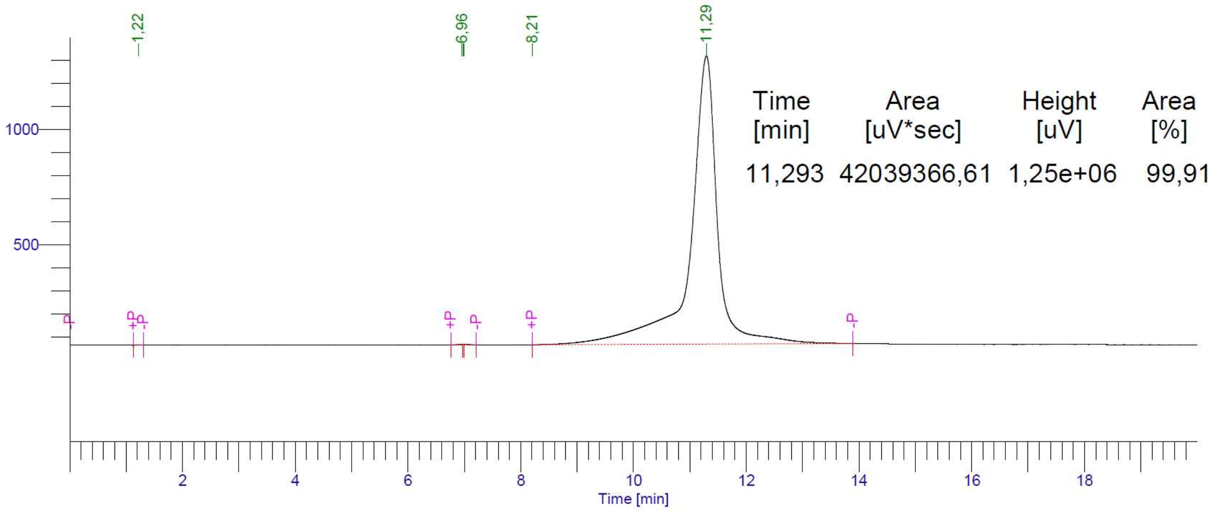

4a standard:

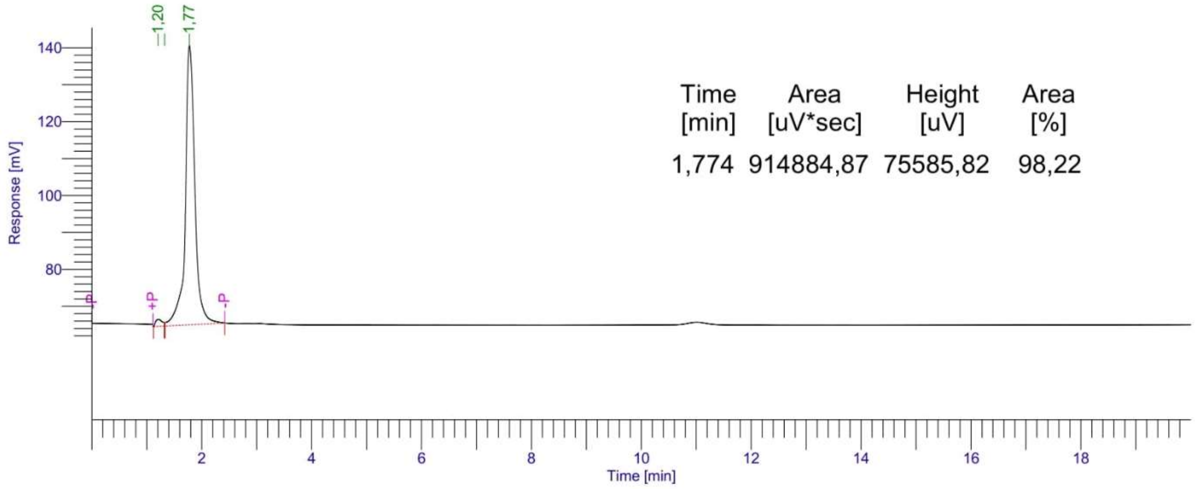

10a standard:

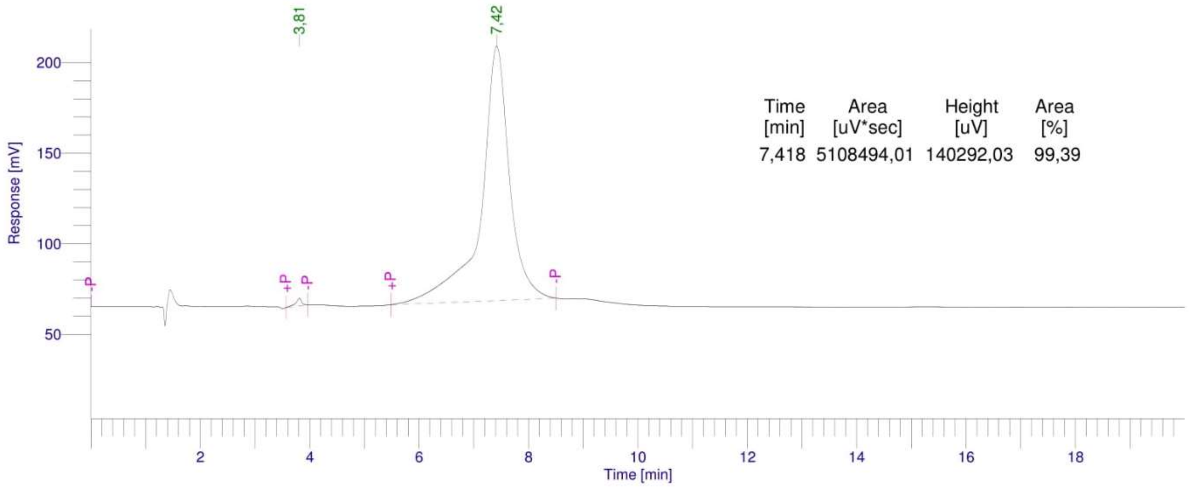

Entry 1:

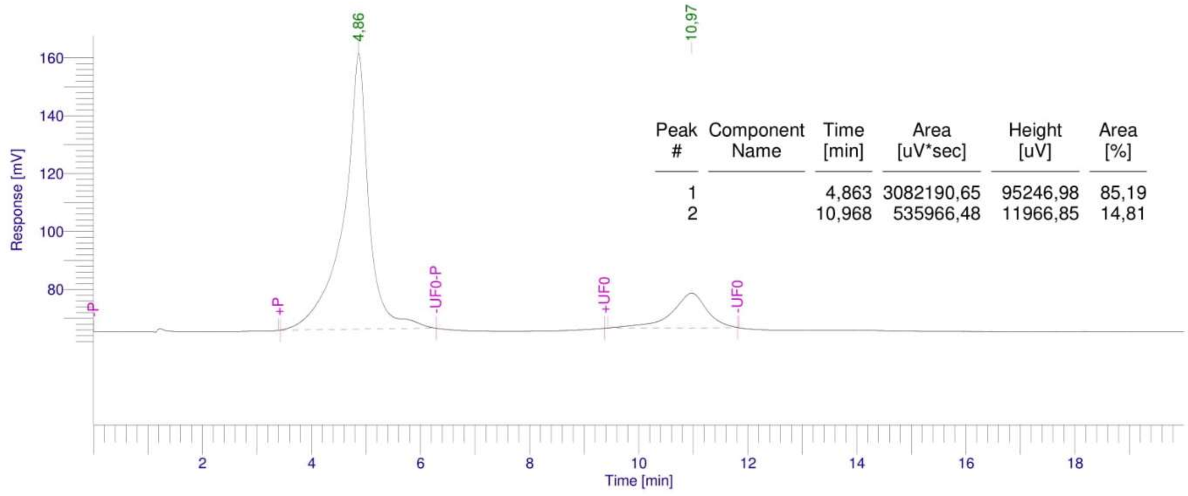

Peak #	Component Name	Time [min]	Area [uV*sec]	Height [uV]	Area [%]
1		4,863	3082190,65	95246,98	85,19
2		10,968	535966,48	11966,85	14,81

Entry 2:

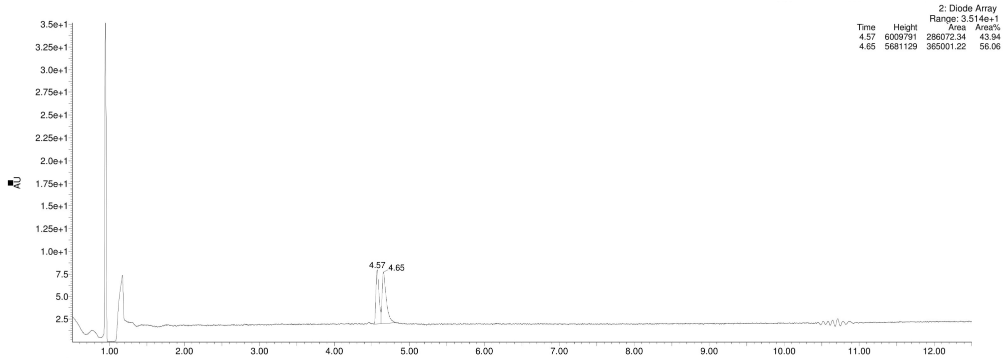

Time	Height	Area	Area%
4.57	6009791	286072.34	43.94
4.65	5681129	365001.22	56.06

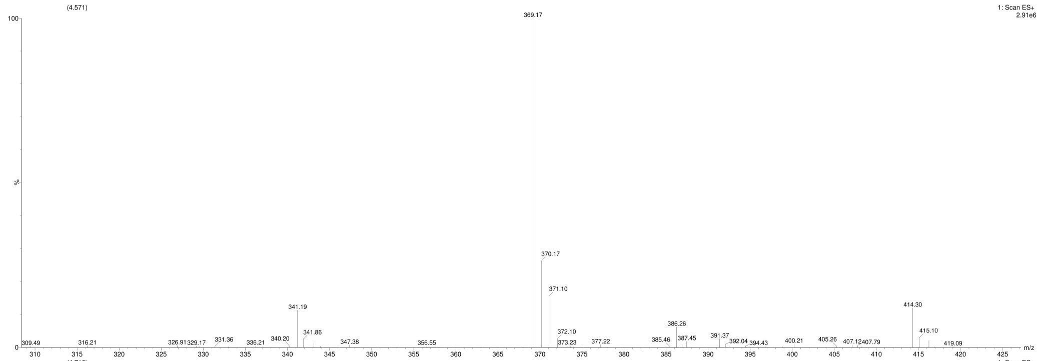

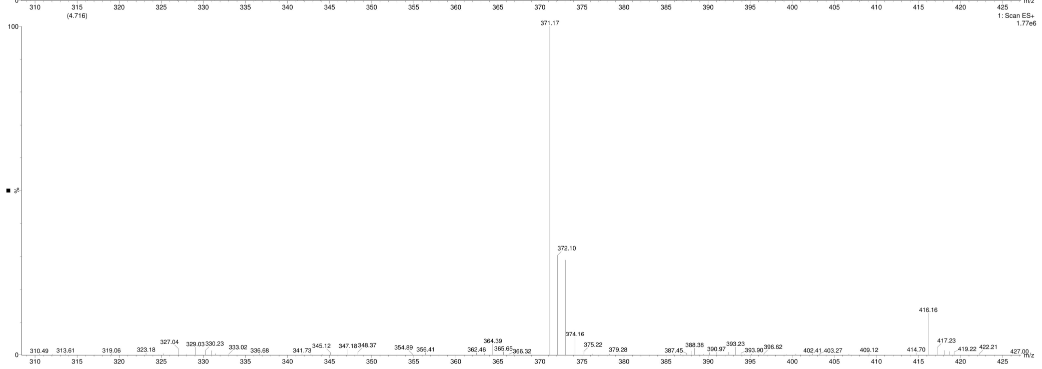

Entry 3:

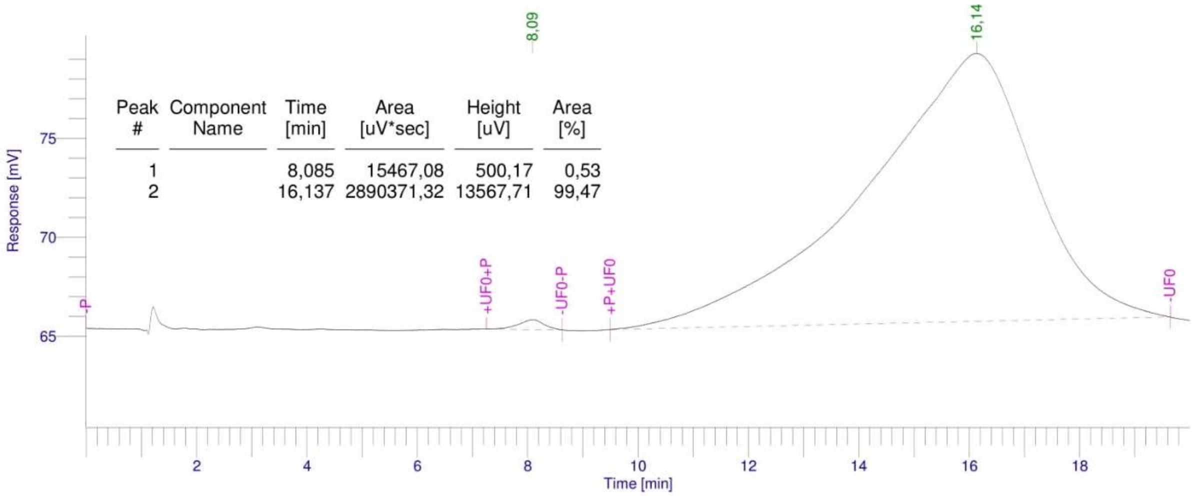

Peak #	Component Name	Time [min]	Area [uV*sec]	Height [uV]	Area [%]
1		8,085	15467,08	500,17	0,53
2		16,137	2890371,32	13567,71	99,47

Entry 4:

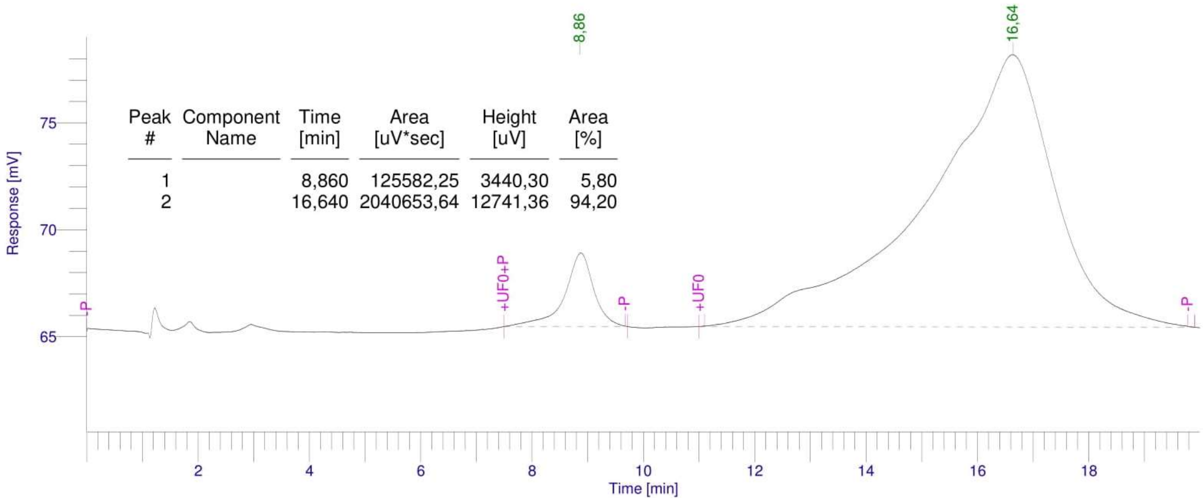

Peak #	Component Name	Time [min]	Area [uV*sec]	Height [uV]	Area [%]
1		8,860	125582,25	3440,30	5,80
2		16,640	2040653,64	12741,36	94,20

Entry 5:

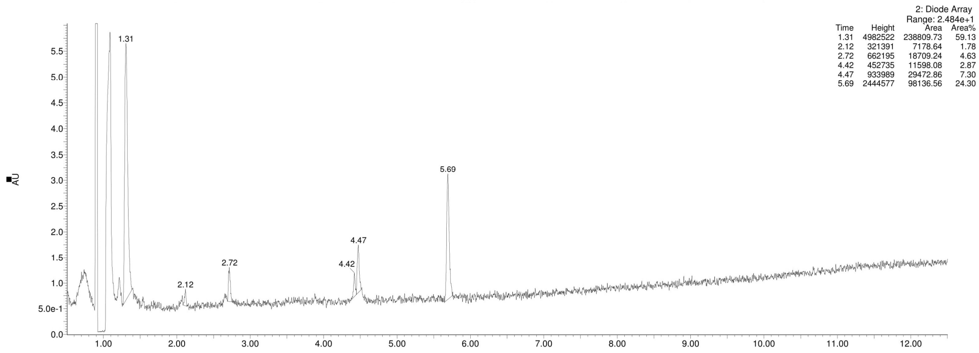

Time	Height	Area	Area%
1.31	4982522	238809.73	59.13
2.12	321391	7178.64	1.78
2.72	662195	18709.24	4.63
4.42	452735	11598.08	2.87
4.47	933989	29472.86	7.30
5.69	2444577	98136.56	24.30

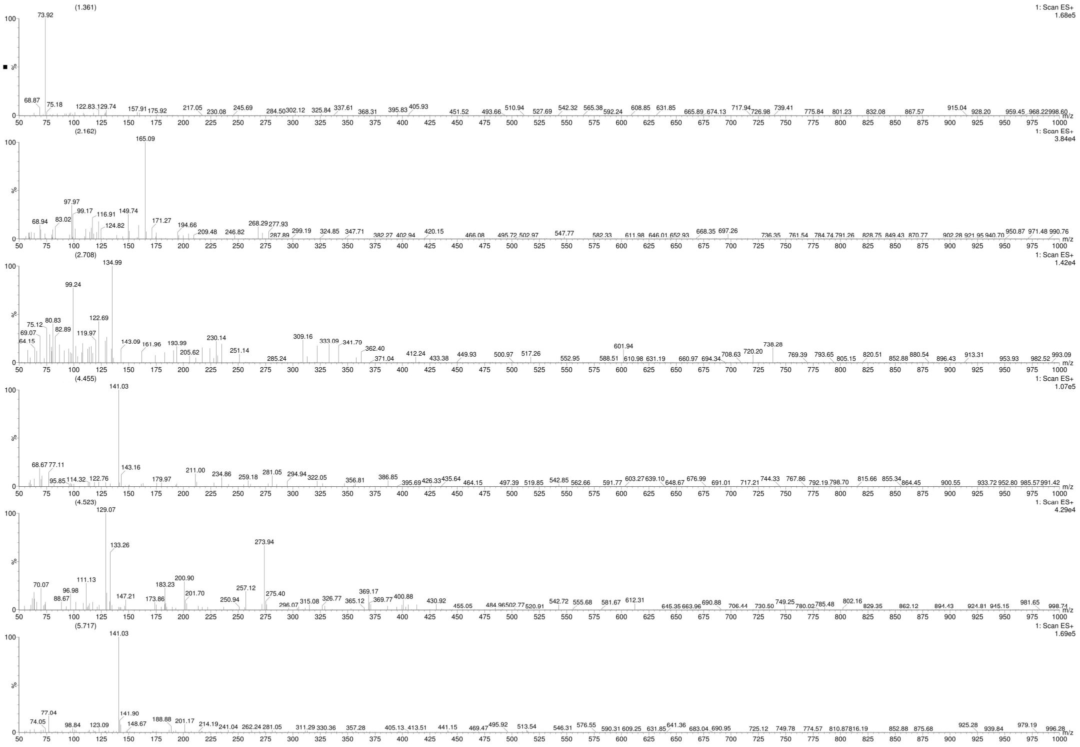

Entry 6:

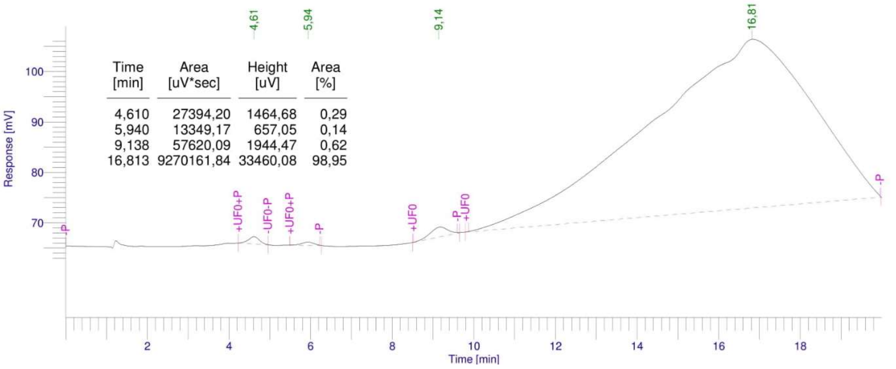

Time [min]	Area [uV*sec]	Height [uV]	Area [%]
4,610	27394,20	1464,68	0,29
5,940	13349,17	657,05	0,14
9,138	57620,09	1944,47	0,62
16,813	9270161,84	33460,08	98,95

Entry 7:

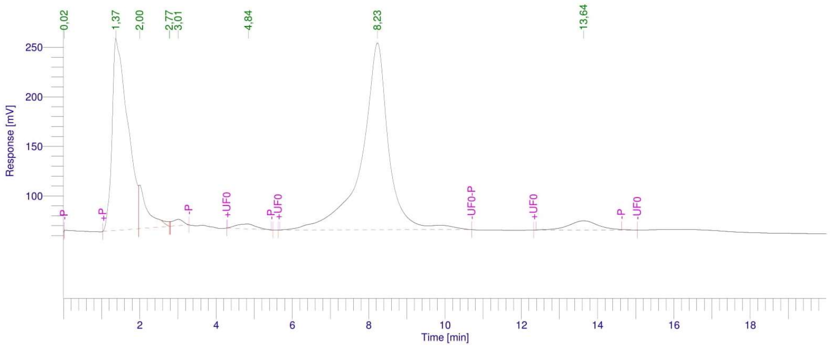

Peak #	Component Name	Time [min]	Area [uV*sec]	Height [uV]	Area [%]
1		0,017	298,36	1152,54	0,00
2		1,372	5451345,44	193746,24	33,43
3		1,998	726800,07	43944,16	4,46
4		2,775	32177,86	4700,72	0,20
5		2,791	4550,27	4760,80	0,03
6		3,007	127801,68	6499,12	0,78
7		4,845	176970,46	5020,15	1,09
8		8,230	9291758,70	188639,91	56,98
9		13,635	496638,69	9339,39	3,05

Entry 8:

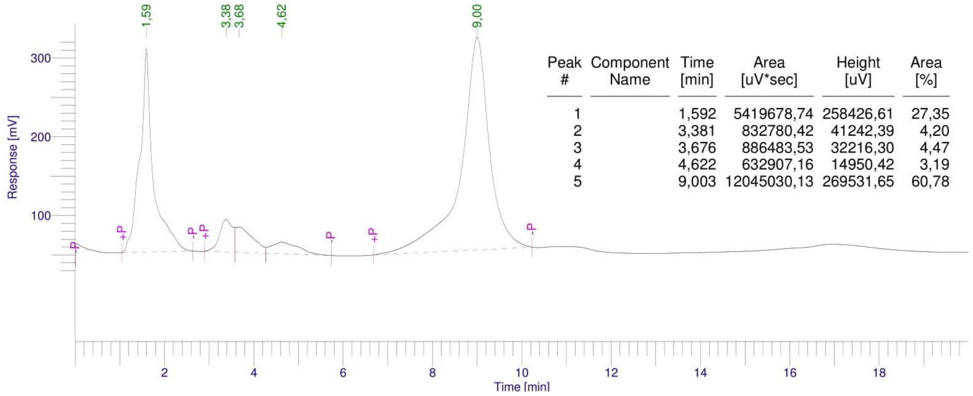

Peak #	Component Name	Time [min]	Area [uV*sec]	Height [uV]	Area [%]
1		1,592	5419678,74	258426,61	27,35
2		3,381	832780,42	41242,39	4,20
3		3,676	886483,53	32216,30	4,47
4		4,622	632907,16	14950,42	3,19
5		9,003	12045030,13	269531,65	60,78

Entry 9:

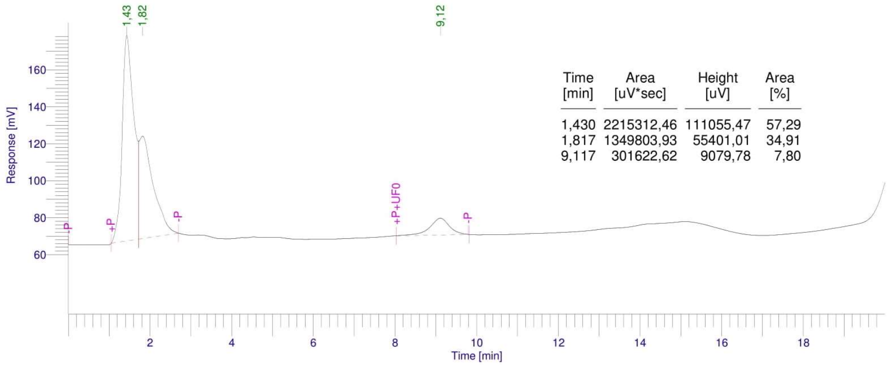

Time [min]	Area [uV*sec]	Height [uV]	Area [%]
1,430	2215312,46	111055,47	57,29
1,817	1349803,93	55401,01	34,91
9,117	301622,62	9079,78	7,80

Entry 10:

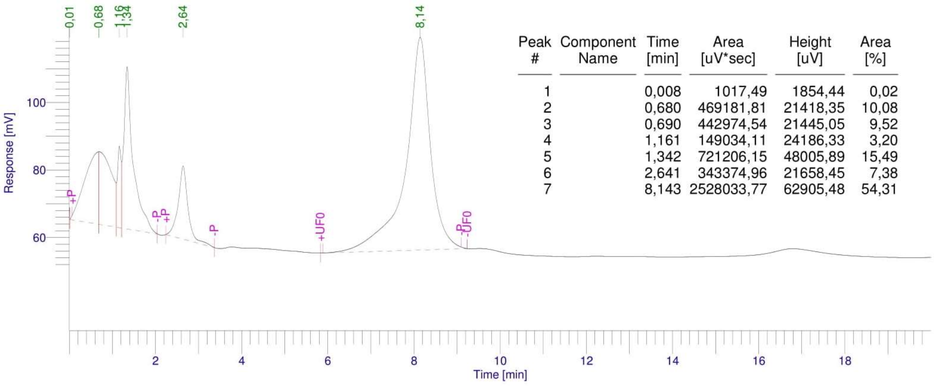

Peak #	Component Name	Time [min]	Area [uV*sec]	Height [uV]	Area [%]
1		0,008	1017,49	1854,44	0,02
2		0,680	469181,81	21418,35	10,08
3		0,690	442974,54	21445,05	9,52
4		1,161	149034,11	24186,33	3,20
5		1,342	721206,15	48005,89	15,49
6		2,641	343374,96	21658,45	7,38
7		8,143	2528033,77	62905,48	54,31

Entry 11:

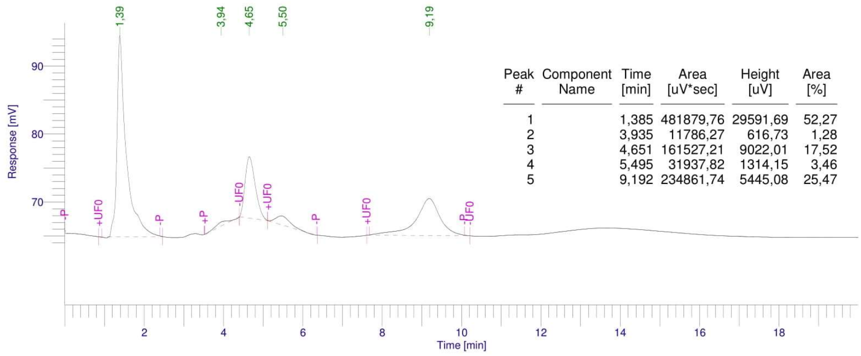

Peak #	Component Name	Time [min]	Area [uV*sec]	Height [uV]	Area [%]
1		1,385	481879,76	29591,69	52,27
2		3,935	11786,27	616,73	1,28
3		4,651	161527,21	9022,01	17,52
4		5,495	31937,82	1314,15	3,46
5		9,192	234861,74	5445,08	25,47

Entry 12:

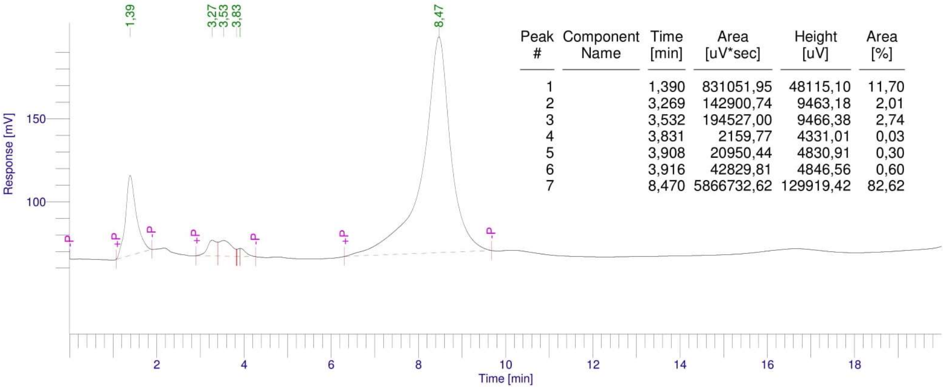

Peak #	Component Name	Time [min]	Area [uV*sec]	Height [uV]	Area [%]
1		1,390	831051,95	48115,10	11,70
2		3,269	142900,74	9463,18	2,01
3		3,532	194527,00	9466,38	2,74
4		3,831	2159,77	4331,01	0,03
5		3,908	20950,44	4830,91	0,30
6		3,916	42829,81	4846,56	0,60
7		8,470	5866732,62	129919,42	82,62

Entry 13:

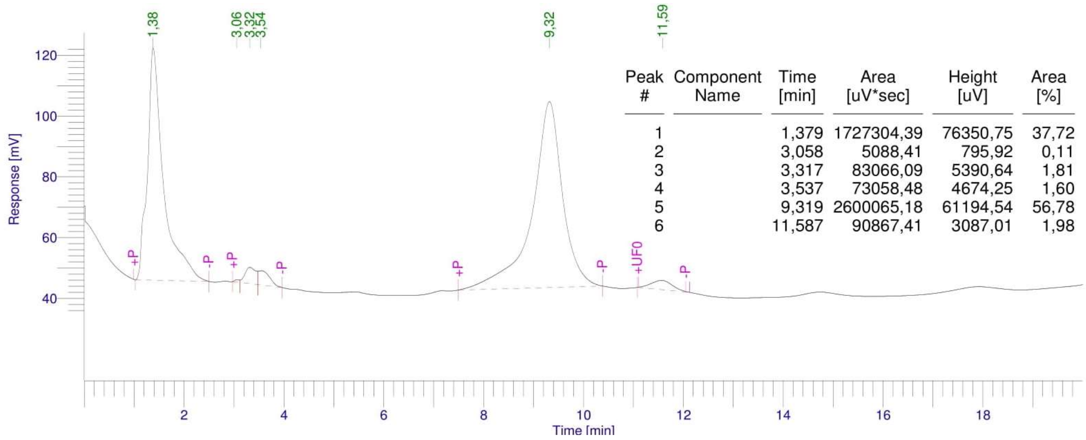

Peak #	Component Name	Time [min]	Area [uV*sec]	Height [uV]	Area [%]
1		1,379	1727304,39	76350,75	37,72
2		3,058	5088,41	795,92	0,11
3		3,317	83066,09	5390,64	1,81
4		3,537	73058,48	4674,25	1,60
5		9,319	2600065,18	61194,54	56,78
6		11,587	90867,41	3087,01	1,98

Entry 14:

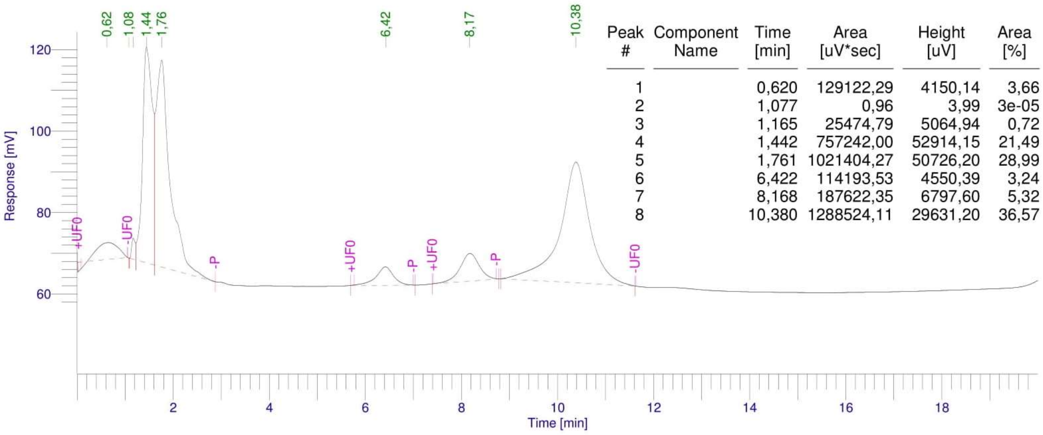

Peak #	Component Name	Time [min]	Area [uV*sec]	Height [uV]	Area [%]
1		0,620	129122,29	4150,14	3,66
2		1,077	0,96	3,99	3e-05
3		1,165	25474,79	5064,94	0,72
4		1,442	757242,00	52914,15	21,49
5		1,761	1021404,27	50726,20	28,99
6		6,422	114193,53	4550,39	3,24
7		8,168	187622,35	6797,60	5,32
8		10,380	1288524,11	29631,20	36,57

Entry 15:

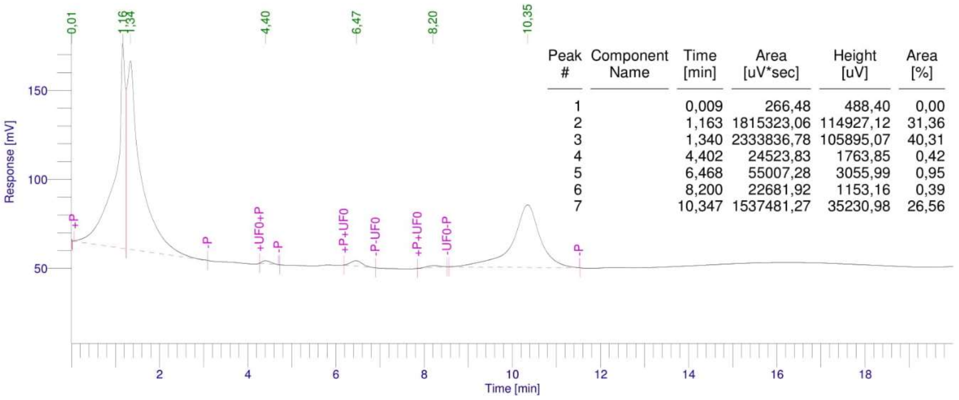

Peak #	Component Name	Time [min]	Area [uV*sec]	Height [uV]	Area [%]
1		0,009	266,48	488,40	0,00
2		1,163	1815323,06	114927,12	31,36
3		1,340	2333836,78	105895,07	40,31
4		4,402	24523,83	1763,85	0,42
5		6,468	55007,28	3055,99	0,95
6		8,200	22681,92	1153,16	0,39
7		10,347	1537481,27	35230,98	26,56

Entry 16:

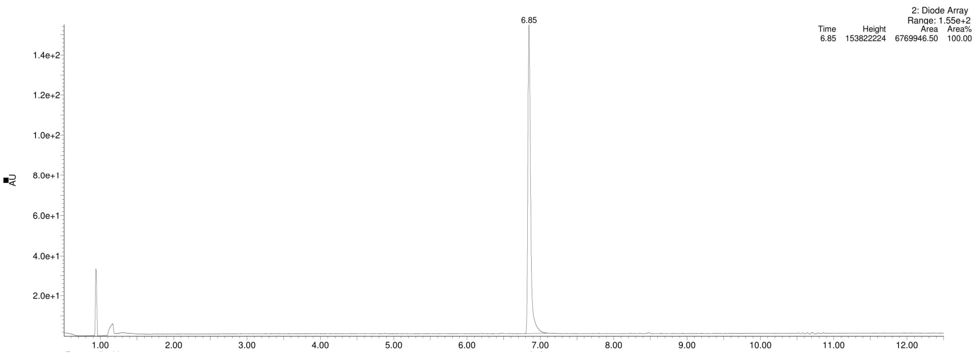

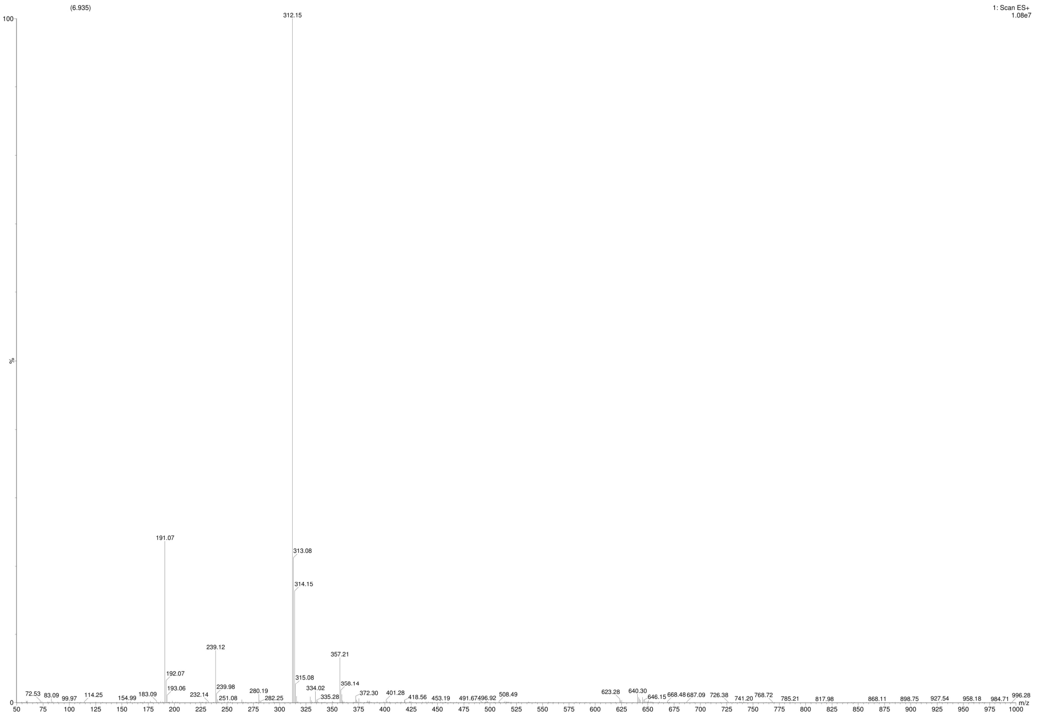

Entry 17:

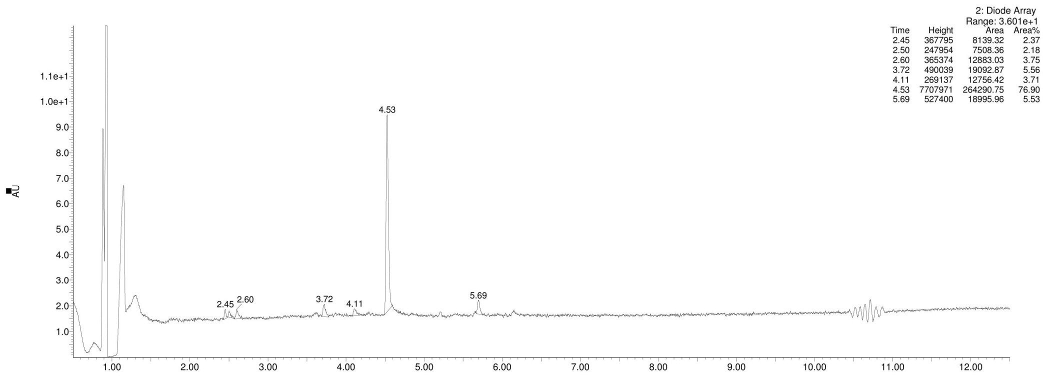

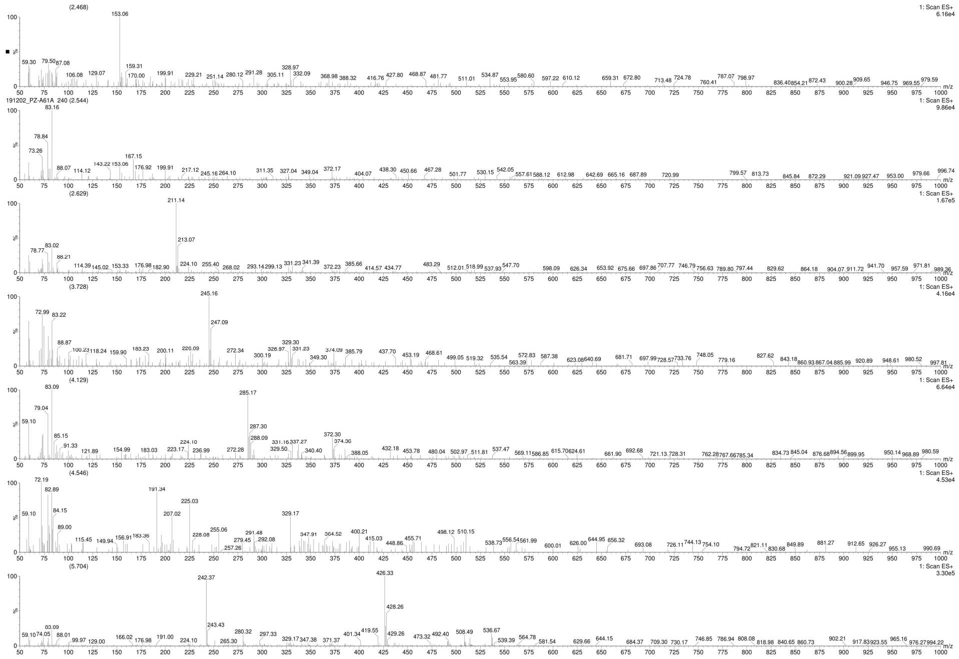

Entry 18:

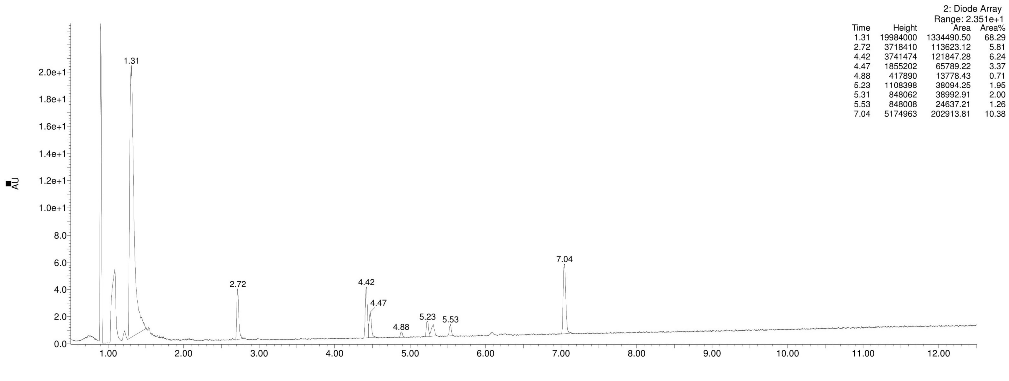

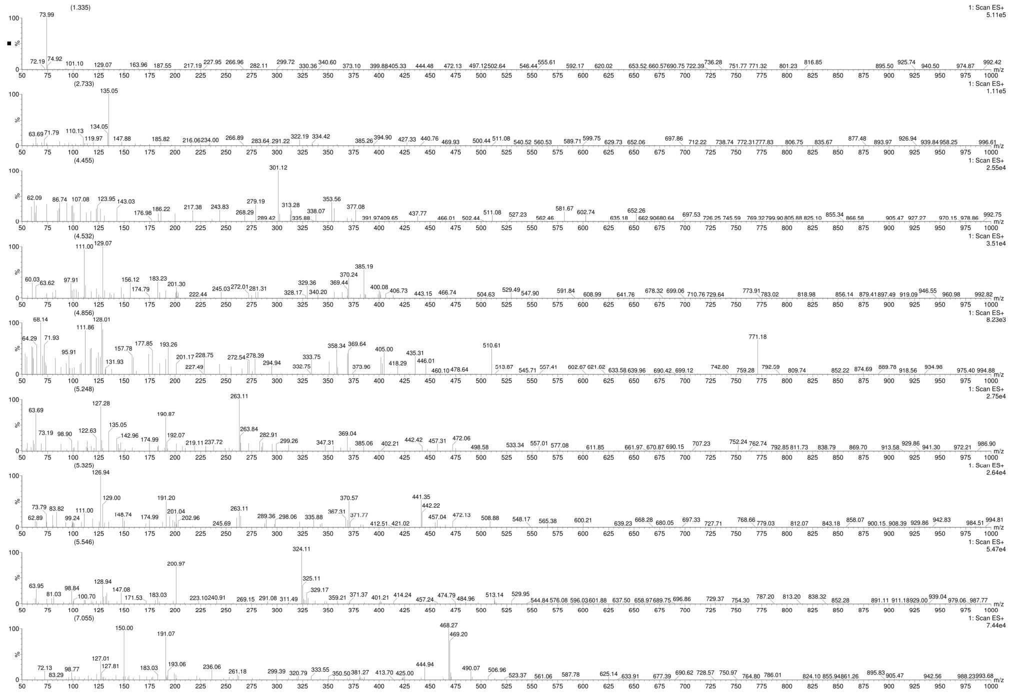

Entry 19:

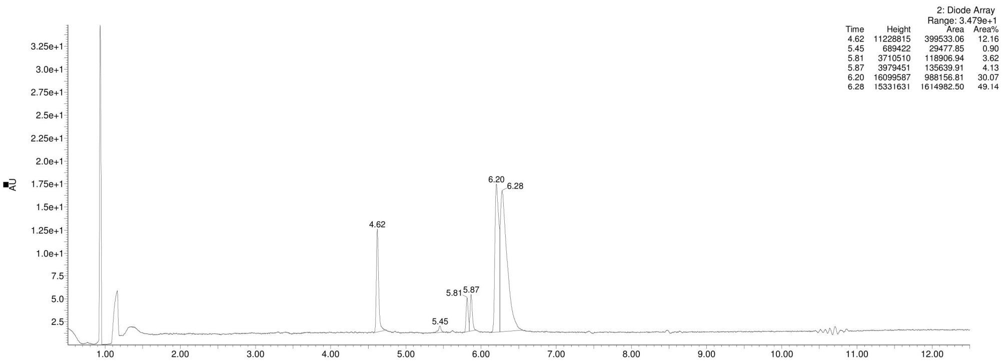

2: Diode Array
Range: 3.479e+1
Time Height Area Area%
4.62 11228815 399533.06 12.16
5.45 689422 29477.85 0.90
5.81 3710510 118906.94 3.62
5.87 3979451 135639.91 4.13
6.20 16099587 988156.81 30.07
6.28 15331631 1614982.50 49.14

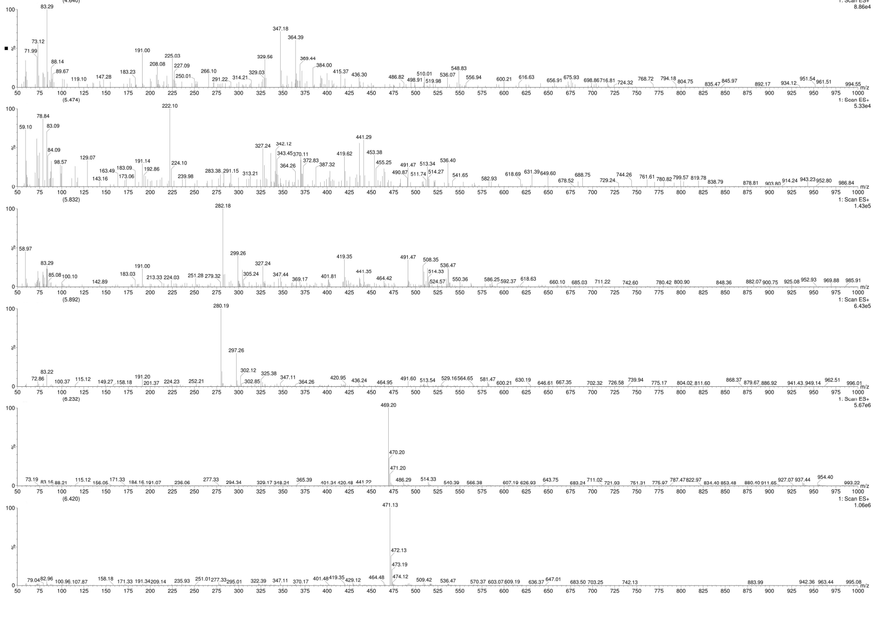

Entry 20:

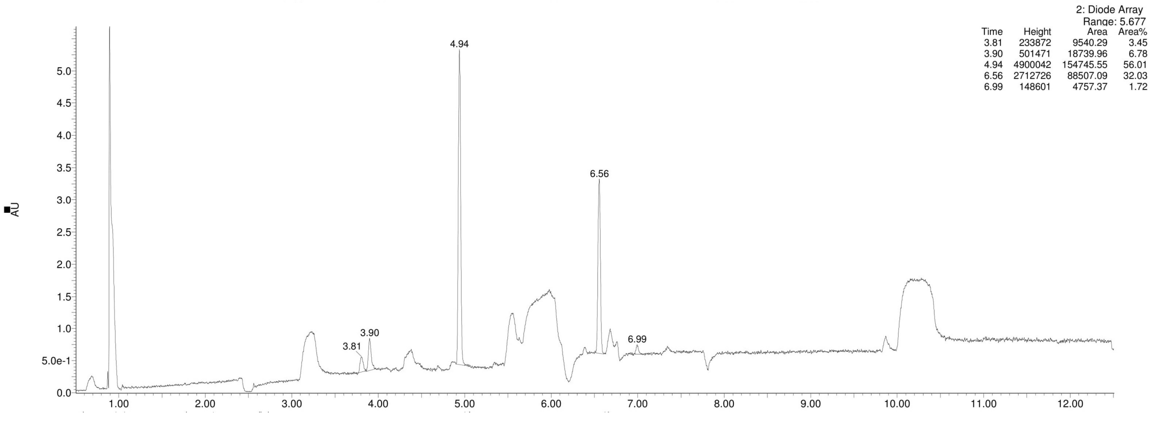

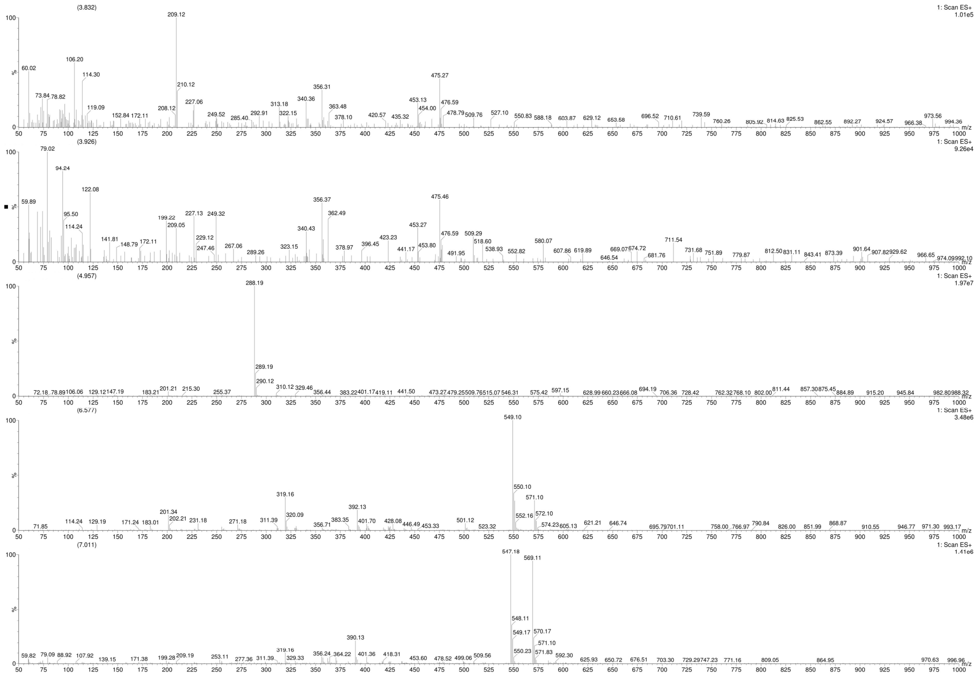

Entry 23:

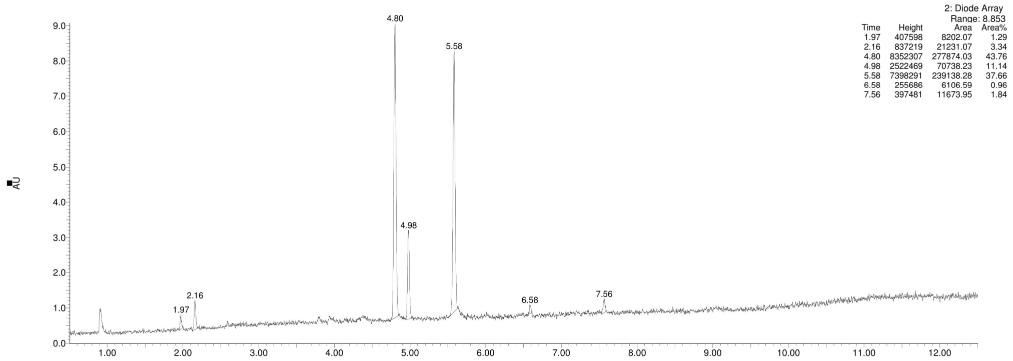

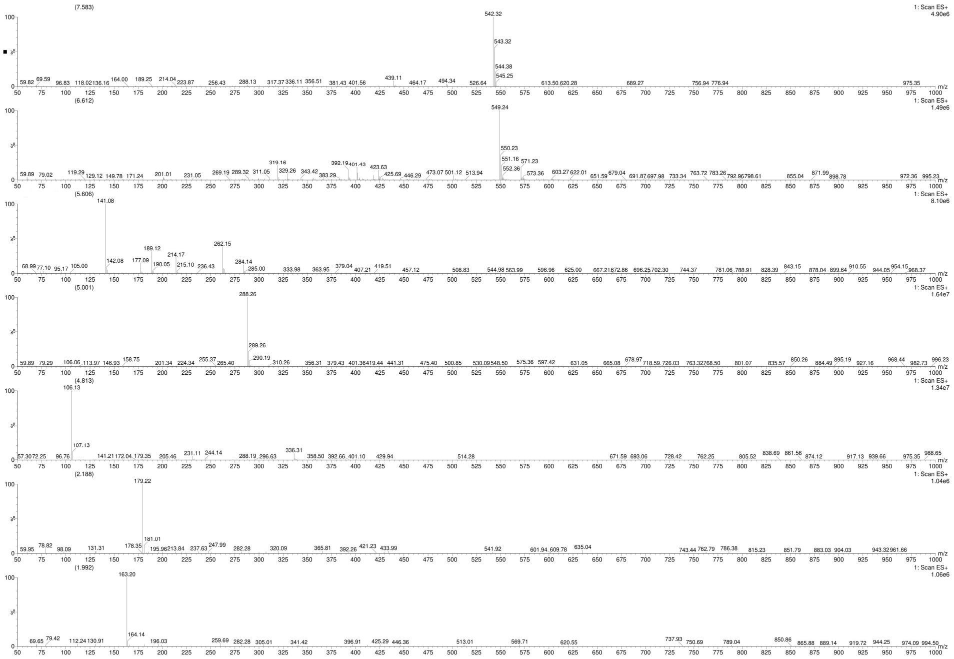
(7.583) 1: Scan ES+ 4.90e6 542.32
(6.612) 1: Scan ES+ 1.49e6 549.24
(5.606) 1: Scan ES+ 8.10e6 141.08
(5.001) 1: Scan ES+ 1.64e7 288.26
(4.813) 1: Scan ES+ 1.34e7 106.13
(2.188) 1: Scan ES+ 1.04e6 179.22
(1.992) 1: Scan ES+ 1.06e6 163.20
m/z

Entry 24:

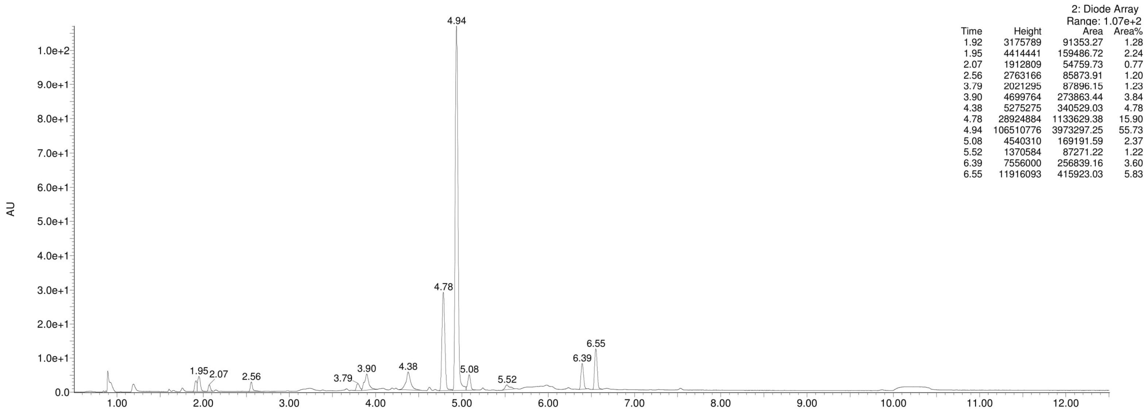
2: Diode Array
Range: 1.07e+2
Time Height Area Area%
1.92 3175789 91353.27 1.28
1.95 4414441 159486.72 2.24
2.07 1912809 54759.73 0.77
2.56 2763166 85873.91 1.20
3.79 2021295 87896.15 1.23
3.90 4699764 273863.44 3.84
4.38 5275275 340529.03 4.78
4.78 28924884 1133629.38 15.90
4.94 106510776 3973297.25 55.73
5.08 4540310 169191.59 2.37
5.52 1370584 87271.22 1.22
6.39 7556000 256839.16 3.60
6.55 11916093 415923.03 5.83
AU

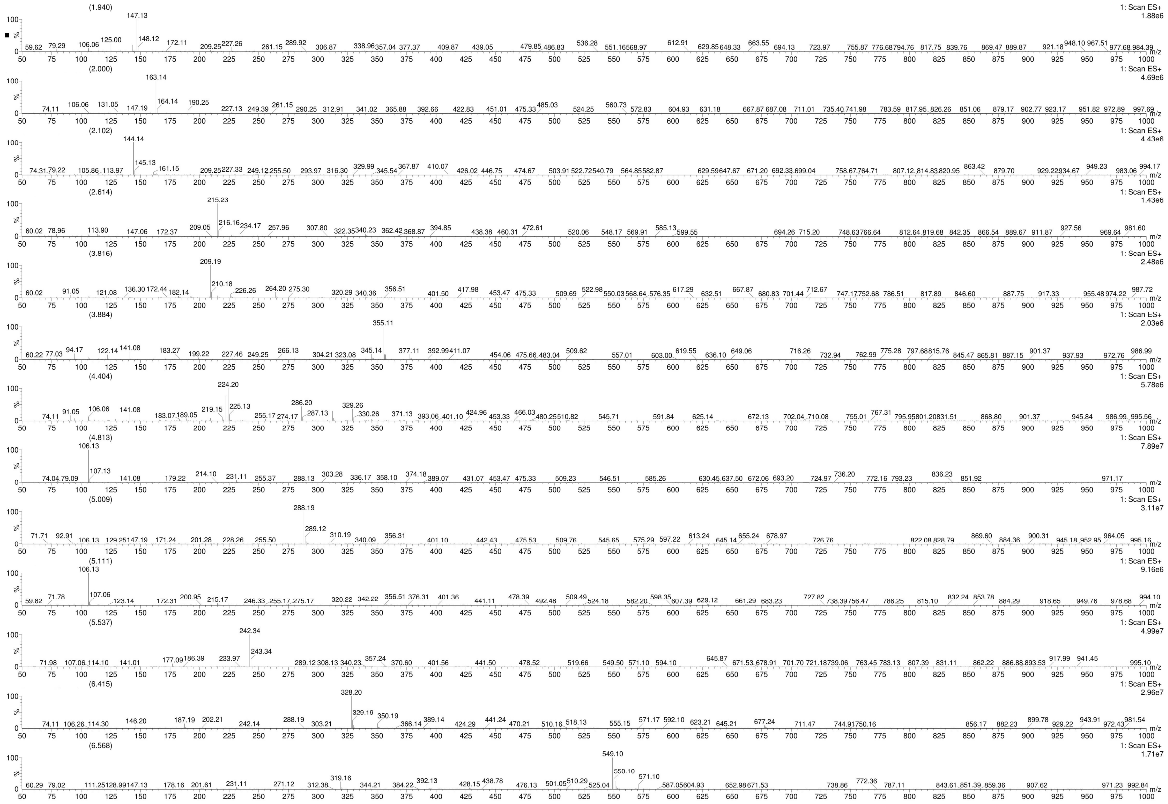

Entry 25:

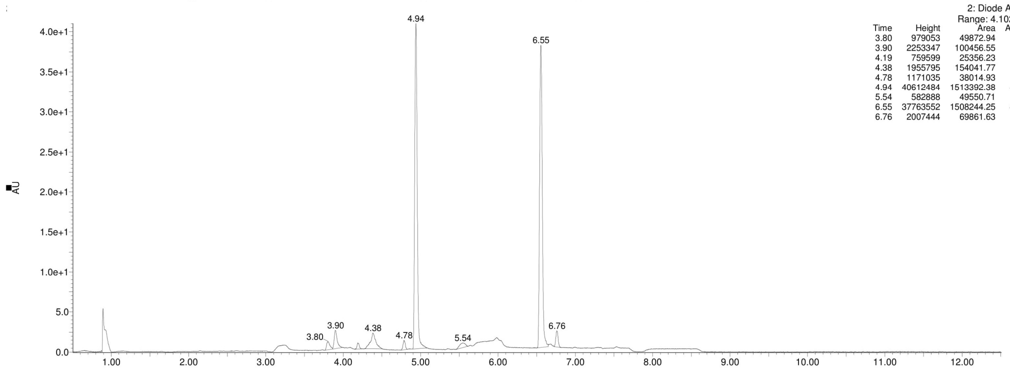

Time	Height	Area	Area%
3.80	979053	49872.94	1.42
3.90	2253347	100456.55	2.86
4.19	759599	25356.23	0.72
4.38	1955795	154041.77	4.39
4.78	1171035	38014.93	1.08
4.94	40612484	1513392.38	43.13
5.54	582888	49550.71	1.41
6.55	37763552	1508244.25	42.98
6.76	2007444	69861.63	1.99

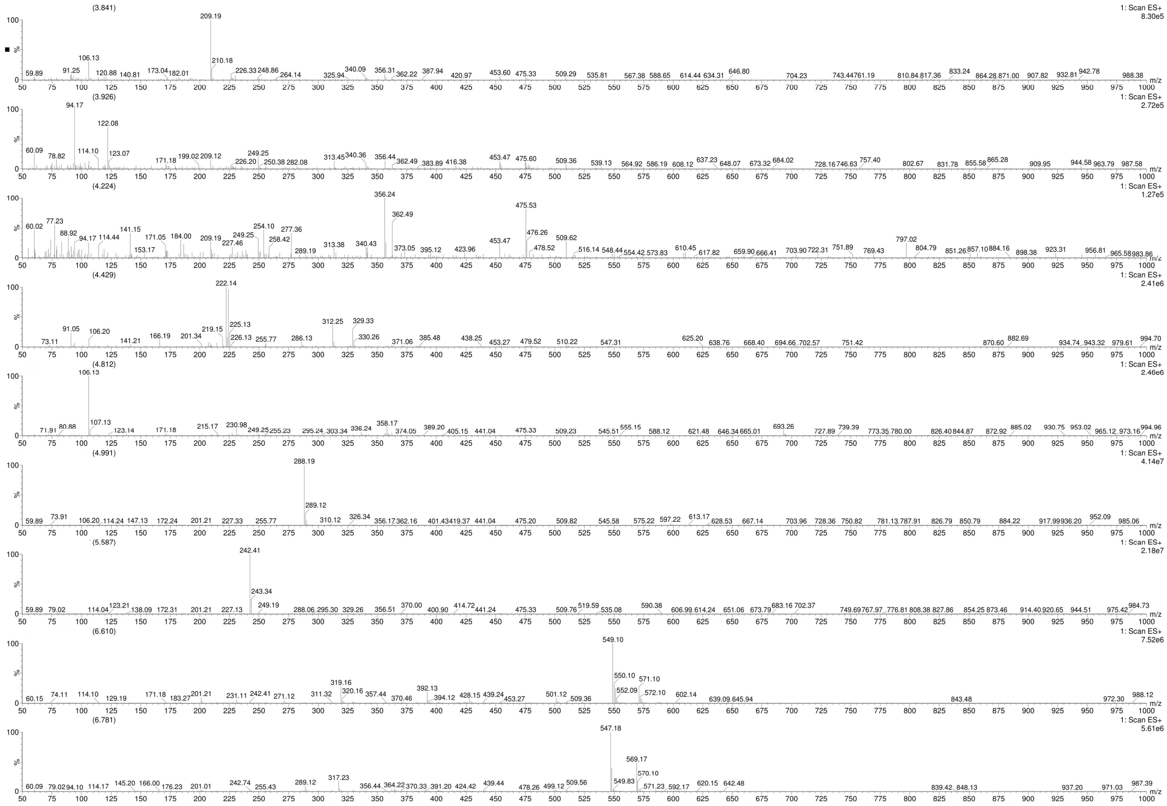
(3.841) 1: Scan ES+ 8.30e5
209.19
(3.926) 1: Scan ES+ 2.72e5
94.17
(4.224) 1: Scan ES+ 1.27e5
356.24
(4.429) 1: Scan ES+ 2.41e6
222.14
(4.812) 1: Scan ES+ 2.46e6
106.13
(4.991) 1: Scan ES+ 4.14e7
288.19
(5.587) 1: Scan ES+ 2.18e7
242.41
(6.610) 1: Scan ES+ 7.52e6
549.10
(6.781) 1: Scan ES+ 5.61e6
547.18
m/z

Entry 26:

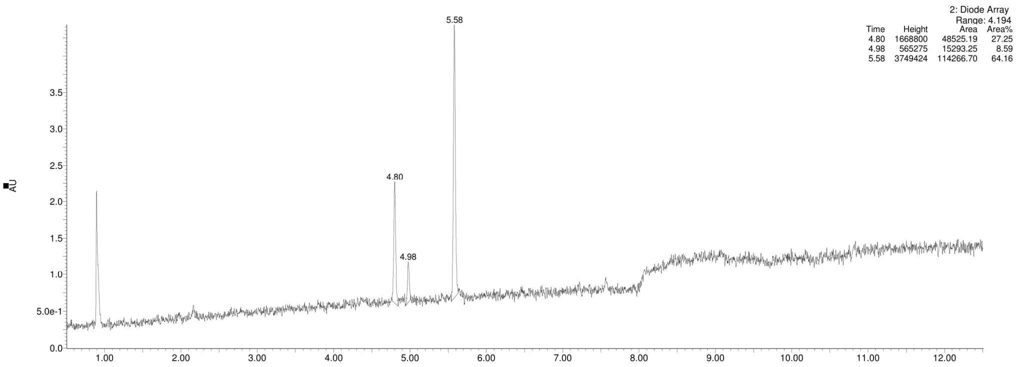
2: Diode Array
Range: 4.194
Time Height Area Area%
4.80 1668800 48525.19 27.25
4.98 565275 15293.25 8.59
5.58 3749424 114266.70 64.16
AU
4.80
4.98
5.58

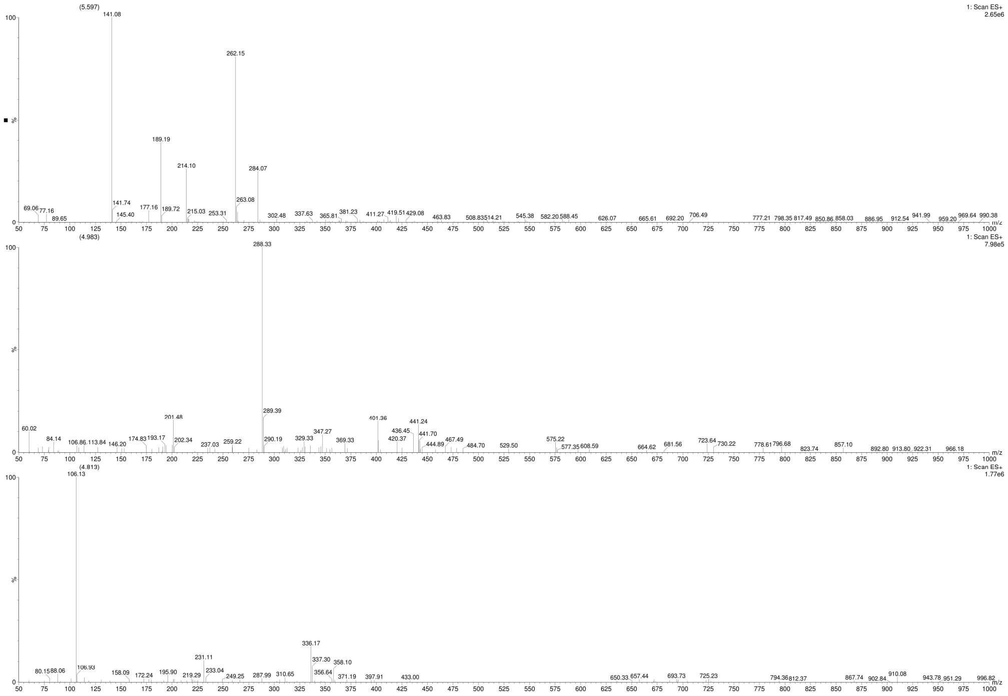

Entry 27:

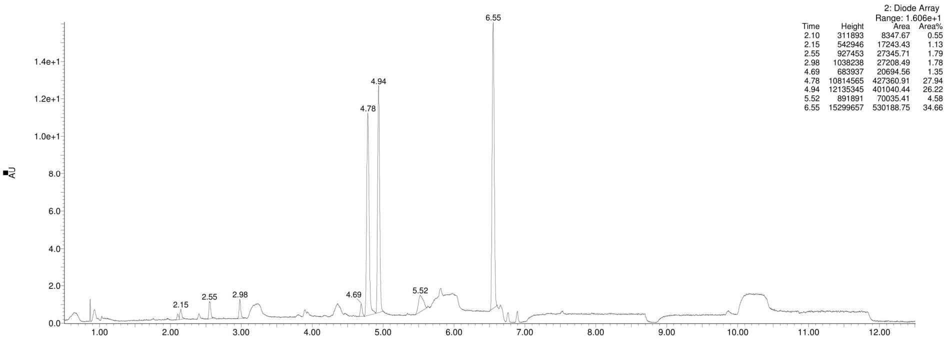
2: Diode Array
Range: 1.606e+1
Time Height Area Area%
2.10 311893 8347.67 0.55
2.15 542946 17243.43 1.13
2.55 927453 27345.71 1.79
2.98 1038238 27208.49 1.78
4.69 683937 20694.56 1.35
4.78 10814565 427360.91 27.94
4.94 12135345 401040.44 26.22
5.52 891891 70035.41 4.58
6.55 15299657 530188.75 34.66
AU

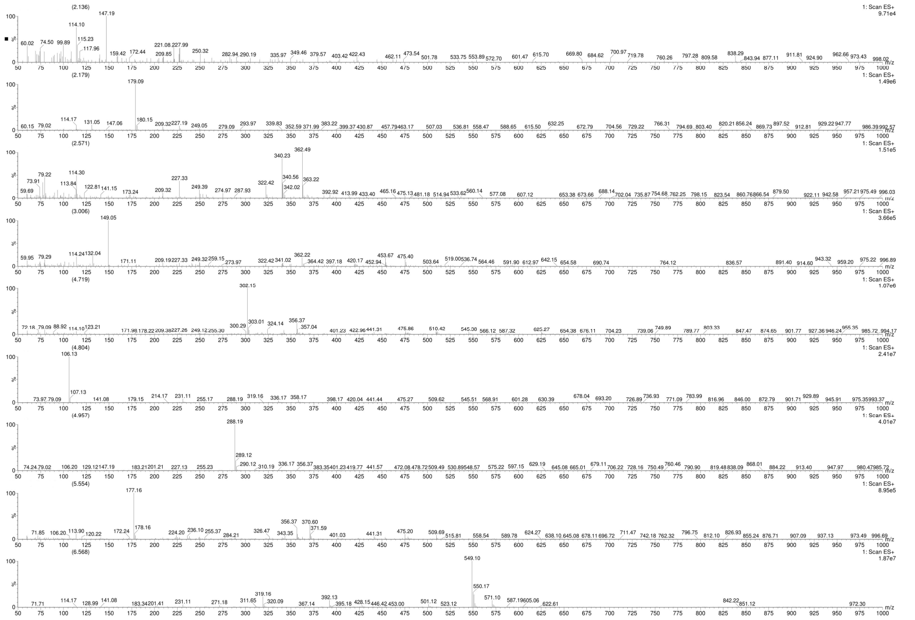

Entry 28:

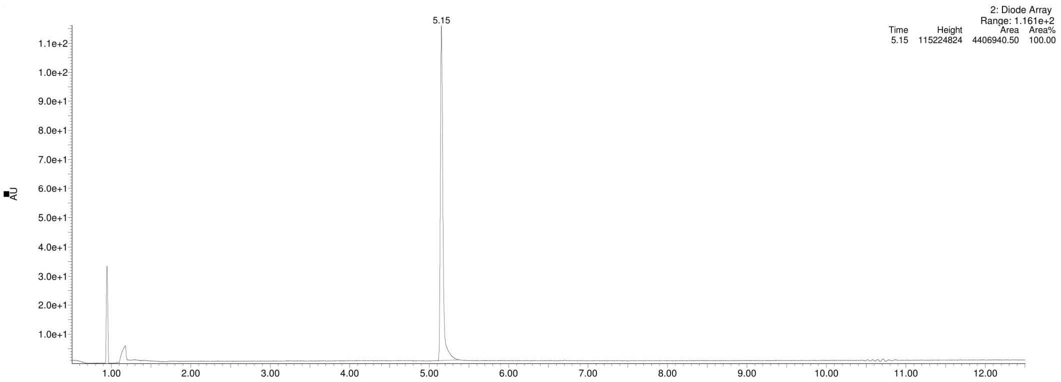

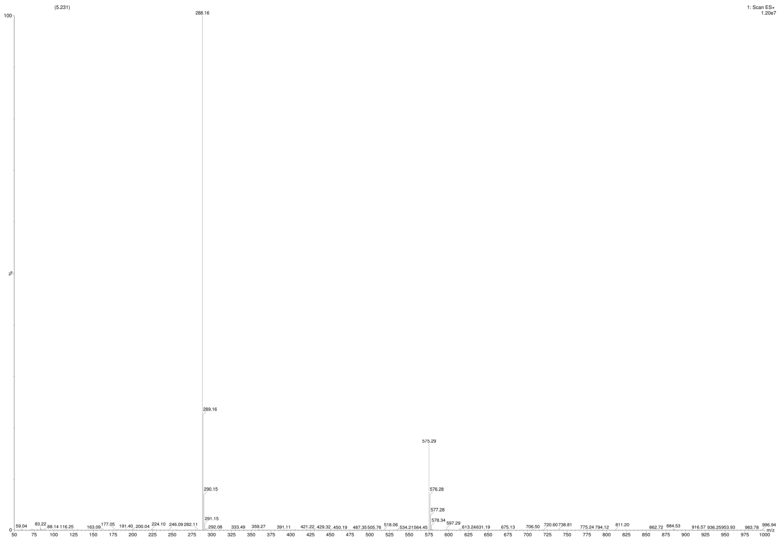

Entry 29:

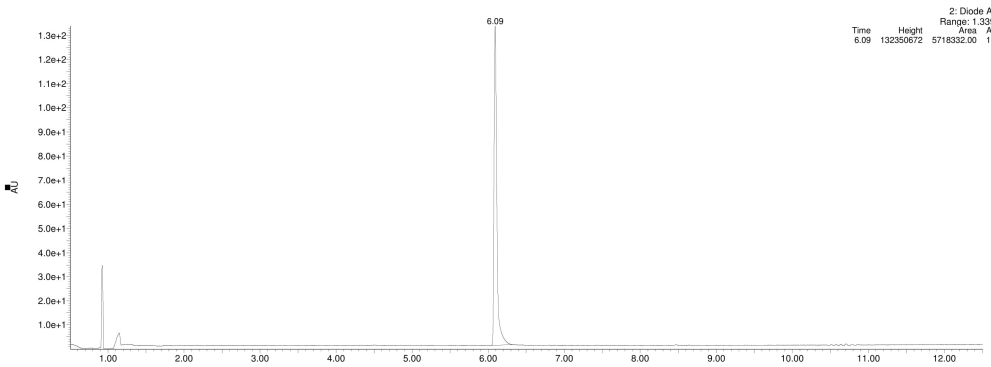

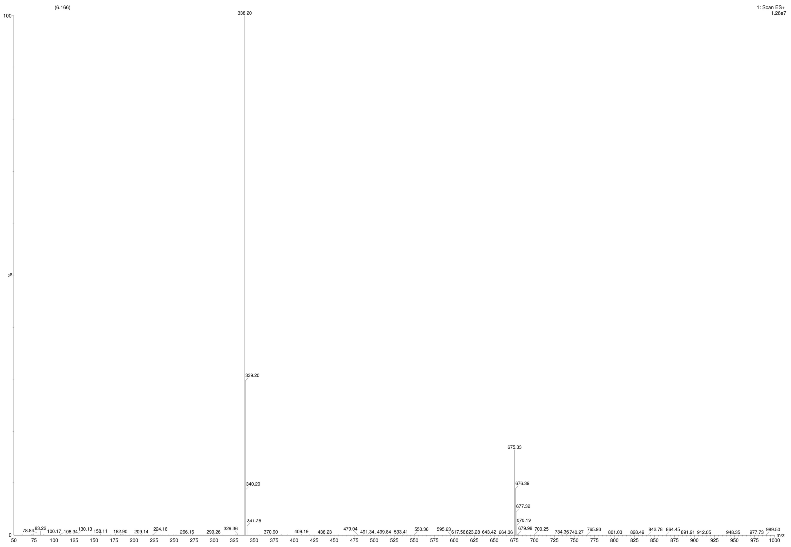

(6.166)
1: Scan ES+
1.26e7
338.20
339.20
340.20
675.33
676.39
677.32
m/z

Entry 30:

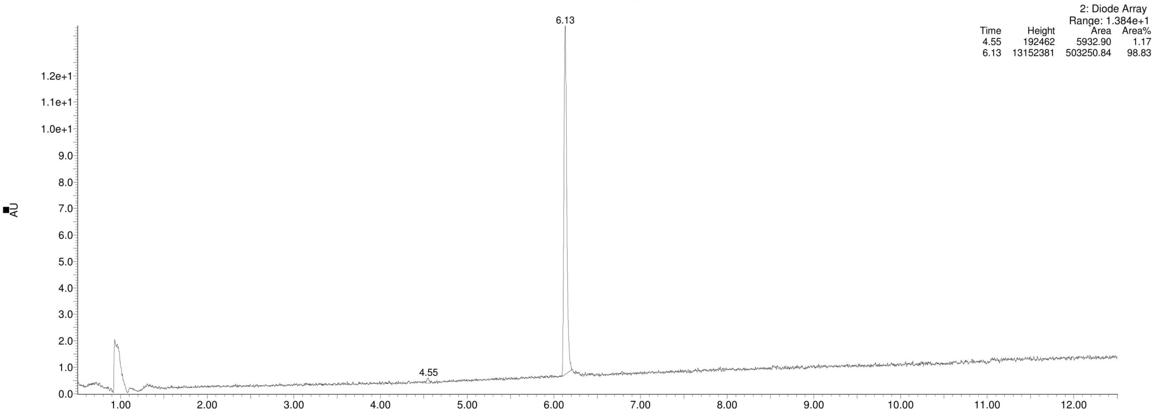

2: Diode Array
Range: 1.384e+1
Time Height Area Area%
4.55 192462 5932.90 1.17
6.13 13152381 503250.84 98.83
6.13
4.55
AU

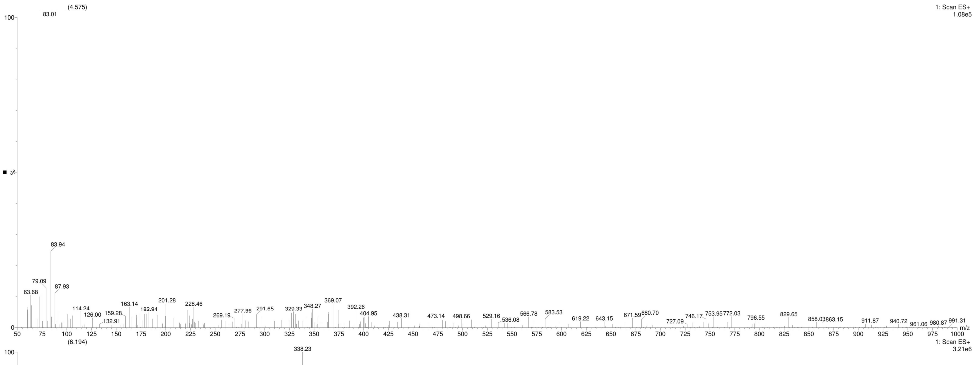

(4.575)
1: Scan ES+
1.08e5
83.01
83.94
m/z

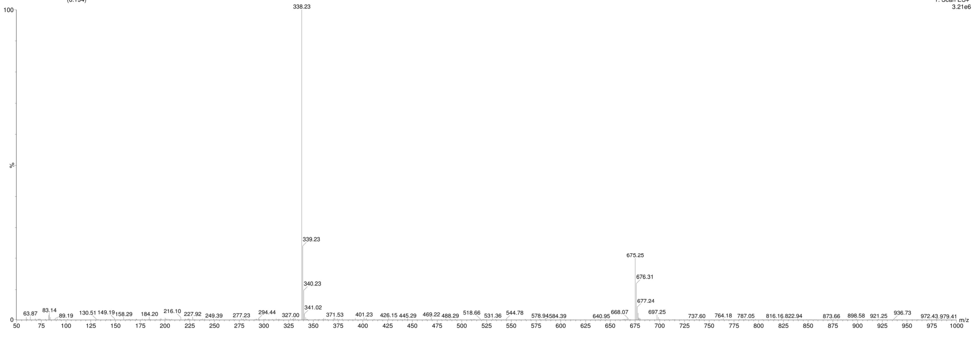

(6.194)
1: Scan ES+
3.21e6
338.23
339.23
340.23
675.25
676.31
677.24
m/z

Entry 31:

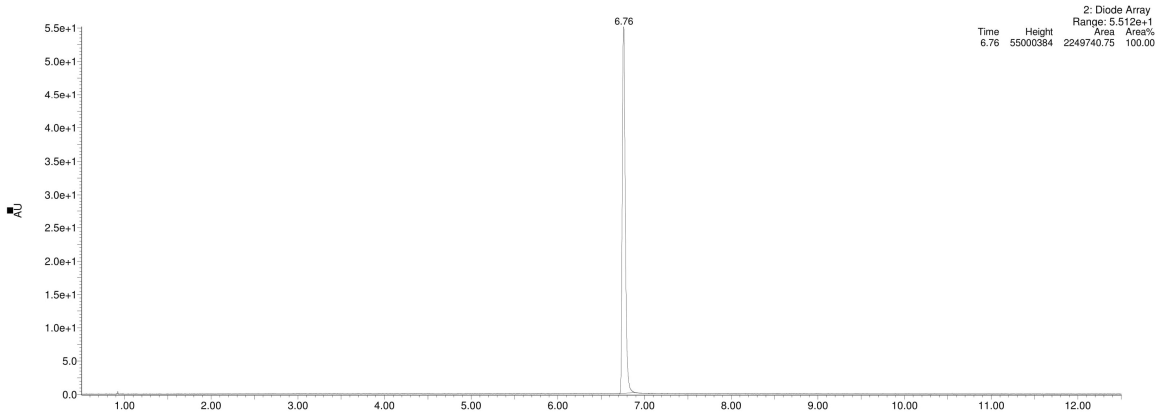
2: Diode Array
Range: 5.512e+1
Time Height Area Area%
6.76 55000384 2249740.75 100.00
6.76
AU

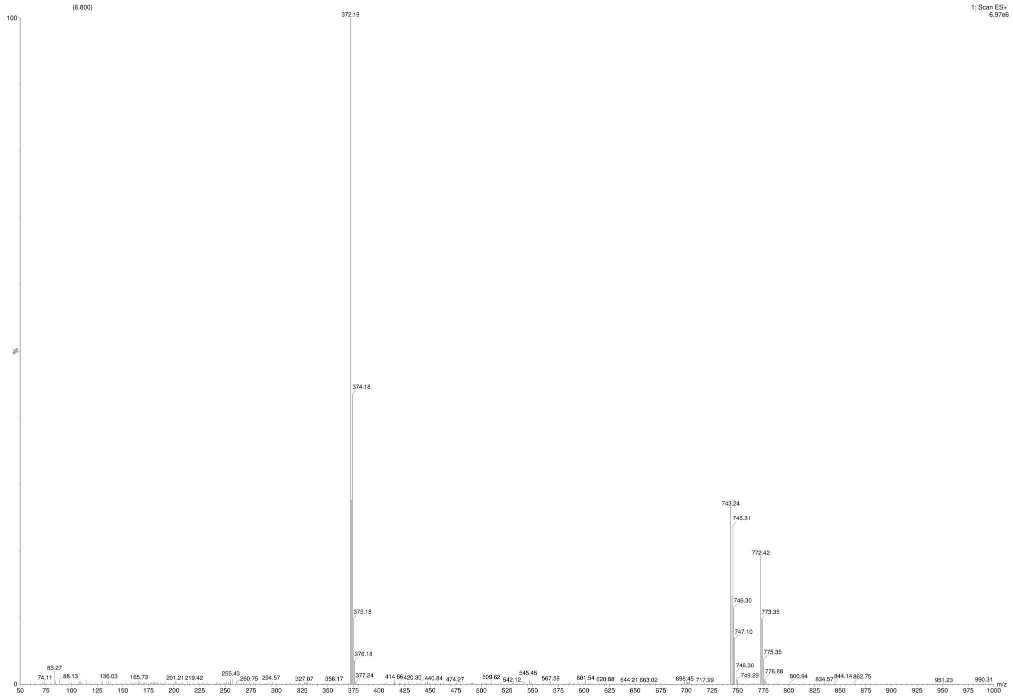
(6.800)
1: Scan ES+
6.97e6
372.19
374.18
743.24
772.42
%
m/z

4.3. Development the method C of synthesis (Table 5)

5d standard:

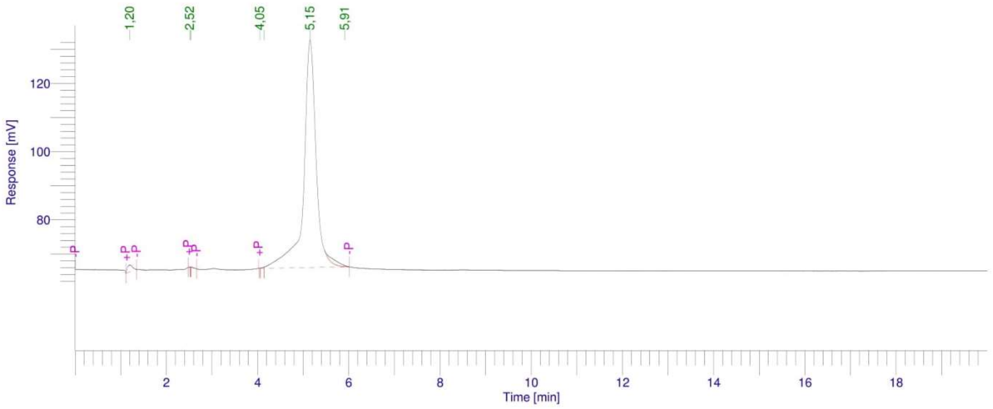

6a standard:

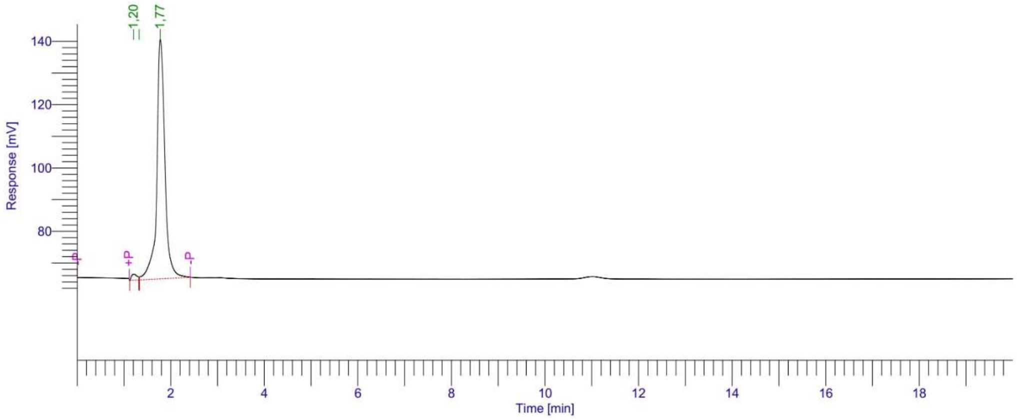

10a standard:

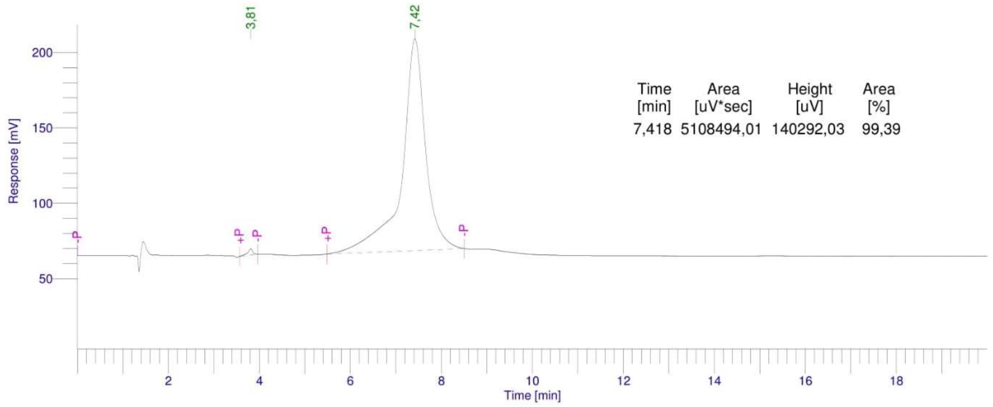

Entry 1:

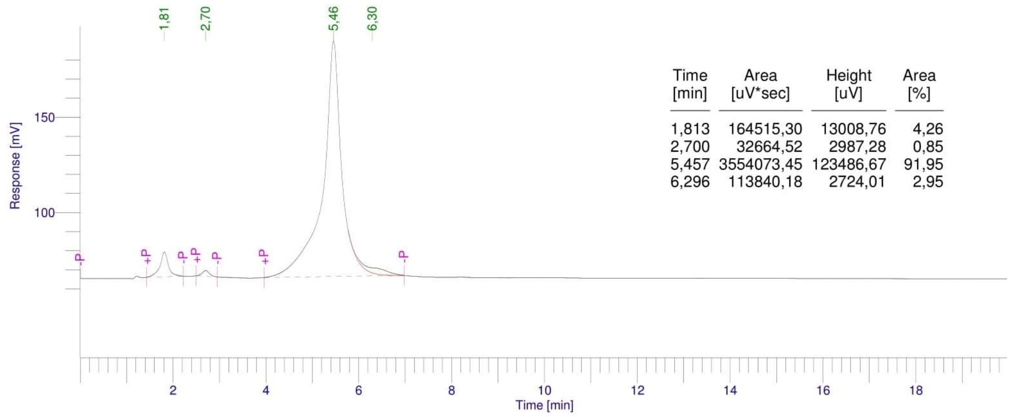

Entry 2:

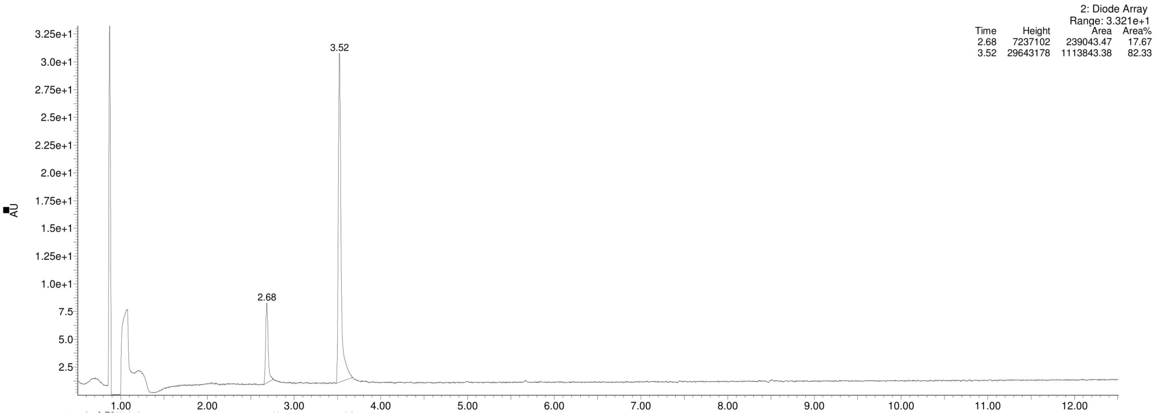

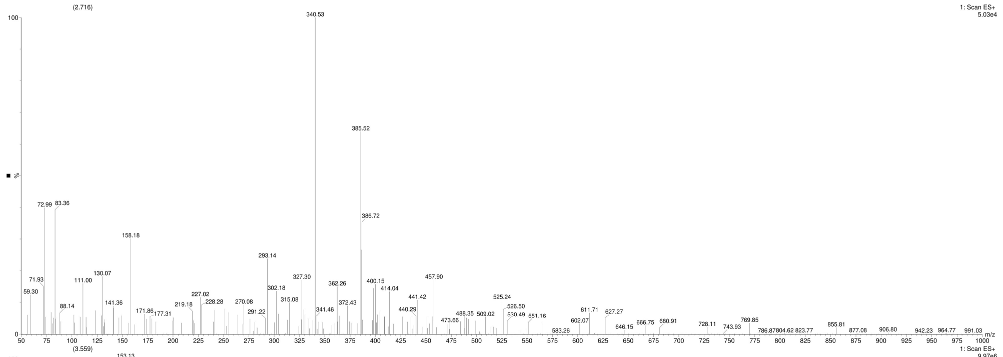

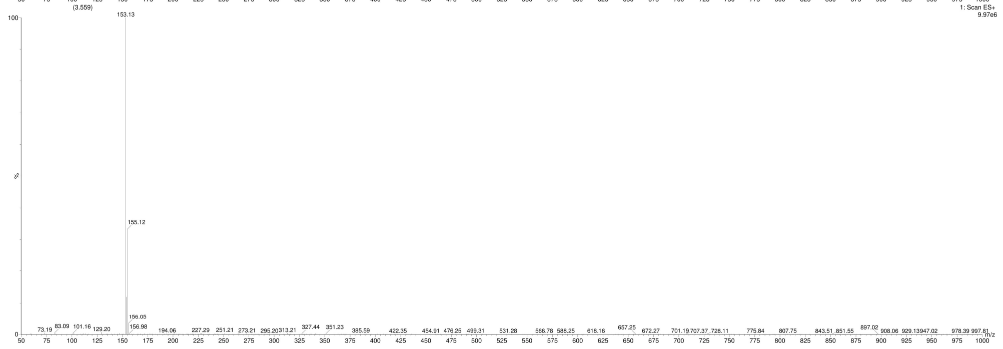

Entry 3:

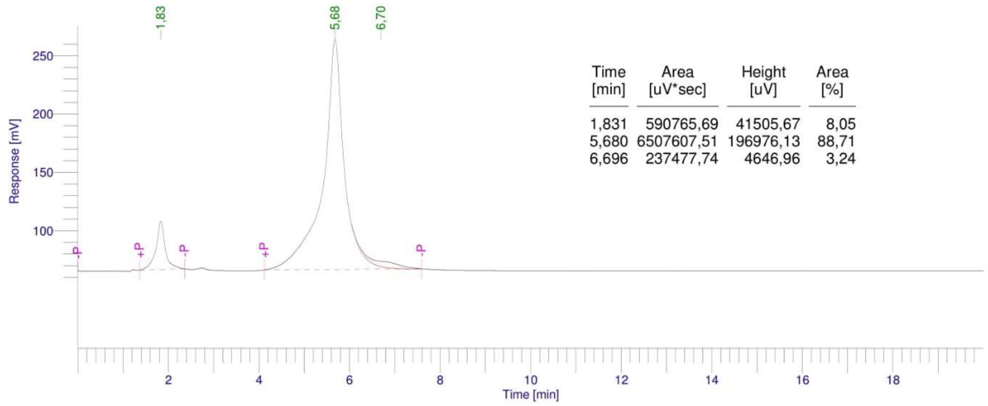

Entry 4:

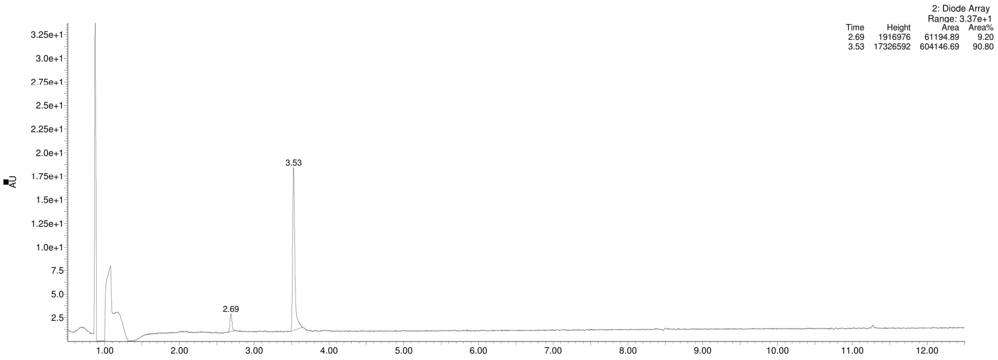

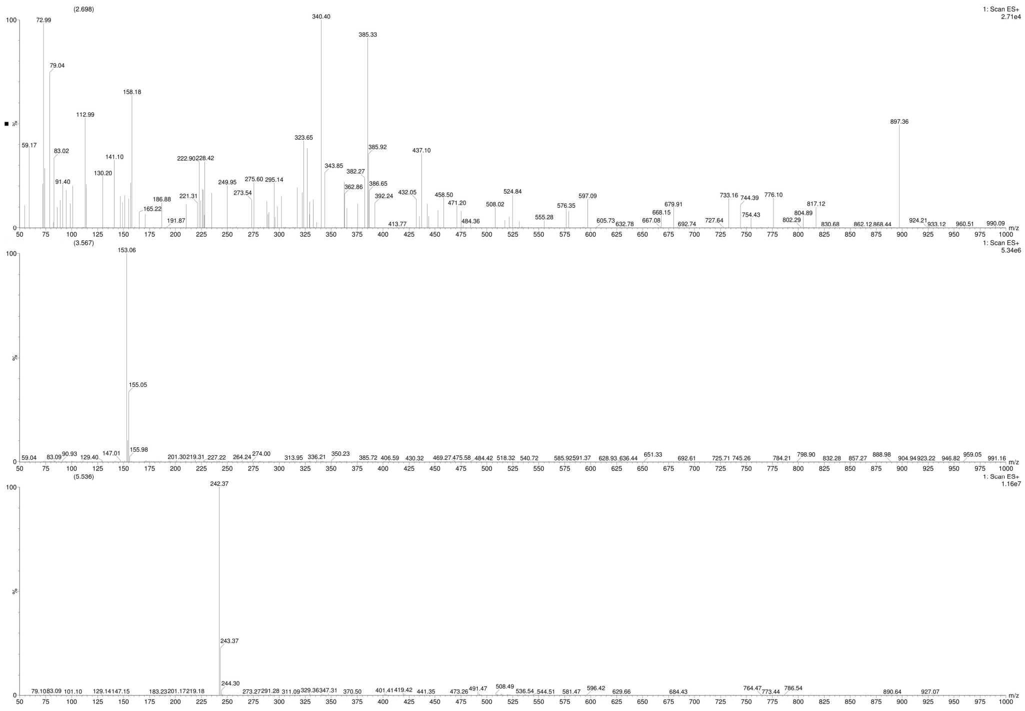

Entry 5:

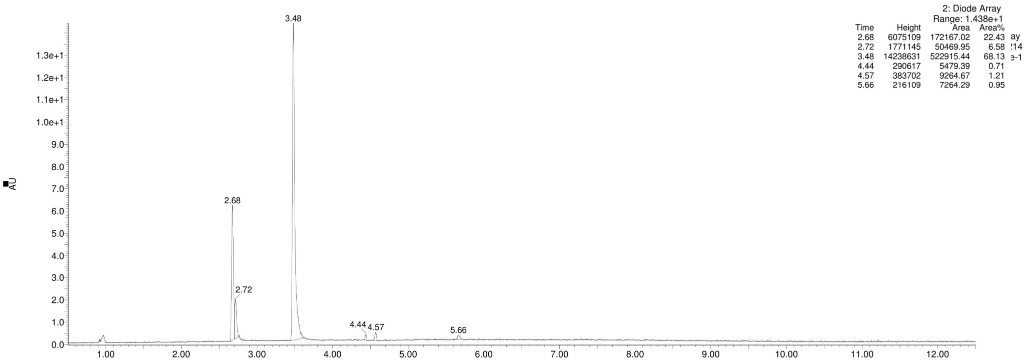
2: Diode Array
Range: 1.438e+1
Time Height Area Area%
2.68 6075109 172167.02 22.43
2.72 1771145 50469.95 6.58
3.48 14238631 522915.44 68.13
4.44 290617 5479.39 0.71
4.57 383702 9264.67 1.21
5.66 216109 7264.29 0.95
AU
3.48
2.68
2.72
4.44
4.57
5.66

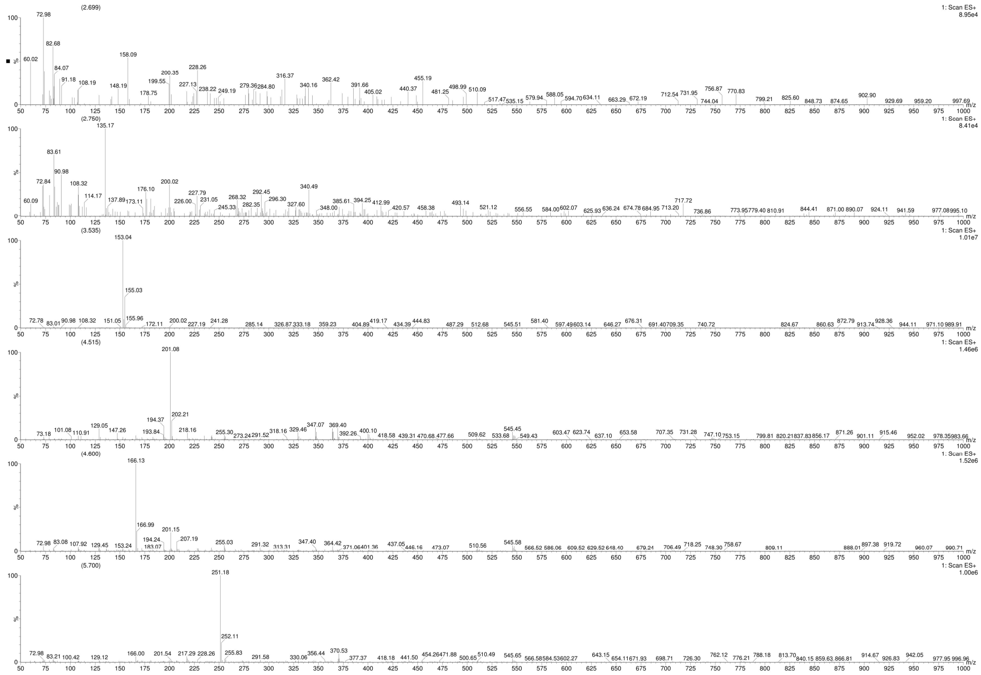
(2.699)
1: Scan ES+
8.95e4
(2.750)
1: Scan ES+
8.41e4
(3.535)
1: Scan ES+
1.01e7
(4.515)
1: Scan ES+
1.46e6
(4.600)
1: Scan ES+
1.52e6
(5.700)
1: Scan ES+
1.00e6
m/z

5a standard:

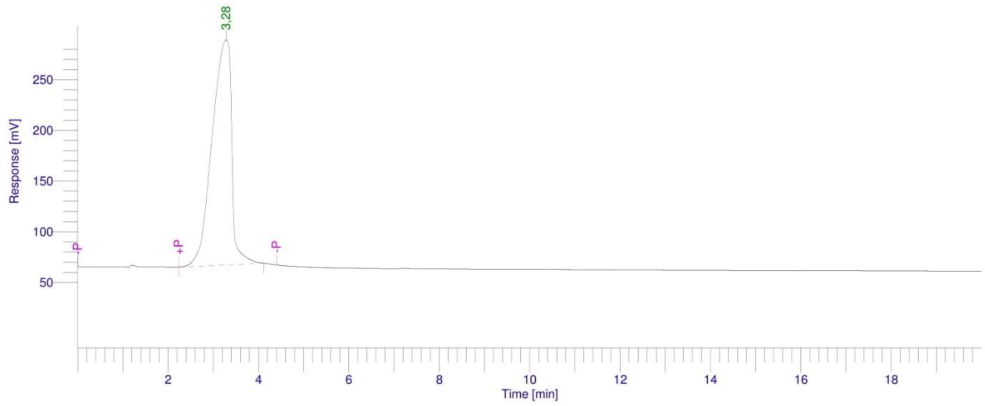
3.28
Response [mV]
Time [min]

Entry 6:

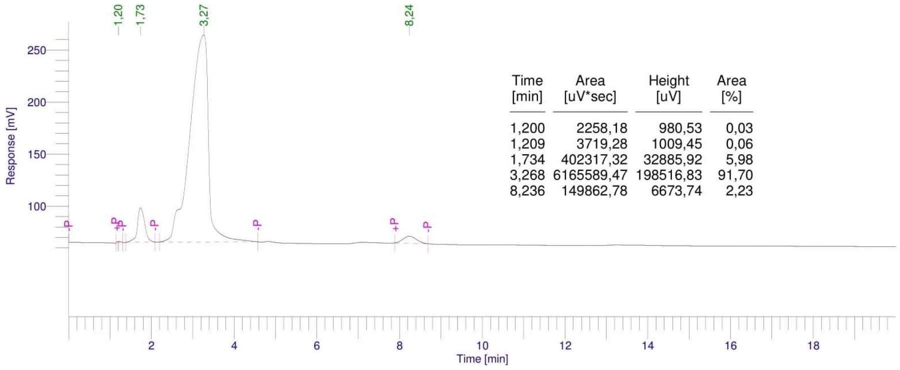

Time [min]	Area [uV*sec]	Height [uV]	Area [%]
1,200	2258,18	980,53	0,03
1,209	3719,28	1009,45	0,06
1,734	402317,32	32885,92	5,98
3,268	6165589,47	198516,83	91,70
8,236	149862,78	6673,74	2,23

Entry 7:

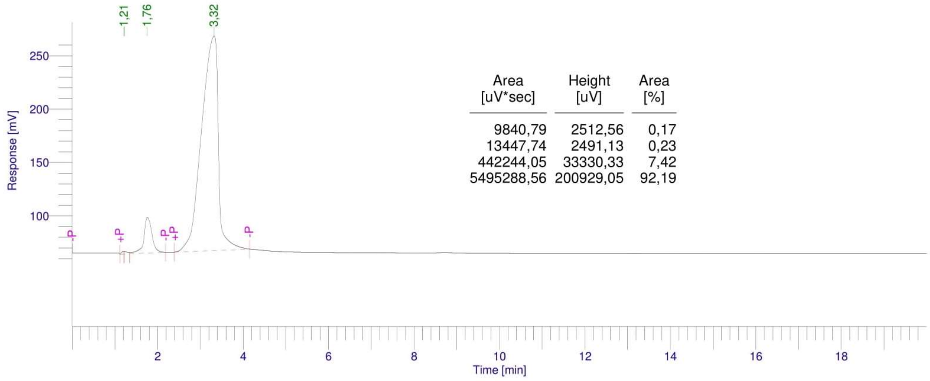

Area [uV*sec]	Height [uV]	Area [%]
9840,79	2512,56	0,17
13447,74	2491,13	0,23
442244,05	33330,33	7,42
5495288,56	200929,05	92,19

Entry 8:

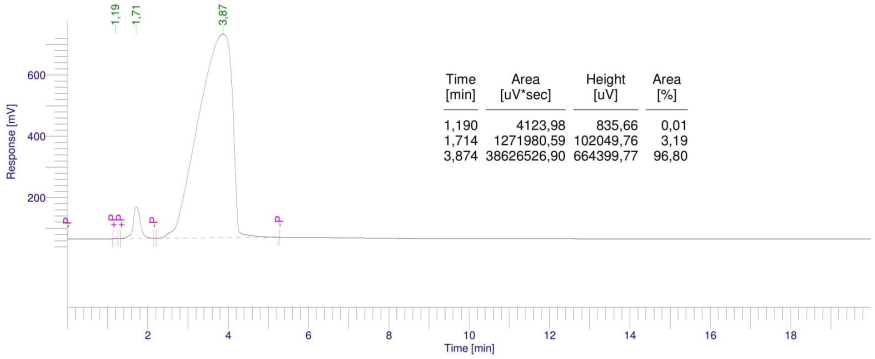

Time [min]	Area [uV*sec]	Height [uV]	Area [%]
1,190	4123,98	835,66	0,01
1,714	1271980,59	102049,76	3,19
3,874	38626526,90	664399,77	96,80

Entry 9:

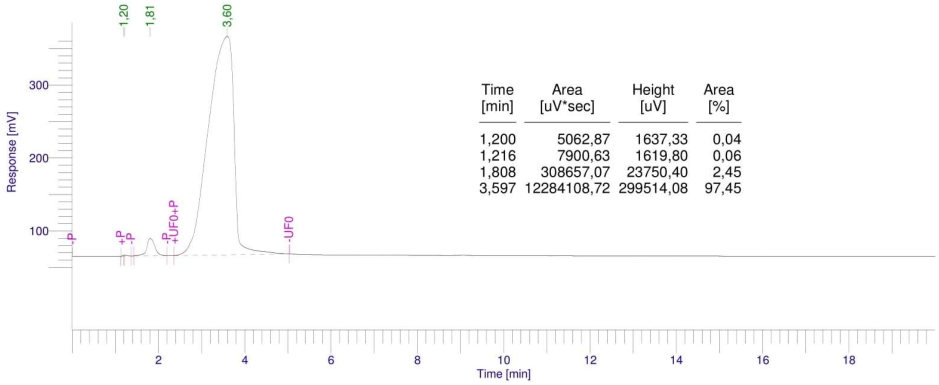

Time [min]	Area [uV*sec]	Height [uV]	Area [%]
1,200	5062,87	1637,33	0,04
1,216	7900,63	1619,80	0,06
1,808	308657,07	23750,40	2,45
3,597	12284108,72	299514,08	97,45

Entry 10:

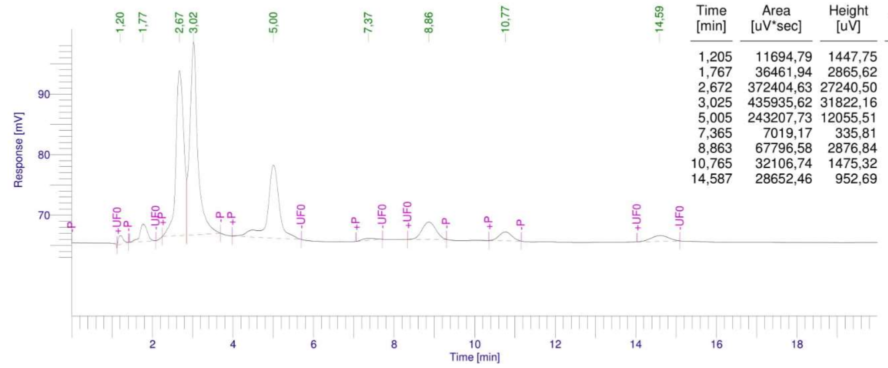

Time [min]	Area [uV*sec]	Height [uV]	Area [%]
1,205	11694,79	1447,75	0,95
1,767	36461,94	2865,62	2,95
2,672	372404,63	27240,50	30,15
3,025	435935,62	31822,16	35,29
5,005	243207,73	12055,51	19,69
7,365	7019,17	335,81	0,57
8,863	67796,58	2876,84	5,49
10,765	32106,74	1475,32	2,60
14,587	28652,46	952,69	2,32

Entry 11:

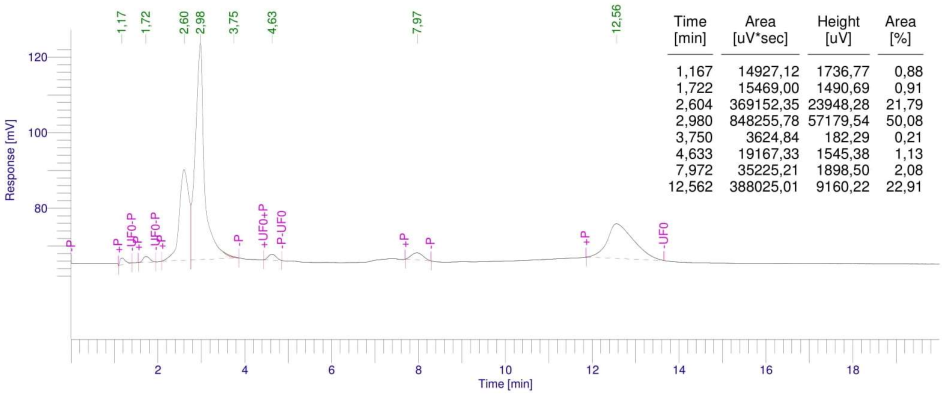

Time [min]	Area [uV*sec]	Height [uV]	Area [%]
1,167	14927,12	1736,77	0,88
1,722	15469,00	1490,69	0,91
2,604	369152,35	23948,28	21,79
2,980	848255,78	57179,54	50,08
3,750	3624,84	182,29	0,21
4,633	19167,33	1545,38	1,13
7,972	35225,21	1898,50	2,08
12,562	388025,01	9160,22	22,91

Entry 12:

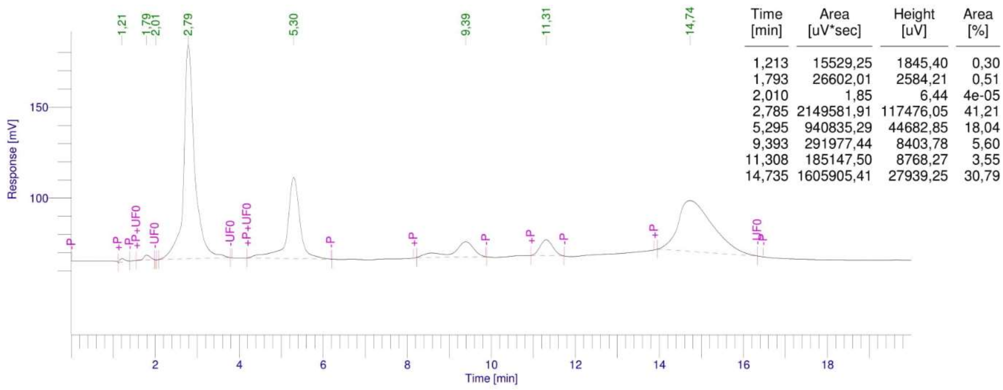

Time [min]	Area [uV*sec]	Height [uV]	Area [%]
1,213	15529,25	1845,40	0,30
1,793	26602,01	2584,21	0,51
2,010	1,85	6,44	4e-05
2,785	2149581,91	117476,05	41,21
5,295	940835,29	44682,85	18,04
9,393	291977,44	8403,78	5,60
11,308	185147,50	8768,27	3,55
14,735	1605905,41	27939,25	30,79

Entry 13:

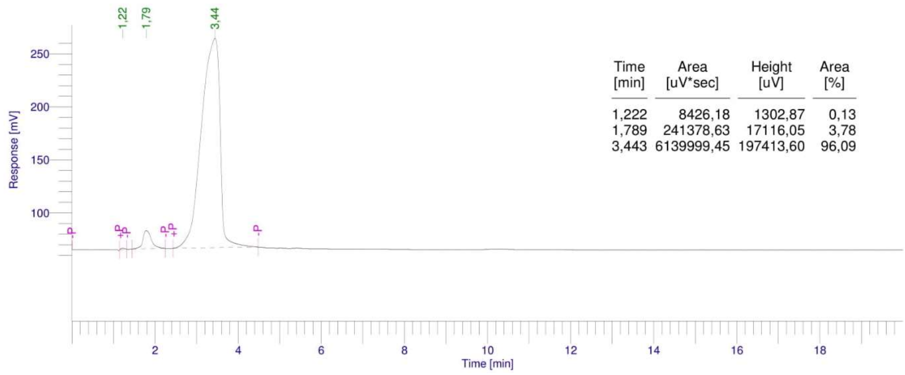

Time [min]	Area [uV*sec]	Height [uV]	Area [%]
1,222	8426,18	1302,87	0,13
1,789	241378,63	17116,05	3,78
3,443	6139999,45	197413,60	96,09

Entry 14:

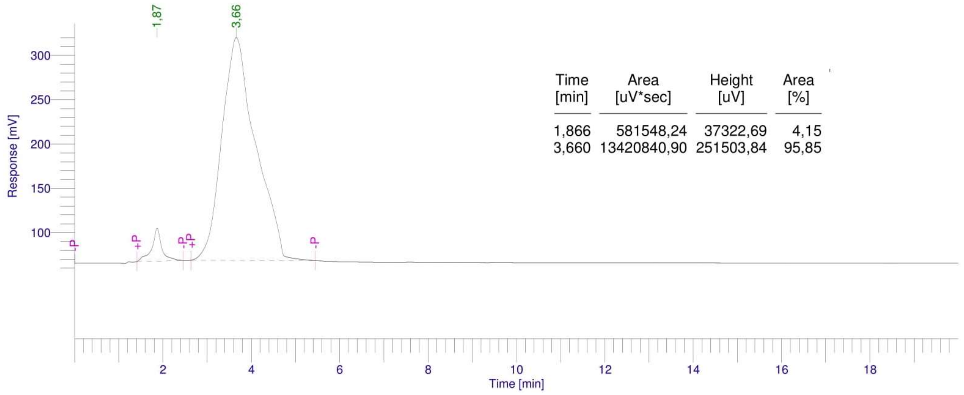

Time [min]	Area [uV*sec]	Height [uV]	Area [%]
1,866	581548,24	37322,69	4,15
3,660	13420840,90	251503,84	95,85

Entry 15:

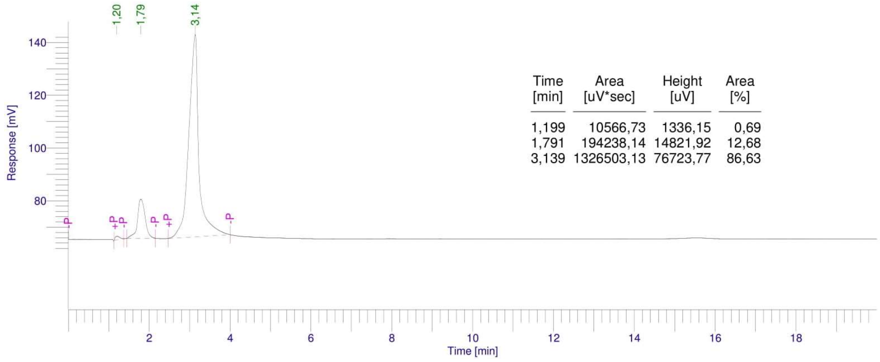

Time [min]	Area [uV*sec]	Height [uV]	Area [%]
1,199	10566,73	1336,15	0,69
1,791	194238,14	14821,92	12,68
3,139	1326503,13	76723,77	86,63

Entry 16:

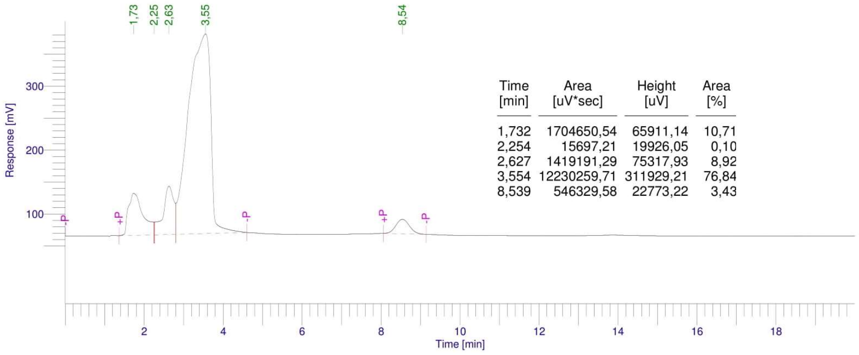

Time [min]	Area [uV*sec]	Height [uV]	Area [%]
1,732	1704650,54	65911,14	10,71
2,254	15697,21	19926,05	0,10
2,627	1419191,29	75317,93	8,92
3,554	12230259,71	311929,21	76,84
8,539	546329,58	22773,22	3,43

Entry 17:

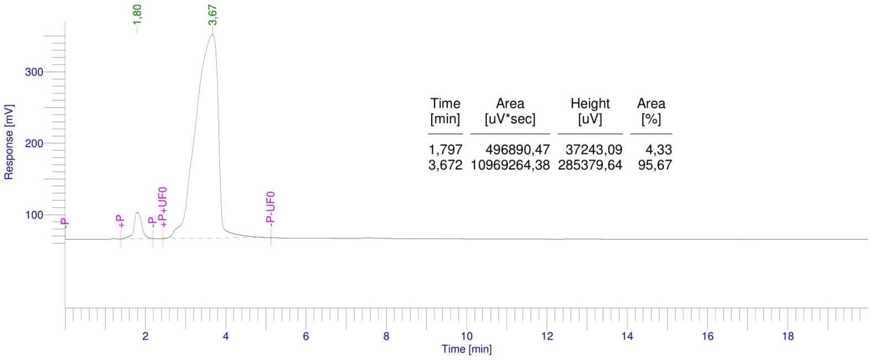

Time [min]	Area [uV*sec]	Height [uV]	Area [%]
1,797	496890,47	37243,09	4,33
3,672	10969264,38	285379,64	95,67

5b standard:

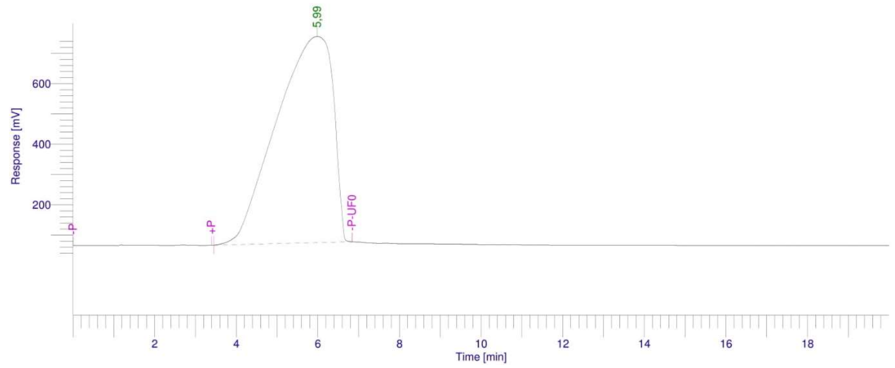

Entry 18:

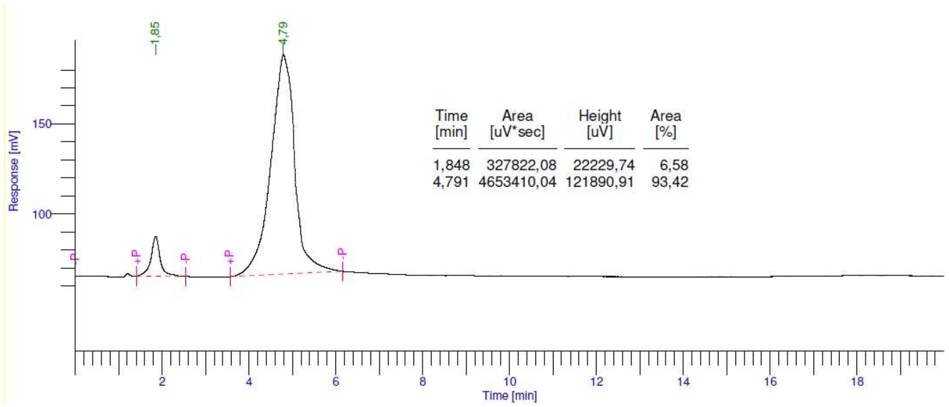

Time [min]	Area [uV*sec]	Height [uV]	Area [%]
1,848	327822,08	22229,74	6,58
4,791	4653410,04	121890,91	93,42

Entry 19:

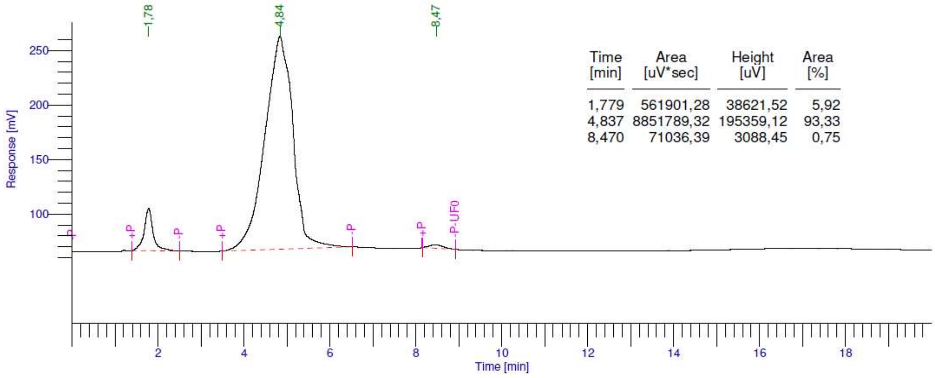

Time [min]	Area [uV*sec]	Height [uV]	Area [%]
1,779	561901,28	38621,52	5,92
4,837	8851789,32	195359,12	93,33
8,470	71036,39	3088,45	0,75

Entry 20

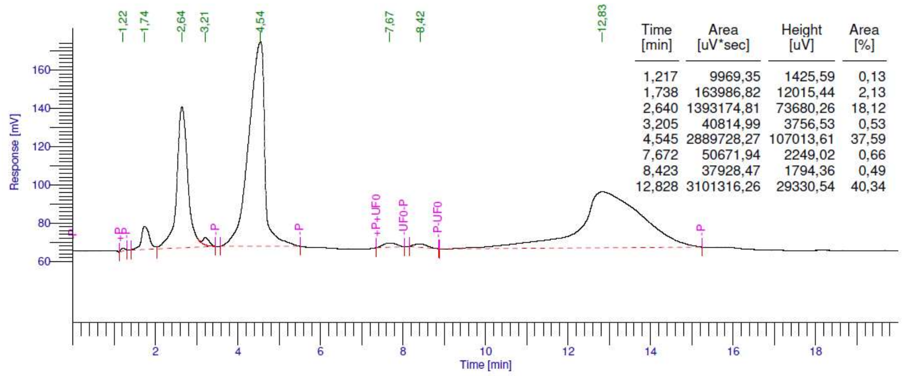

Time [min]	Area [uV*sec]	Height [uV]	Area [%]
1,217	9969,35	1425,59	0,13
1,738	163986,82	12015,44	2,13
2,640	1393174,81	73680,26	18,12
3,205	40814,99	3756,53	0,53
4,545	2889728,27	107013,61	37,59
7,672	50671,94	2249,02	0,66
8,423	37928,47	1794,36	0,49
12,828	3101316,26	29330,54	40,34

Entry 21:

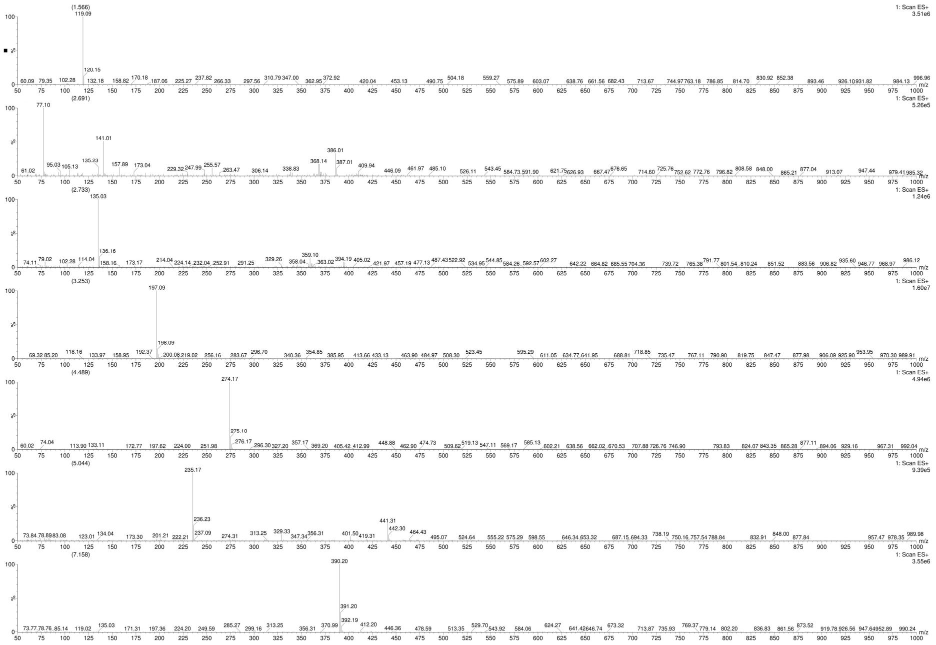

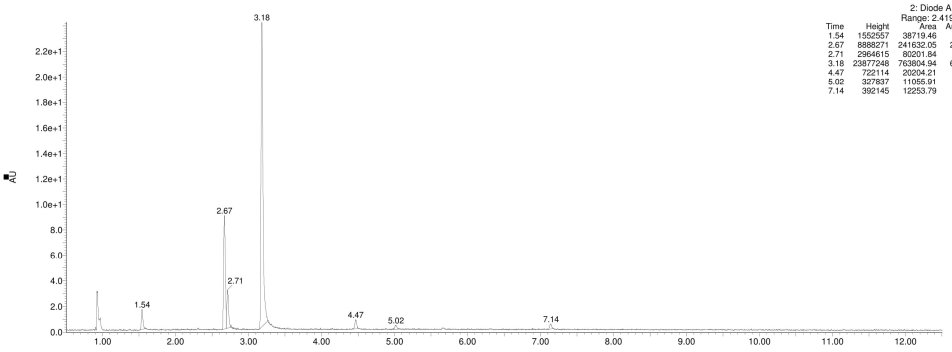

2: Diode Array
Range: 2.419e+1

Time	Height	Area	Area%
1.54	1552557	38719.46	3.32
2.67	8888271	241632.05	20.69
2.71	2964615	80201.84	6.87
3.18	23877248	763804.94	65.40
4.47	722114	20204.21	1.73
5.02	327837	11055.91	0.95
7.14	392145	12253.79	1.05

Entry 22:

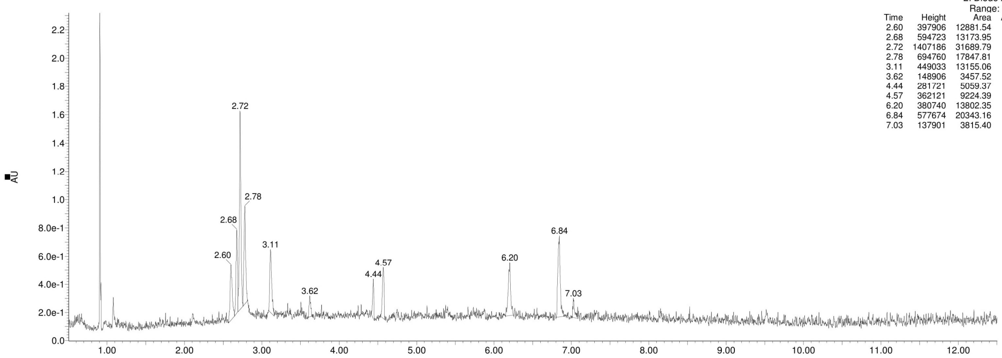

2: Diode Array
Range: 2.251

Time	Height	Area	Area%
2.60	397906	12881.54	8.92
2.68	594723	13173.95	9.12
2.72	1407186	31689.79	21.94
2.78	694760	17847.81	12.36
3.11	449033	13155.06	9.11
3.62	148906	3457.52	2.39
4.44	281721	5059.37	3.50
4.57	362121	9224.39	6.39
6.20	380740	13802.35	9.56
6.84	577674	20343.16	14.08
7.03	137901	3815.40	2.64

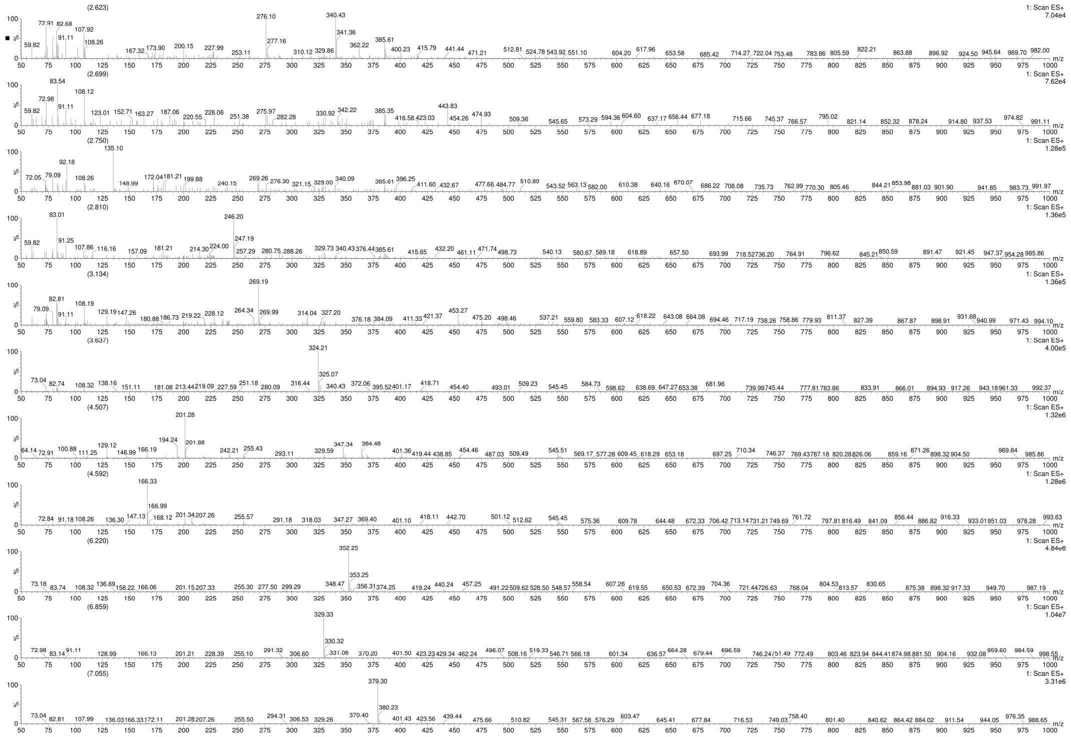

(2.623)
1: Scan ES+
7.04e4
(2.699)
1: Scan ES+
7.62e4
(2.750)
1: Scan ES+
1.28e5
(2.810)
1: Scan ES+
1.36e5
(3.134)
1: Scan ES+
1.36e5
(3.637)
1: Scan ES+
4.00e5
(4.507)
1: Scan ES+
1.32e6
(4.592)
1: Scan ES+
1.28e6
(6.220)
1: Scan ES+
4.84e6
(6.859)
1: Scan ES+
1.04e7
(7.055)
1: Scan ES+
3.31e6
m/z

Entry 23:

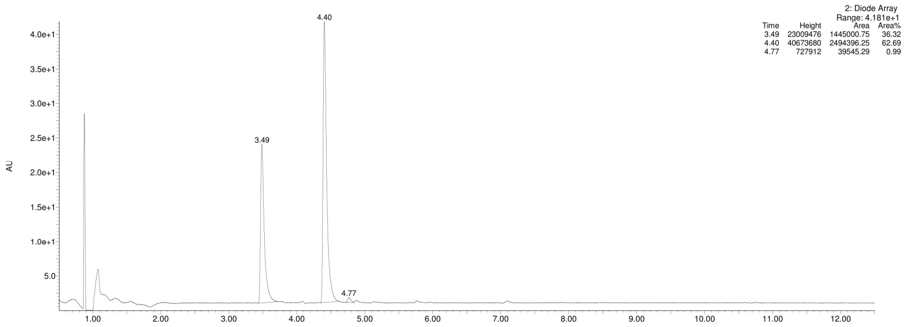

2: Diode Array
Range: 4.181e+1
Time Height Area Area%
3.49 23009476 1445000.75 36.32
4.40 40673680 2494396.25 62.69
4.77 727912 39545.29 0.99
AU
3.49
4.40
4.77

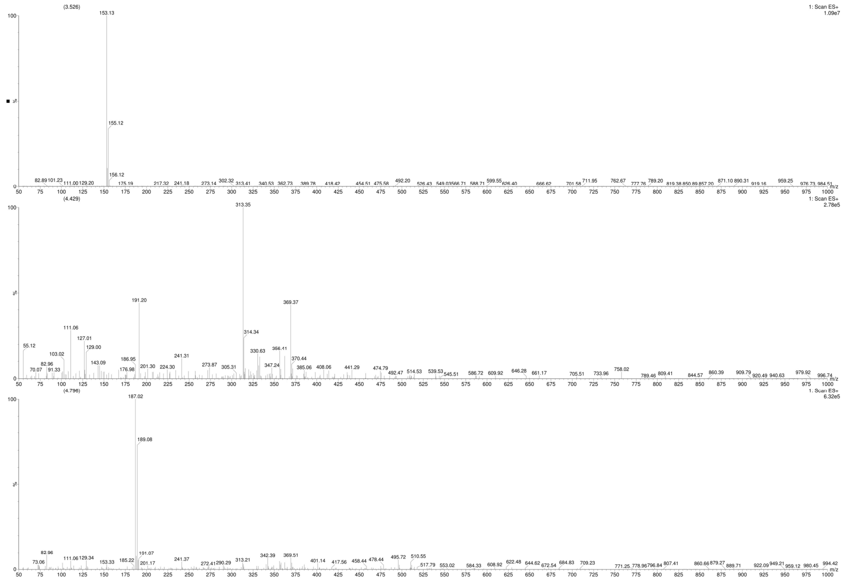

(3.526)
1: Scan ES+
1.09e7
(4.429)
1: Scan ES+
2.78e5
(4.796)
1: Scan ES+
6.32e5
m/z

Entry 24:

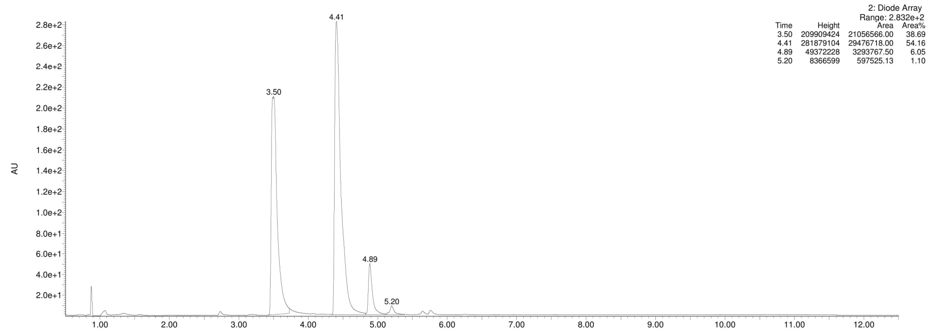

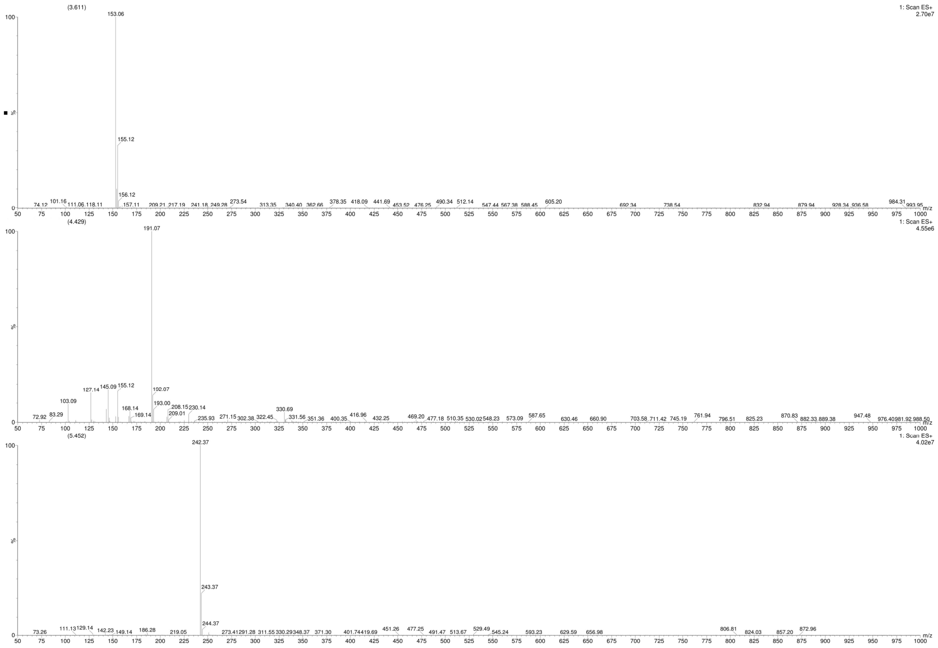

6c standard:

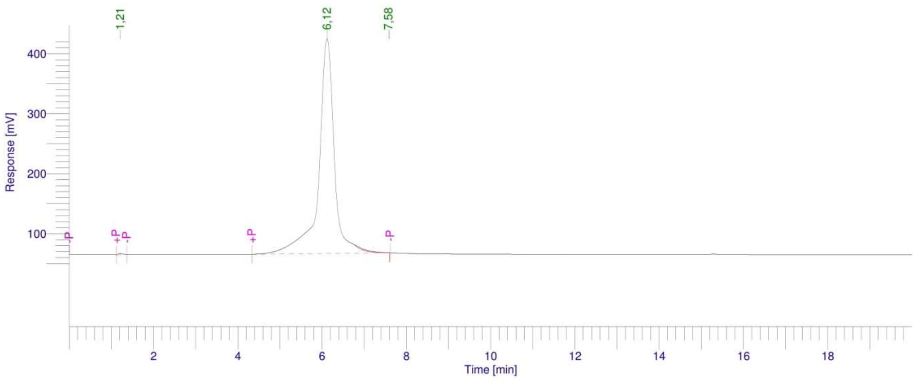

Entry 25:

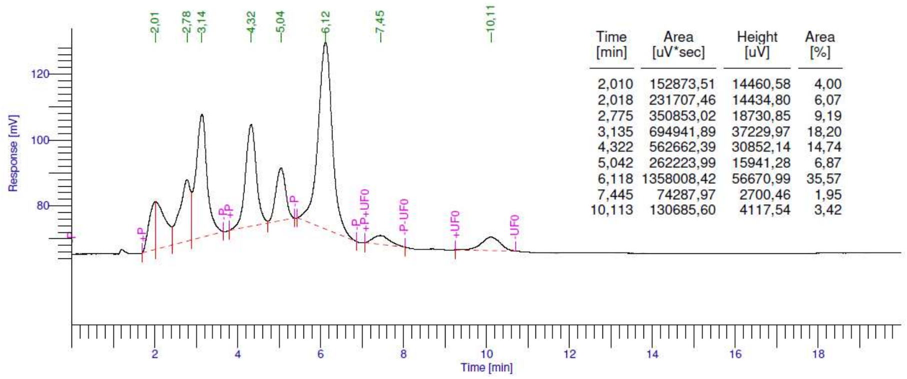

Time [min]	Area [uV*sec]	Height [uV]	Area [%]
2,010	152873,51	14460,58	4,00
2,018	231707,46	14434,80	6,07
2,775	350853,02	18730,85	9,19
3,135	694941,89	37229,97	18,20
4,322	562662,39	30852,14	14,74
5,042	262223,99	15941,28	6,87
6,118	1358008,42	56670,99	35,57
7,445	74287,97	2700,46	1,95
10,113	130685,60	4117,54	3,42

Entry 26:

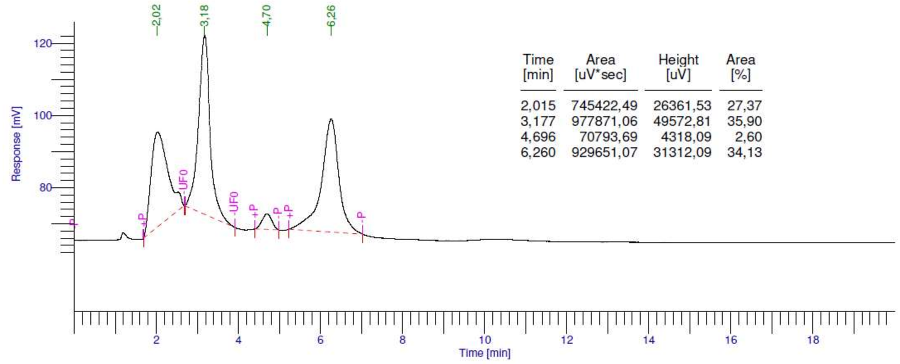

Time [min]	Area [uV*sec]	Height [uV]	Area [%]
2,015	745422,49	26361,53	27,37
3,177	977871,06	49572,81	35,90
4,696	70793,69	4318,09	2,60
6,260	929651,07	31312,09	34,13

Entry 27:

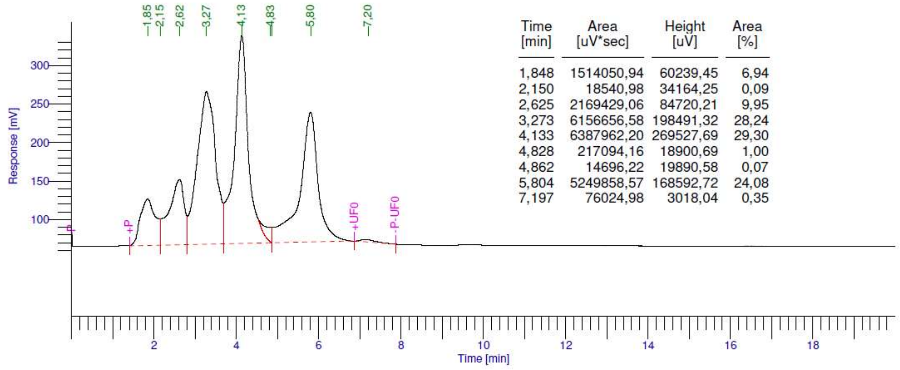

Time [min]	Area [uV*sec]	Height [uV]	Area [%]
1,848	1514050,94	60239,45	6,94
2,150	18540,98	34164,25	0,09
2,625	2169429,06	84720,21	9,95
3,273	6156656,58	198491,32	28,24
4,133	6387962,20	269527,69	29,30
4,828	217094,16	18900,69	1,00
4,862	14696,22	19890,58	0,07
5,804	5249858,57	168592,72	24,08
7,197	76024,98	3018,04	0,35

Entry 28:

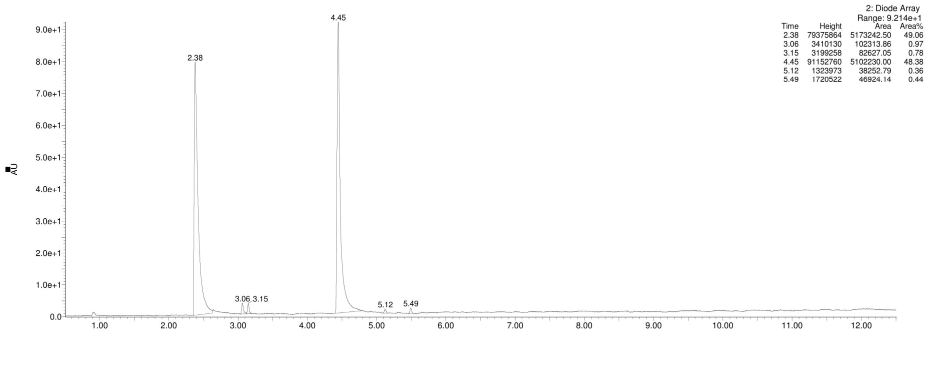

Time	Height	Area	Area%
2.38	79375864	5173242.50	49.06
3.06	3410130	102313.86	0.97
3.15	3199258	82627.05	0.78
4.45	91152760	5102230.00	48.38
5.12	1323973	38252.79	0.36
5.49	1720522	46924.14	0.44

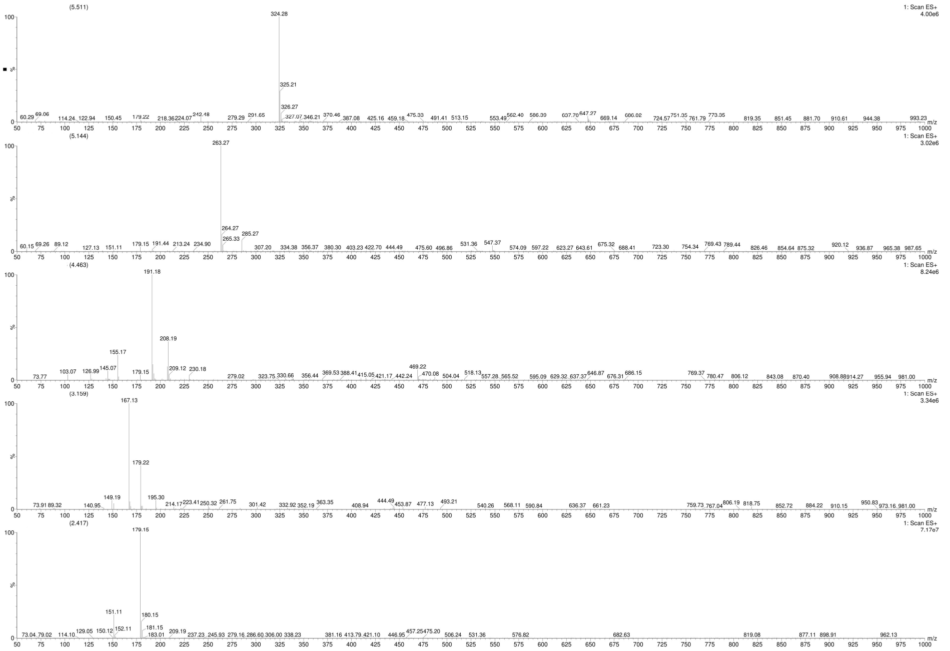

Entry 29:

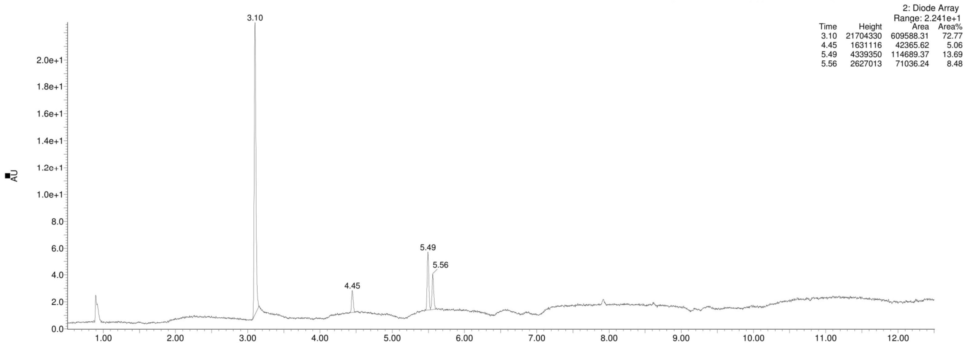

2: Diode Array
Range: 2.241e+1
Time Height Area Area%
3.10 21704330 609588.31 72.77
4.45 1631116 42365.62 5.06
5.49 4339350 114689.37 13.69
5.56 2627013 71036.24 8.48

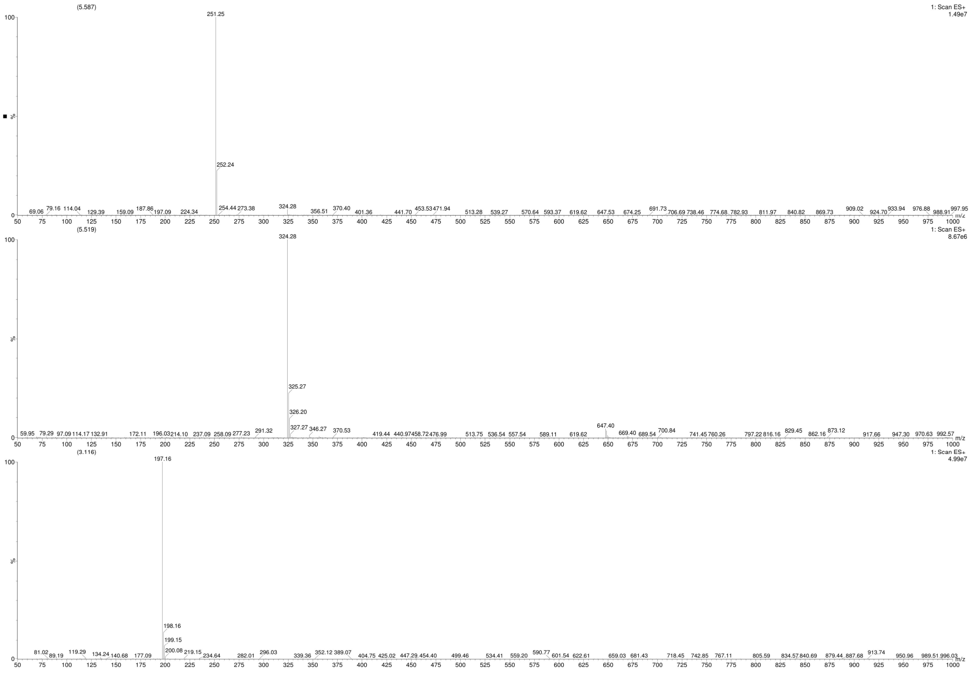

Entry 30:

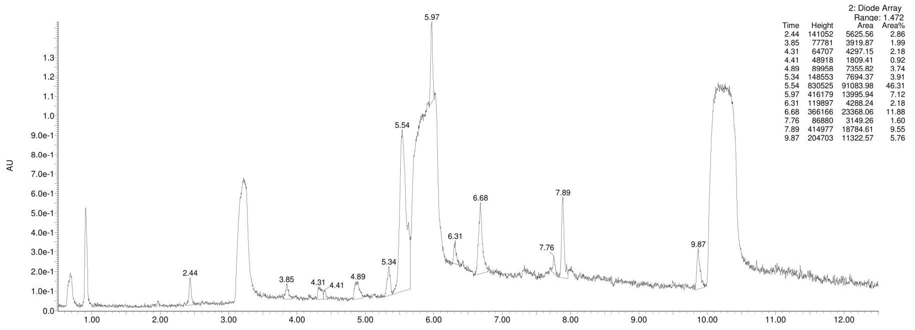

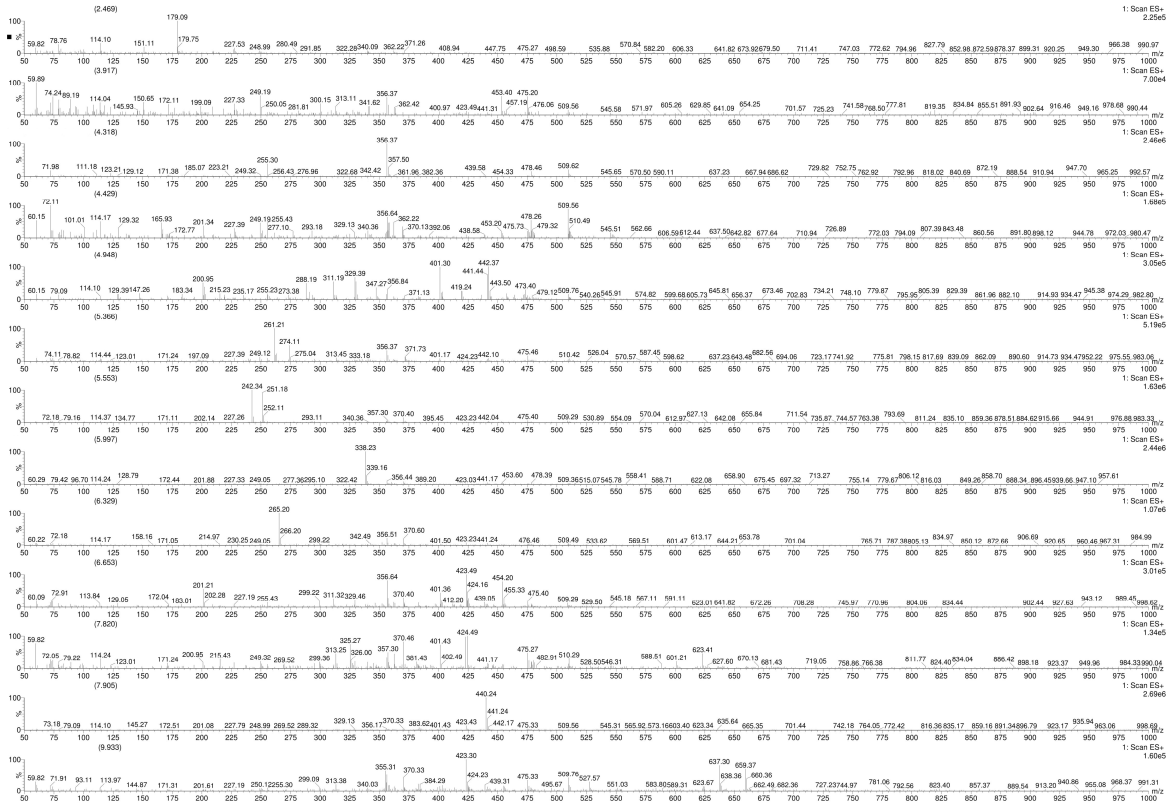

5c standard:

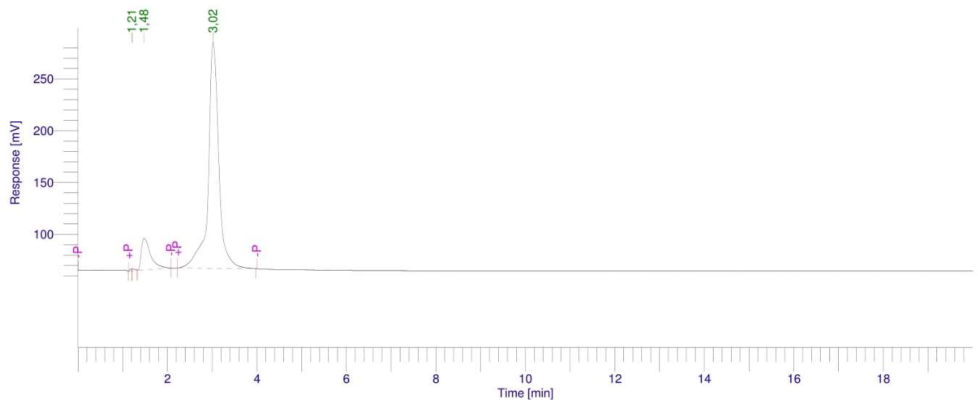

Entry 31:

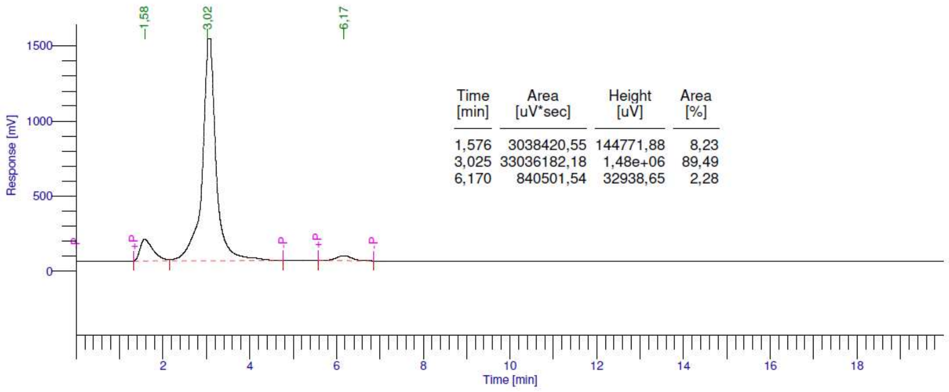

Time [min]	Area [uV*sec]	Height [uV]	Area [%]
1,576	3038420,55	144771,88	8,23
3,025	33036182,18	1,48e+06	89,49
6,170	840501,54	32938,65	2,28

Entry 32:

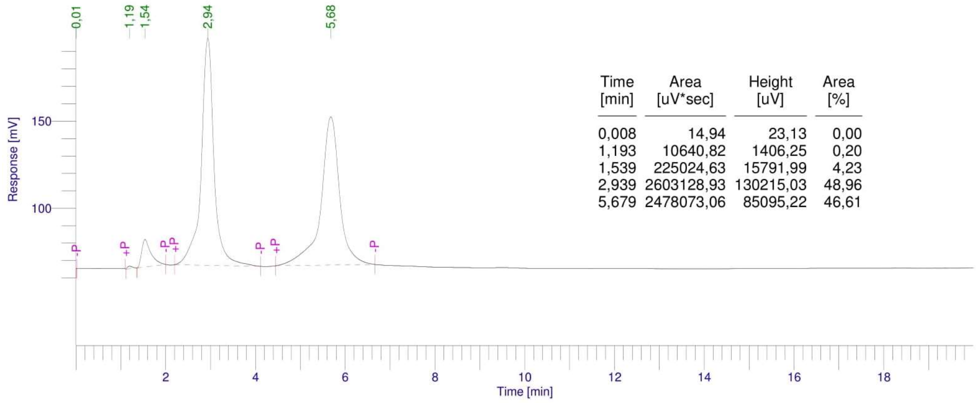

Time [min]	Area [uV*sec]	Height [uV]	Area [%]
0,008	14,94	23,13	0,00
1,193	10640,82	1406,25	0,20
1,539	225024,63	15791,99	4,23
2,939	2603128,93	130215,03	48,96
5,679	2478073,06	85095,22	46,61

Entry 33:

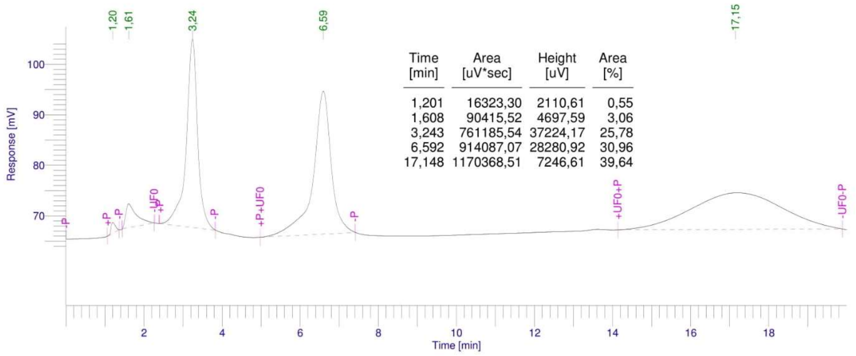

Time [min]	Area [uV*sec]	Height [uV]	Area [%]
1,201	16323,30	2110,61	0,55
1,608	90415,52	4697,59	3,06
3,243	761185,54	37224,17	25,78
6,592	914087,07	28280,92	30,96
17,148	1170368,51	7246,61	39,64

Entry 34

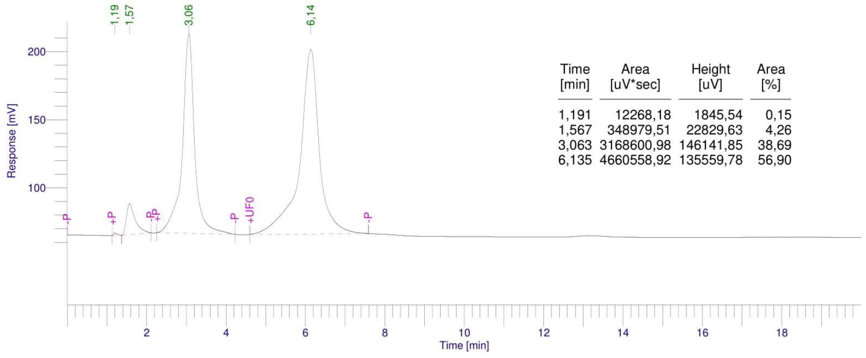

Time [min]	Area [uV*sec]	Height [uV]	Area [%]
1,191	12268,18	1845,54	0,15
1,567	348979,51	22829,63	4,26
3,063	3168600,98	146141,85	38,69
6,135	4660558,92	135559,78	56,90

10e standard:

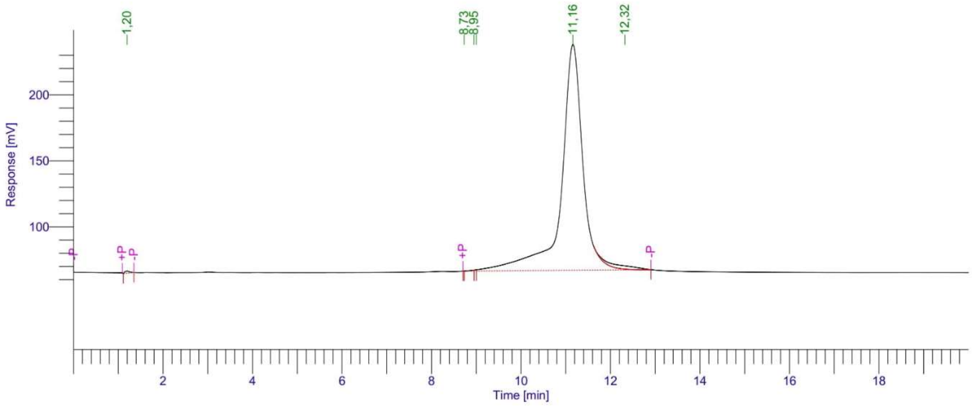

Entry 35:

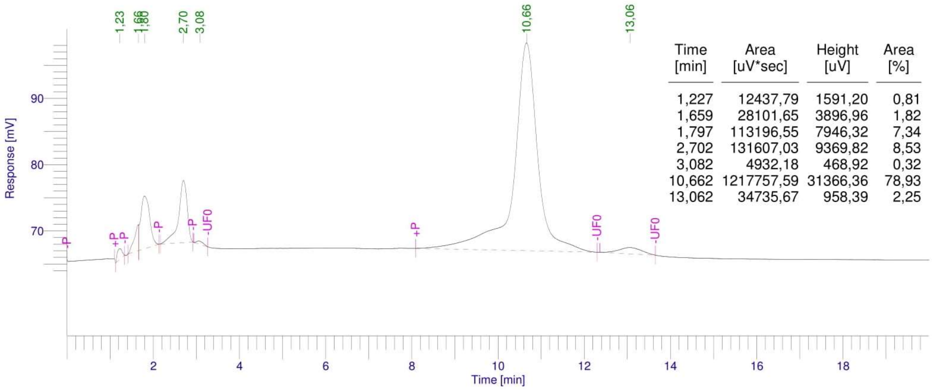

Time [min]	Area [uV*sec]	Height [uV]	Area [%]
1,227	12437,79	1591,20	0,81
1,659	28101,65	3896,96	1,82
1,797	113196,55	7946,32	7,34
2,702	131607,03	9369,82	8,53
3,082	4932,18	468,92	0,32
10,662	1217757,59	31366,36	78,93
13,062	34735,67	958,39	2,25

Entry 36:

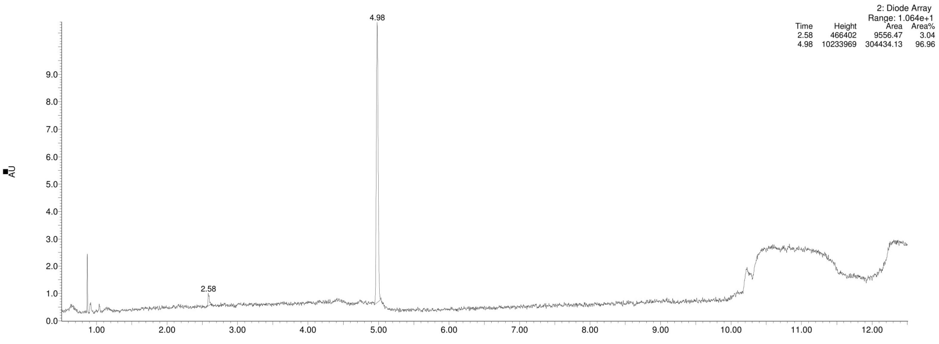

Time	Height	Area	Area%
2.58	466402	9556.47	3.04
4.98	10233969	304434.13	96.96

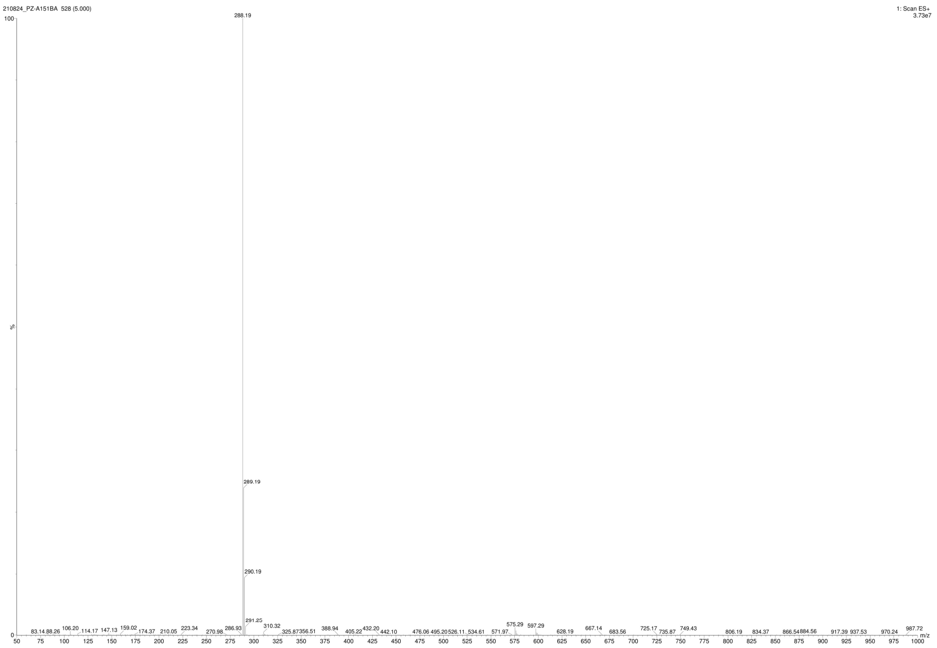
210824_PZ-A151BA 528 (5.000)
1: Scan ES+
3.73e7
288.19
289.19
290.19
m/z

Entry 38:

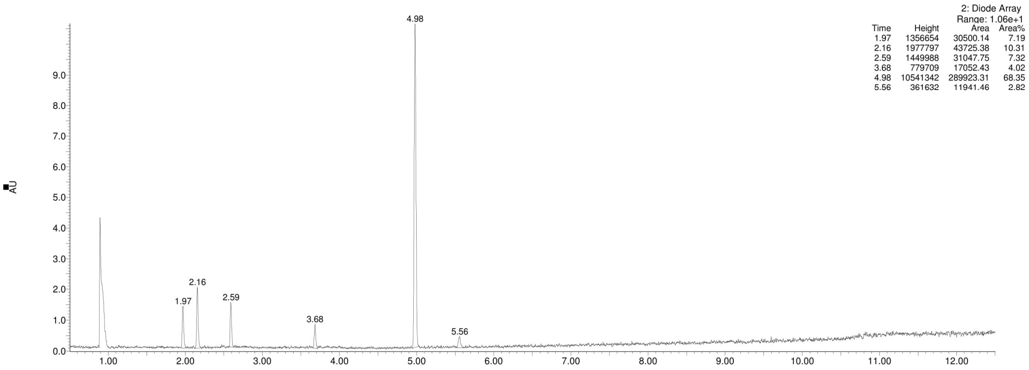
2: Diode Array
Range: 1.06e+1
Time Height Area Area%
1.97 1356654 30500.14 7.19
2.16 1977797 43725.38 10.31
2.59 1449988 31047.75 7.32
3.68 779709 17052.43 4.02
4.98 10541342 289923.31 68.35
5.56 361632 11941.46 2.82
AU
4.98
1.97
2.16
2.59
3.68
5.56

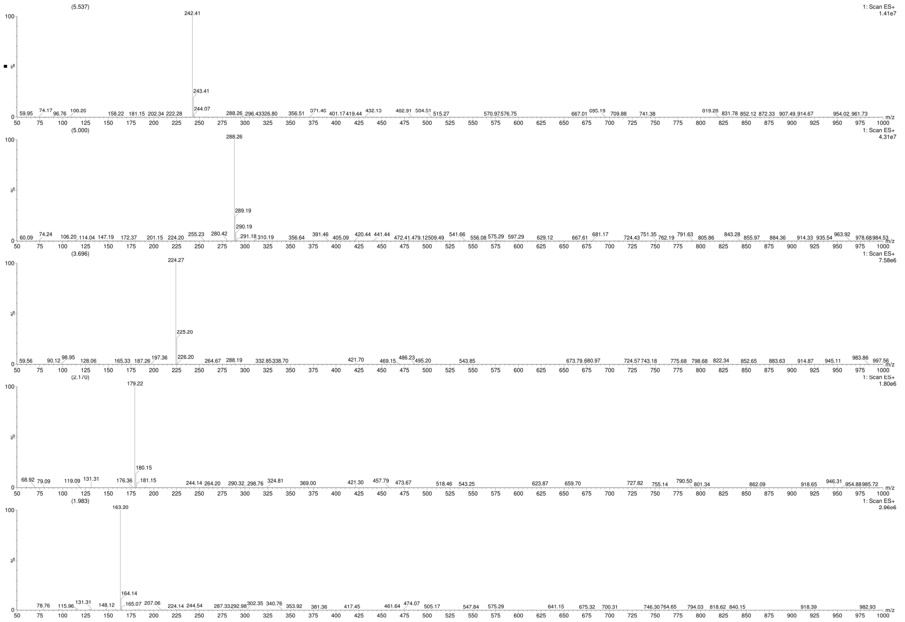
(5.537)
1: Scan ES+
1.41e7
242.41
243.41
(5.000)
1: Scan ES+
4.31e7
288.26
289.19
290.19
(3.696)
1: Scan ES+
7.58e6
224.27
225.20
(2.170)
1: Scan ES+
1.80e6
179.22
180.15
(1.983)
1: Scan ES+
2.96e6
163.20
164.14
m/z

Entry 39:

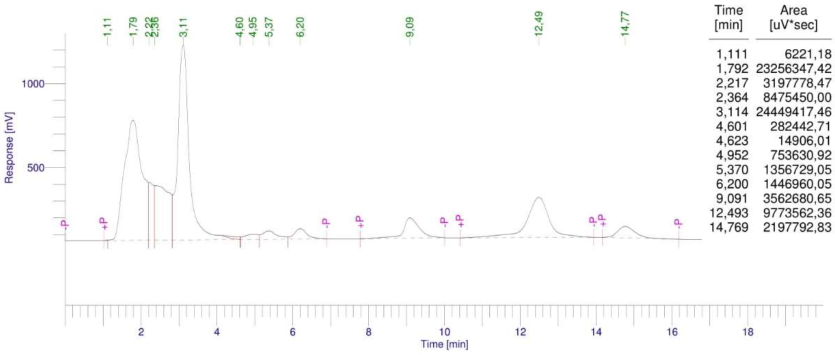

Time [min]	Area [uV*sec]	Height [uV]	Area [%]
1,111	6221,18	2018,86	0,01
1,792	23256347,42	714364,26	29,52
2,217	3197778,47	344032,18	4,06
2,364	8475450,00	325341,96	10,76
3,114	24449417,46	1,16e+06	31,04
4,601	282442,71	15595,36	0,36
4,623	14906,01	15724,63	0,02
4,952	753630,92	30792,41	0,96
5,370	1356729,05	49621,48	1,72
6,200	1446960,05	62027,57	1,84
9,091	3562680,65	121401,81	4,52
12,493	9773562,36	240071,93	12,41
14,769	2197792,83	68043,08	2,79

Entry 40:

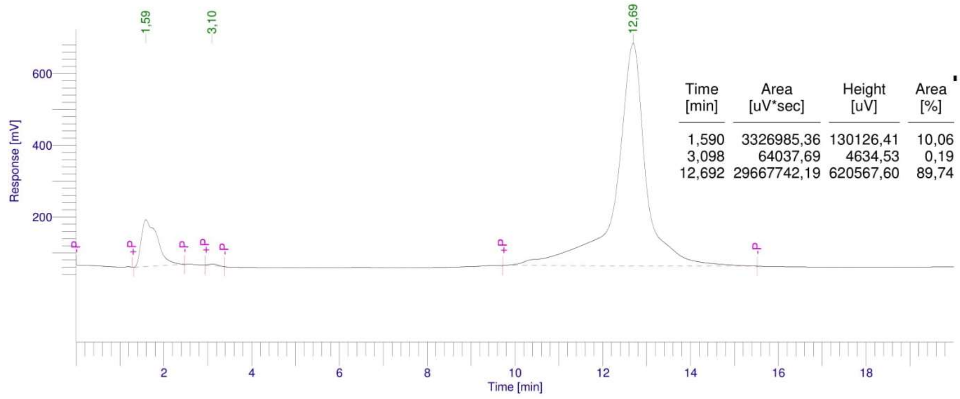

Time [min]	Area [uV*sec]	Height [uV]	Area [%]
1,590	3326985,36	130126,41	10,06
3,098	64037,69	4634,53	0,19
12,692	29667742,19	620567,60	89,74

Entry 41:

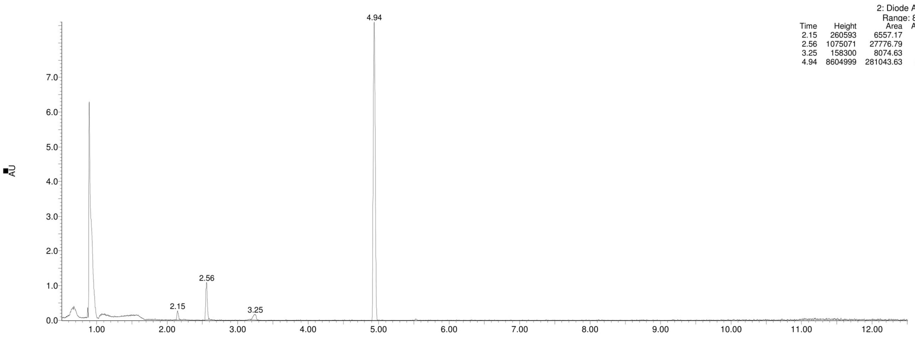

2: Diode Array
Range: 8.605

Time	Height	Area	Area%
2.15	260593	6557.17	2.03
2.56	1075071	27776.79	8.59
3.25	158300	8074.63	2.50
4.94	8604999	281043.63	86.89

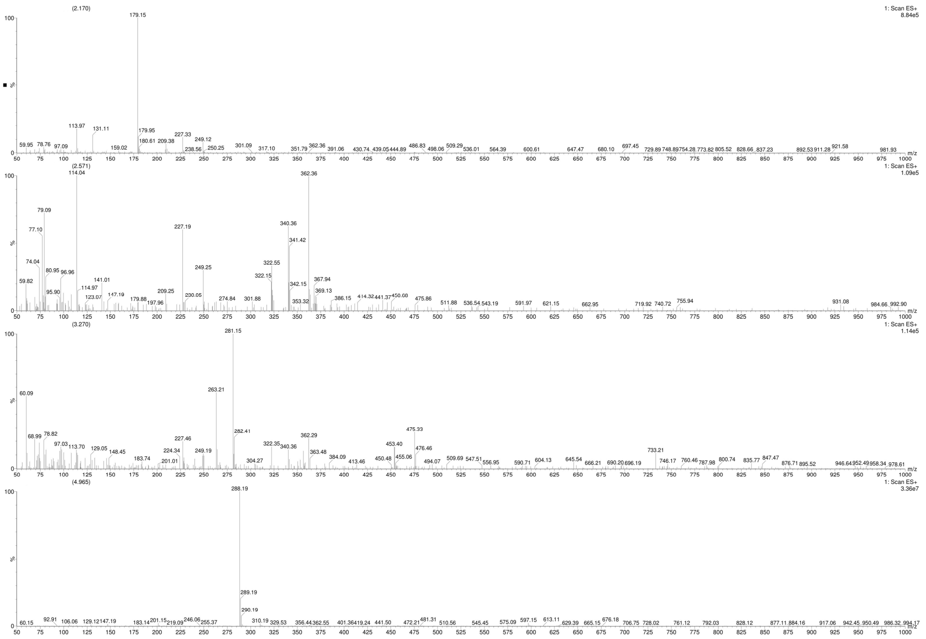

Entry 42:

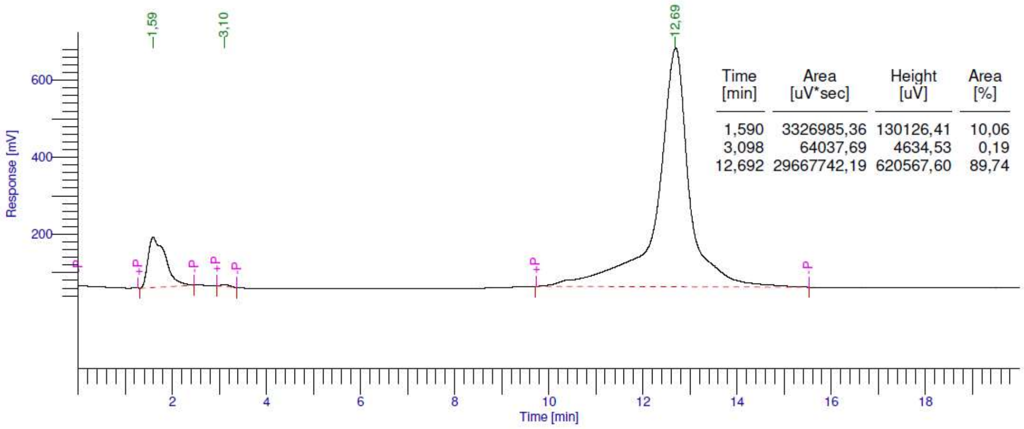

Entry 43:

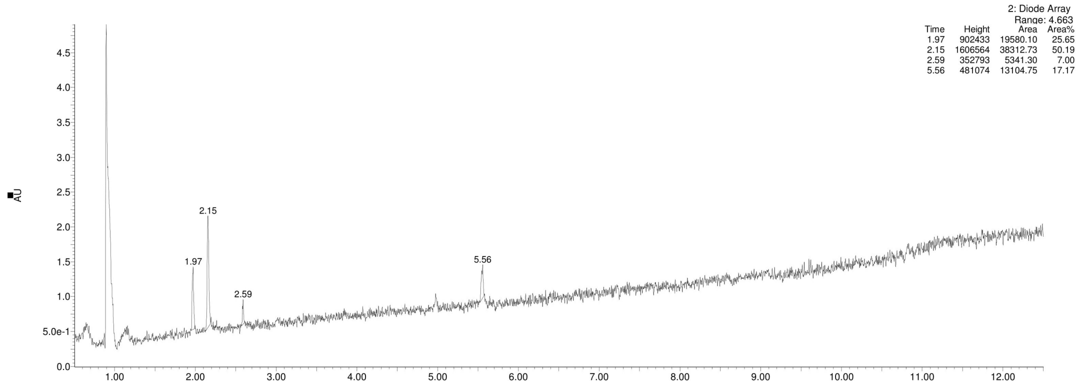

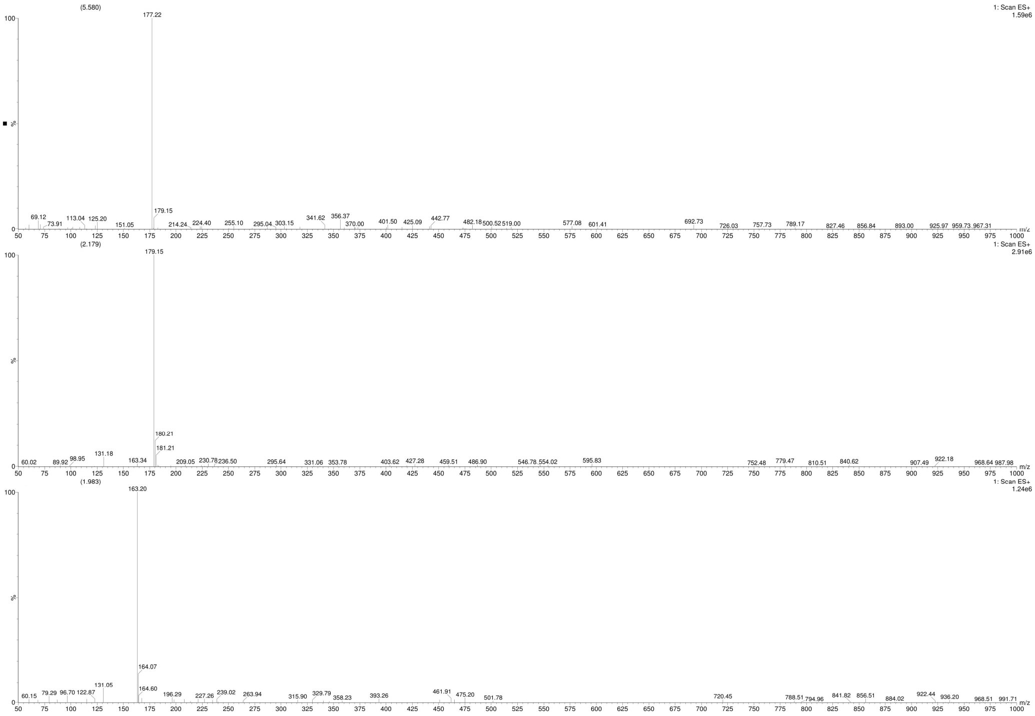

Entry 44:

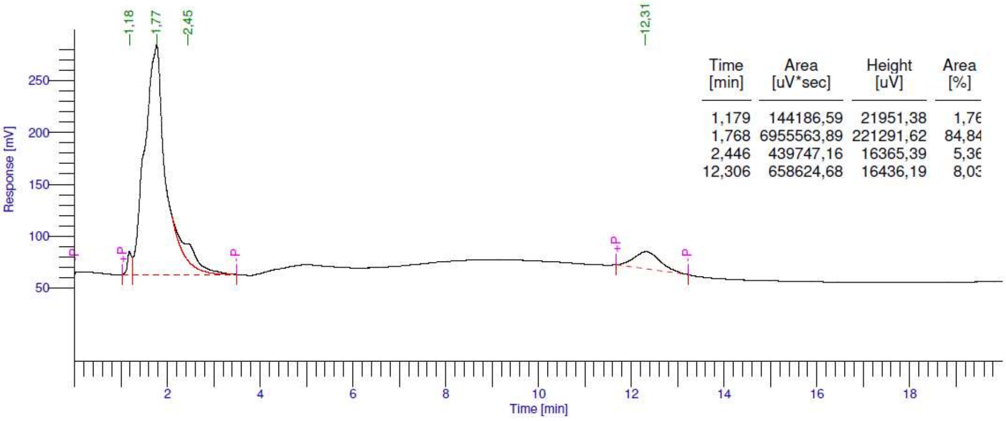

Time [min]	Area [uV*sec]	Height [uV]	Area [%]
1,179	144186,59	21951,38	1,76
1,768	6955563,89	221291,62	84,84
2,446	439747,16	16365,39	5,36
12,306	658624,68	16436,19	8,03
